# Target Fidelity and Failure: Structure–Activity Relationship of High-Molecular-Mass Penicillin-Binding Proteins (HMM-PBPs) in Refractory *Granulicatella adiacens* Endocarditis

**DOI:** 10.3390/antibiotics15020168

**Published:** 2026-02-05

**Authors:** Paola Conti, Alberto Pagotto, Sebastiano A. Fortuna, Alessandra Giardina, Grete F. Privitera, Ester Rosa, Assunta Sartor, Carlo Tascini, Floriana Campanile

**Affiliations:** 1 Department of Biomedical and Biotechnological Sciences, Section of Microbiology, University of Catania, 95123 Catania, Italy; paolacontias@hotmail.com (P.C.); s.albertofortuna@gmail.com (S.A.F.); alessandragiardina27@gmail.com (A.G.); ester.rosa@unict.it (E.R.); 2 Department of Medical Biotechnologies, University of Siena, 53100 Siena, Italy; 3 Infectious Diseases Division, Azienda Sanitaria Universitaria Friuli Centrale (ASUFC), 33100 Udine, Italy; alberto.pagotto@asufc.sanita.fvg.it (A.P.); carlo.tascini@asufc.sanita.fvg.it (C.T.); 4 Department of Biotechnological and Applied Clinical Sciences (DISCAB), University of L’Aquila, 67100 L’Aquila, Italy; 5 Bioinformatics Unit, Department of Clinical and Experimental Medicine, University of Catania, 95123 Catania, Italy; grete.privitera@unict.it; 6 Microbiology Unit, Udine University Hospital, 33100 Udine, Italy; assunta.sartor@asufc.sanita.fvg.it; 7 Department of Medicine (DMED), University of Udine, 33100 Udine, Italy

**Keywords:** *Granulicatella adiacens*, infective endocarditis, penicillin non-susceptible, double beta-lactam, penicillin-binding proteins, PBP affinity, Bocillin

## Abstract

**Background/Objectives**: *Granulicatella adiacens* infective endocarditis is conventionally managed with penicillin, ampicillin, or ceftriaxone in combination with gentamicin, although double beta-lactam regiments have been proposed a safer alternative to reduce aminoglycoside-associated nephrotoxicity. To date, the High-Molecular-Mass Penicillin-Binding Proteins (HMM-PBPs) of *G. adiacens* and their affinities for beta-lactam antibiotics have not been previously characterized. This study investigated the HMM-PBP profile of *G. adiacens*, with particular interest on sequence alterations and beta-lactam binding properties, both as single agents and in combination. **Methods:** Beta-lactam activity, synergistic interactions and PBP binding affinities were evaluated in a clinical isolate (IS 48) and compared with those in the reference strain ATCC 49175. Binding of PBPs to ampicillin, ceftriaxone, and ceftobiprole, alone or in combination, was investigated by Bocillin-FL labeling. PBP homology and conserved active-sites motifs were assessed by sequence alignment, and *pbp* gene mutations were identified by whole-genome sequencing. **Results:** The clinical isolate was non-susceptible to ampicillin, resistant to ceftriaxone and exhibited higher minimum inhibitory concentrations (MICs) for ceftobiprole relative to the fully susceptible ATCC reference strain. Five HMM PBPs with high enterococcal homology, were identified. In the IS 48 isolate, the class A PBP showed distinct amino acid substitutions in proximity to the catalytic centers. Despite these alterations, PBP1A and PBP2A were strongly inhibited by the tested beta-lactams, whereas PBP2 and PBP2B demonstrated low acylation rates. Combination of ampicillin with either ceftobiprole or ceftriaxone resulted in enhanced acylation of the three bifunctional HMM PBPs compared with monotreatment. IC_50_ values were consistently higher for the IS 48 clinical isolate, suggesting decreased target availability and/or reduced beta-lactam affinity under clinical conditions. **Conclusions:** The resistance phenotype of *G. adiacens* clinical isolate appears to be primarily associated with altered PBP beta-lactam interactions. Nonetheless, beta-lactam combination regimes remain effective by achieving substantial inhibition of key HMM-PBPs involved in peptidoglycan synthesis, thereby supporting the rationale for dual beta-lactam therapy in this setting.

## 1. Introduction

*Granulicatella adiacens* is a nutritionally demanding (fastidious) organism, originally classified among the Nutritionally Variant Streptococci (NVS), that typically colonizes the humans oral, gastrointestinal and genitourinary tracts and able to cause invasive infections such as bacteraemia, osteoarticular infections and infective endocarditis (IE) [1].

Owing to its requirements for multiple growth supplements, conventional cultures are frequently negative, thereby delaying diagnosis, prolonging the course of disease and contributing to a high rate of complications. Among all cases of streptococcal IE, 5–6% are estimated to be attributable to NVS; however, this proportion is likely underestimated because of the diagnostic difficulties mentioned above. Even with guideline-concordant appropriate antimicrobial therapy, treatment failure is common and surgical intervention may be required.

*G. adiacens* IE is frequently associated with valvular vegetations (reported in approximately 64% of cases), a feature that has been linked to its relatively slower growth rate that may reduce the bactericidal activity of beta-lactam antibiotics. Relapse after apparently appropriate therapy is not uncommon, even when isolates exhibit high in vitro susceptibility. Reported mortality ranges from 17 to 27%, exceeding that observed in IE caused by enterococci and streptococci. Pre-existing valvular pathologies are common predisposing factors; nevertheless, *G. adiacens* IE can also occur in healthy individuals often following dental procedures, such as tooth extraction, which facilitate bacterial translocation from the oral cavity [1,2,3,4,5].

Current recommendations from the American Heart Association (AHA) and the European Society of Cardiology (ESC) for NVS IE advocate treatment with a beta-lactam, such as penicillin (P), ampicillin (AMP), or ceftriaxone (CRO) in combination with gentamycin, or with vancomycin in patients with contraindications to aminoglycosides [6,7]. Given the substantial risk of aminoglycoside-associated nephrotoxicity, several clinical reports have suggested that dual beta-lactam therapy may constitute an effective alternative treatment for IE sustained by *G. adiacens*.

To avoid nephrotoxicity, monotherapy with ceftriaxone was initially employed as beta-lactam-only approach; however, this approach was proved to be clinically insufficient, indicating the need for optimization of treatment regiments [8]. Khan et al. [9] demonstrated the in vitro synergistic activity of ampicillin plus ceftriaxone against a susceptible *G. adiacens* strain causing IE, and successfully treated a patient at high risk of nephrotoxicity using intravenously (i.v.) ampicillin 2 g every 6 h combined with ceftriaxone 2 g every 24 h. Similarly, Pagotto et al. [10] reported a case of IE sustained by *G. adiacens* successfully treated with 4 g ampicillin i.v. every 6 h plus ceftriaxone 2 g i.v. every 12 h. Both studies documented synergistic activity with dual beta-lactam therapy, comparable to that observed in *Enterococcus faecalis*, where the effect is attributed to inhibition of a broader spectrum of Penicillin-Binding Proteins (PBPs). On this basis, the authors suggested the adoption of comparable combination regiments for *G. adiacens* IE.

Although NVS generally exhibit low Minimal Inhibitory Concentrations (MICs) for many antibiotic classes, the emergence of beta-lactam resistance in *G. adiacens* is of increasing concern. A substantial proportion of *G. adiacens* isolates displayed reduced susceptibility or resistance to penicillin (MIC ≥ 0.25 mg/L). In particular, these penicillin-non-susceptible strains frequently show resistance to third-generation cephalosporins, including ceftriaxone, cefuroxime, and cefepime (MIC ≥ 4 mg/L), with a higher prevalence than that observed among penicillin-susceptible strains [11]. In addition, strains exhibit elevated MIC values to ceftaroline, a fifth-generation cephalosporin (MIC > 4 mg/L) [5].

This beta-lactam resistance phenotype is likely multifactorial, with PBPs—well-established targes of beta-lactams in other bacterial species—playing a central role. Bacteria may overcome beta-lactam activity through the production of constitutive low-affinity PBPs, the expression of alternative PBPs, or the acquisition of mutations, particularly within the three conserved catalytic motifs within the transpeptidase domain.

For instance, Methicillin-Resistant *Staphylococcus aureus* (MRSA) expresses PBP2a, an additional/alternative low-affinity transpeptidase, that confers broad beta-lactam resistance [12]. *Streptococcus pneumoniae* constitutively produces the low-affinity PBP2X, whose mosaicity together with alterations in PBP2b (transpeptidase) and PBP1a (transglycosylase and transpeptidase), is essential for the development of beta-lactam resistance [13]. Among enterococci, *Enterococcus faecalis* and *E. faecium* express low-affinity PBPs known as PBP4 and PBP5, respectively [12]. Point mutations can further decrease beta-lactam affinity [14], and increased PBP expression levels can also contribute to resistance phenotypes [15].

Despite clinical evidence supporting the use of dual beta-lactam therapy in *G. adiacens* IE, the molecular mechanisms underlying beta-lactam resistance in this species, as well as the mechanistic basis for the observed benefit of combination regimens, remain poorly understood. Data are limited regarding the PBP repertoire of *G. adiacens*, the involvement of specific sequence alterations to reduced beta-lactam susceptibility, and the interaction between individual beta-lactams and PBP targets, when used as monotherapy or in combination. A more detailed understanding of PBP binding spectra and target coverage could help to rationalize the clinical efficacy of combination therapies and inform optimization of antimicrobial regiments.

Accordingly, the objectives of this research are to: (i) define the PBP profiles of *G. adiacens* and identify PBP alterations, responsible for reduced beta-lactam susceptibility; (ii) evaluate the inhibitory activity of mono- versus dual beta-lactam regimens, comparing the current standard combination (ampicillin plus ceftriaxone) with the off-label use of ceftobiprole (BPR), a fifth generation cephalosporin with activity against low-affinity PBPs, such as *E. faecalis* PBP4; and (iii) correlate PBP acylation levels with mutations that may affect antibiotic binding.

## 2. Results

### 2.1. Phenotypic Characteristics of Selected Strains

Antibiotic susceptibility testing showed that the clinical strain of *G. adiacens* IS 48 was non-susceptible to penicillin (MIC 0.5 mg/L) and ampicillin (MIC 0.5 mg/L), resistant to ceftriaxone (MIC 32 mg/L) and cefotaxime (MIC 8 mg/L), and exhibited elevated MICs for ceftobiprole (MIC 0.19 mg/L) and ceftaroline (MIC 2 mg/L) compared with the reference ATCC strain, which was susceptible to all tested beta-lactams. Specifically, the MIC values of *G. adiacens* ATCC 49175 were: penicillin, 0.032 mg/L; ampicillin, 0.016 mg/L; ceftriaxone, 0.064 mg/L; cefotaxime, 0.5 mg/L; ceftobiprole, 0.023 mg/L; and ceftaroline, 0.047 mg/L. In addition, the combination of ampicillin with either ceftriaxone or ceftobiprole, each used at 1× MIC value, demonstrated synergistic activity [10].

### 2.2. Pbp Genes: Detection, Homology and Mutations

#### 2.2.1. In Silico Analysis, HMM-PBP Homology and Functional Annotation

16S rRNA gene sequencing (acc. N. FOQC00000000) indicated that *G. adiacens* is closely related to *Abiotrophia para-adiacens*, *Vagococcus coleopterurom*, *Carnobacterium maltaromaticum* and *Trichococcus flocculiformis*, as well as to certain enterococcal species, primarily *Enterococcus durans* and *Enterococcus faecium* (100% query coverage and about 93% sequence identity).

Preliminary in silico analysis of the *G. adiacens* ATCC 49175 genome (acc. N. CP102283.1) identified five genes encoding HMM PBPs (Table 1) showing distinct sequences with high similarity for the *C. maltaromaticum* and *T. flocculiformis* PBPs (acc. N. FOQC00000000). Owing to the lack of available functional data for PBPs from *T. flocculiformis* and *C. maltaromaticum*, the taxonomic placement of *Granulicatella* spp. within the NVS group, and the results of the 16S rRNA phylogenetic analysis, enterococcal and streptococcal PBP sequences were selected as references for sequence alignments.

Three HMM *pbp* genes encoded bifunctional enzymes, possessing both transglycosylase and transpeptidase activities: PBP1B (834 aa), PBP1A (827 aa), and PBP2A (720 aa). The remaining two genes encoded monofunctional transpeptidases comprising a penicillin-binding dimerization domain and a transpeptidase domain: PBP2B (711 aa) and PBP2 (595 aa) (Table 1).

The annotations obtained from the diverse online databases were concordant, with the sole exception of the PBP encoded by the *pbp* gene at locus NQ540_RS07070. UniProt and Swiss Model report this protein as being 711 amino acid in length, whereas the NCBI entry lacks the first four residues, yelding a predicted length of 707 amino acids and a correspondingly lower molecular mass. Aside from these four N-terminal residues, the aligned sequences showed 100% identity.

Further analyses enabled the prediction of the subcellular localization and domain organization of the PBPs and allowed the identification of the three conserved motifs within the transpeptidase (TP) domain: motif I (SxxK), motif II (SxN), and motif III (KTG) (Table 2). PBP1B, PBP1A and PBP2A were predicted to be membrane proteins exposed to the periplasmic space, whereas PBP2B and PBP2 were prediceted to be integral cytoplasmic membrane proteins. Consistent with their bifunctional nature, PBP1B, PBP1A and PBP2A harbored both glycosyltransferase and transpeptidase domains. In contrast, PBP2B and PBP2 contained a penicillin-binding protein dimerization domain and a TP domain, and were thus classified as monofunctional enzymes.

**Figure 1:**
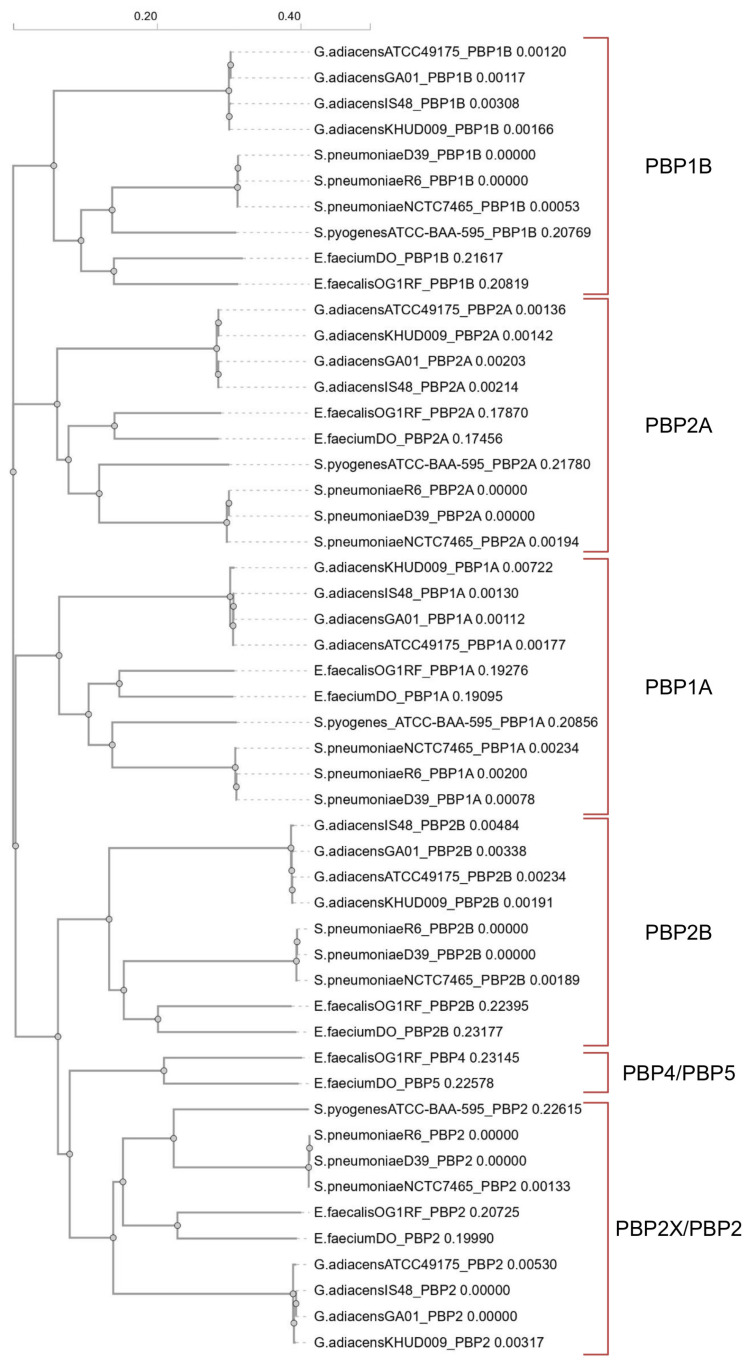
Phylogram generated from the multi-alignment of amino acid sequences using Clustal Omega v1.2.4 (https://www.ebi.ac.uk/jdispatcher/msa/clustalo, accessed on 6 September 2025). Each amino acid sequence is designated according to the following order: “abbreviated species_strain_PBP name”.

The phylogenetic tree (Figure 1) derived from the multiple amino acid sequence alignment revealed that *G. adiacens* PBPs shared homology with both streptococcal and enterococcal PBPs. Pairwise sequence identity values ranged from about 40–48% between *G. adiacens* and *E. faecium* or *E. faecalis*, higher than those observed with streptococcal PBPs, as detailed in the PIM matrix (Table S1). Therefore, we designated the *G. adiacens* PBPs as PBP1B, PBP1A, PBP2A, PBP2B and PBP2, consistent with the nomenclature reported in Table 1 and Table 2.

#### 2.2.2. *G. adiacens* PBP Architecture and Sequence Analysis

**Figure 2:**
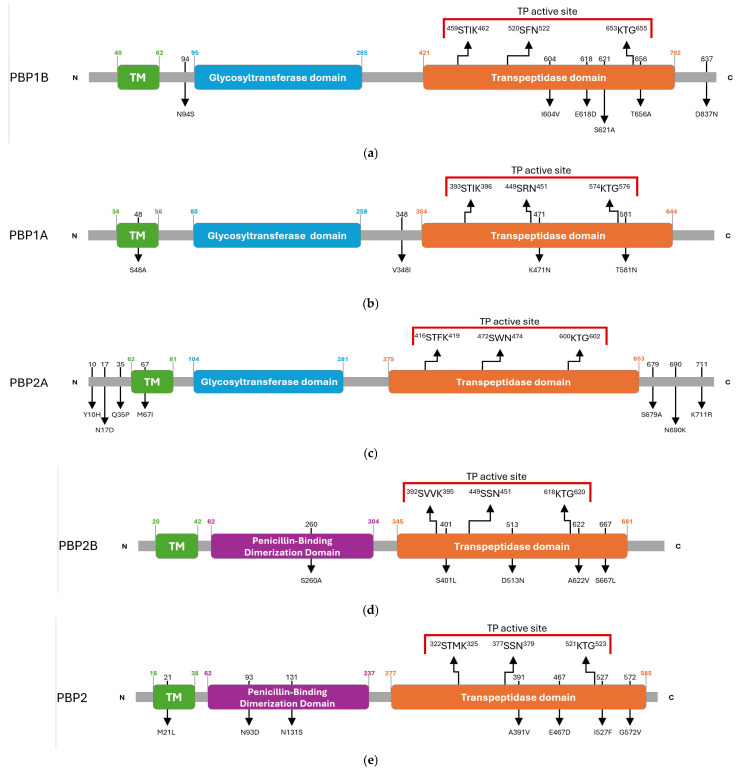
Schematic overview of amino acid substitutions and domain organization of *G. adiacens* HMM penicillin-binding proteins (PBPs): (**a**) PBP1B, (**b**) PBP1A, (**c**) PBP2A, (**d**) PBP2B, (**e**) PBP2. Arrows in the upper panel indicate the positions of the three conserved catalytic motifs. Red parentheses delineate the active site of the transpeptidase domain. Black numbers above the diagrams indicate the residue positions mutated in the *G. adiacens* IS 48 strain, and the corresponding amino acid substitutions are shown below. Green numbers indicate the start and end positions of the transmembrane (TM) helix. Blue numbers indicate the start and end positions of the glycosyl-transferase domain. Orange numbers indicate the start and end positions of the transpeptidase domain. Purple numbers indicate the start and end positions of the penicillin-binding dimerization domain.

The domain architecture of the HMM-predicted PBPs of *G. adiacens* was inferred by comparing the amino acid sequence of the clinical isolate IS 48 with that of the reference strain ATCC 49175. This comparative analysis identified multiple synonymous and missense substitutions distributed across transmembrane regions, catalytic domains, and the N- and C-terminal ends (Table S2). The overall PBP architectures of *G. adiacens*, with the amino acidic substitutions detected in the clinical isolate, are depicted in Figure 2.

Class A PBPs, which harbor both glycosyltransferase and transpeptidase (TP) domains, displayed specific substitutions in proximity to their catalytic centres.

In PBP1B, an N94S substitution was detected adjacent to the glycosyltransferase domain. Within the transpeptidase (TP) domain, three substitutions (I604V, E618D, and S621A) were identified between motifs II and III, and a T656A substitution was located adjacent to motif III. An additional D837N substitution was found in the C-terminal region (Figure 2a).

PBP1A carried a S48A substitution within the transmembrane (TM) helix and a V348I substitution near the N-terminus of the TP domain. Two further mutations were identified in the catalytic site, K471N and T581N, the latter occurring in the vicinity of motif III (Figure 2b).

PBP2A was the only PBP in which no substitutions were detected within the TP domain. Mutations were limited to the N-terminal region (Y10H, N17D, Q35P), TM region (M67I), and the C-terminal region (S679A, N690K, K711R) (Figure 2c).

Class B PBPs, which primarily act as transpeptidases, exhibited heterogeneous mutational profiles.

In PBP2B, substitutions included S260A in the dimerization domain and four mutations within the TP domain: S401L near motif I, D513N, A622V near motif III, and S667L (Figure 2d).

PBP2 carried multiple amino acid substitutions, comprising M21L in the TM region; N93D and N131S in the dimerization domain; and A391V, E467D, I527F and G572V in the TP domain, with I527F located in the proximity of motif III (Figure 2e).

### 2.3. HMM-PBP Binding Profiles and Beta-Lactam Affinity

**Figure 3:**
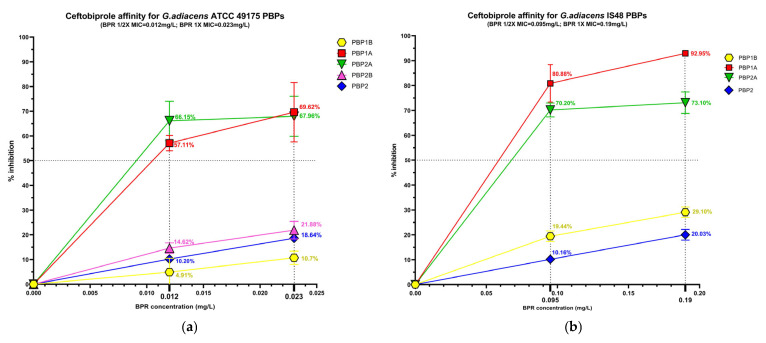

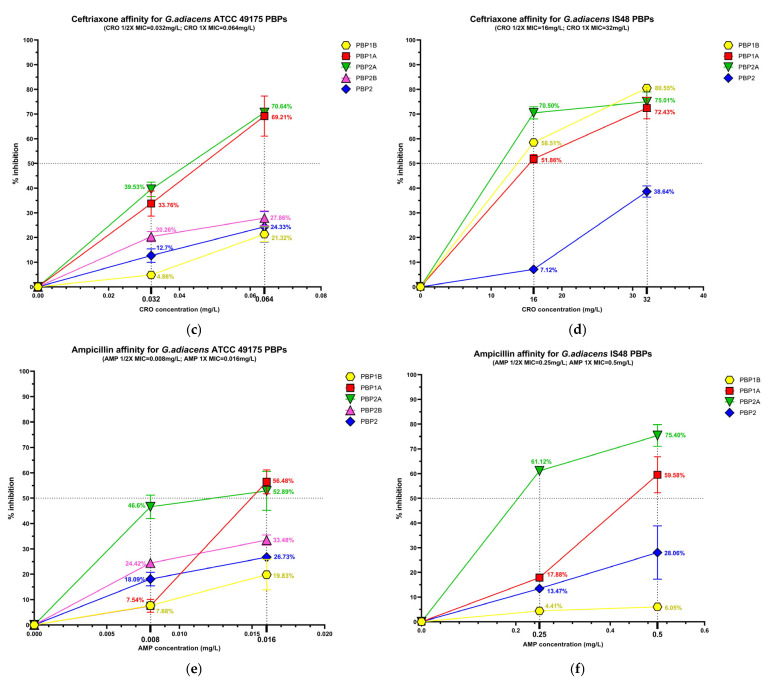
Relative binding affinities of PBPs for beta-lactams: (**a**) ceftobiprole affinity, (**c**) ceftriaxone affinity, (**e**) and ampicillin affinity for *G. adiacens* ATCC 49175 PBPs; (**b**) ceftobiprole affinity, (**d**) ceftriaxone affinity, (**f**) and ampicillin affinity for *G. adiacens* IS 48 PBPs. Yellow line: PBP1B; red line: PBP1A; green line: PBP2A; fuchsia line: PBP2B; blue line: PBP2. Error bars represent means of three independent biological replicates, each measured in triplicate assays, ±standard deviation (SD). Data were analyzed using GraphPad Prism 9 (version 9.0).

The beta-lactam interaction behaviour of *G. adiacens* with its preferential PBP targets was assessed by analysing the binding affinity of HMM-labeled PBPs for ampicillin (AMP), ceftobiprole (BPR) and ceftriaxone (CRO) (Figure S2). Affinity was quantify as the percentage of PBP under each experimental condition (Figure 3), followed by determination of the antibiotic concentration required to inhibit 50% of enzyme activity (IC_50_) for both the reference strain (ATCC 49175) and the clinical isolate (IS 48) (Table 3).

#### 2.3.1. PBP Affinity Under Single Beta-Lactam Exposure

Significant differences in acylation levels were observed across the PBP profiles of both the reference strain (ATCC 49175) and the clinical isolate (IS 48).

Among the PBPs, PBP1A and PBP2A exhibited the highest affinities for all beta-lactams tested. Ceftobiprole showed particularly pronounced activity in the clinical isolate (Figure 3a,b), where PBP1A acylation reached 80.88% at 1/2× MIC and 92.95% at 1× MIC. Ceftriaxone was active at 1/2× MIC against PBP1A and PBP2A exclusively in the clinical isolate, slightly exceeding 50% acylation (51.85% and 70.50%, respectively), and achieving higher acylation levels at 1× MIC in both isolates. Consistently, IC_50_ values for these targets were extremely low in the reference strain (0.01 mg/L for BPR and AMP) and moderately higher in the clinical isolate (Table 3).

PBP1B generally exhibited limited inhibition (< 30%) following exposure to BPR or AMP. In contrast, in the IS 48 clinical isolate it showed high affinity for ceftriaxone, with 80.55% acylation at 1× MIC (Figure 3d) and a corresponding IC_50_ of 15.12 mg/L (Table 3).

PBP2B and PBP2 displayed uniformly low acylation levels in all tested concentrations. In the reference strain, the inhibition of PBP2B did not exceed 33.48% even at 1× MIC of AMP, and PBP2B could not be analyzed in the clinical isolate due to an insufficient fluorescence signal. Similarly, PBP2 acylation remained below 40% in both strains for all treatment conditions (Figure 3). Owing to these low inhibition levels, IC_50_ values could not be reliably determined for these PBPs (Table 3).

#### 2.3.2. PBP Affinity Under Dual Beta-Lactam Exposure

**Figure 4:**
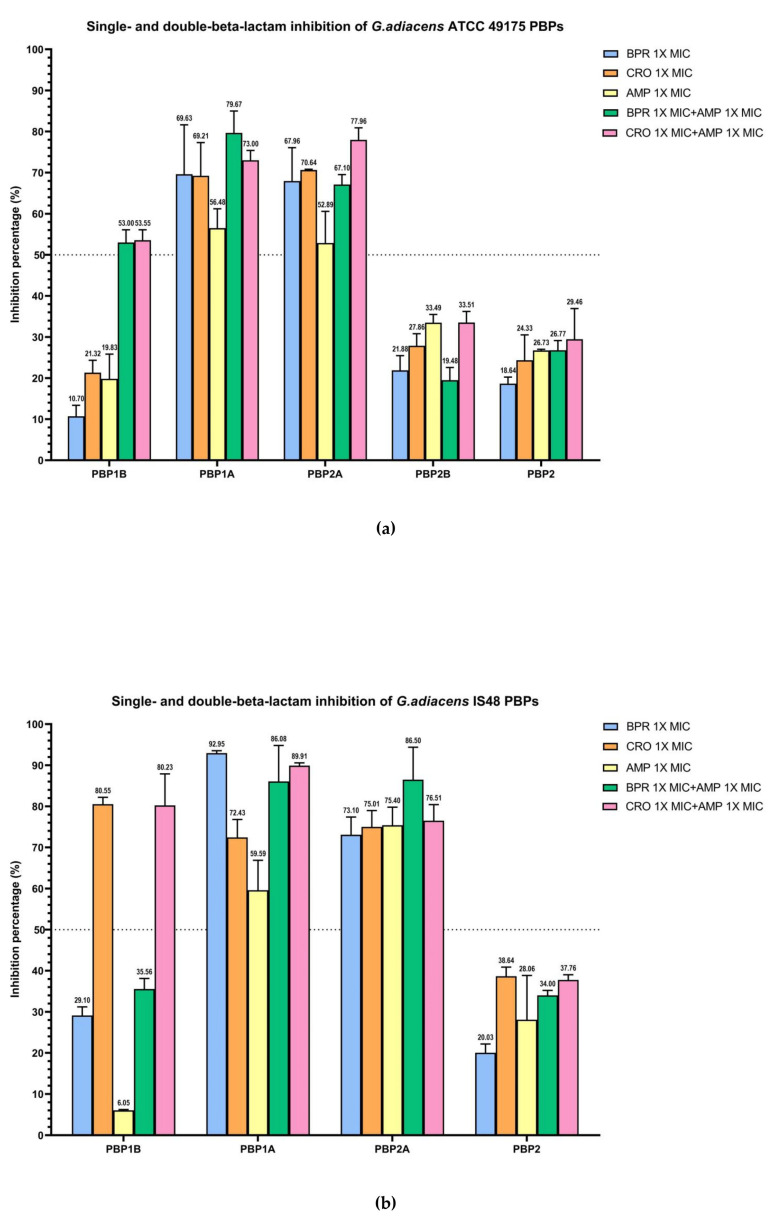
Relative binding affinities of beta-lactams, alone and in combination, for *G. adiacens* PBPs: (**a**) ATCC 49175 above; (**b**) IS 48. Data points and error bars indicate the mean of three independent biological replicates, each measured in triplicate, ± SD. Light blue: BPR 1× MIC; orange: CRO 1× MIC; yellow: AMP 1× MIC; green: BRP 1× MIC + AMP 1× MIC; pink: CRO 1× MIC + AMP 1× MIC. Graph generated using GraphPad Prism 9 (version 9.0).

The combination of ampicillin with ceftobiprole or ceftriaxone resulted in enhanced acylation of the three bifunctional HMM-PBPs compared with monotreatment (Figure 4).

In the ATCC 49175 reference strain, dual beta-lactam exposure produced synergistic inhibition of PBP1B with more than a two-fold increase in inhibition relative to each antibiotic alone (53% and 53.55% inhibition to ceftobiprole and ceftriaxone, respectively) yielding IC_50_ values of 0.02 mg/L for BPR + AMP and 0.04 mg/L for CRO + AMP (Figure 4a). In contrast, the IS 48 clinical isolate exhibited high inhibition with CRO + AMP (80.23%), which was comparable to ceftriaxone monotreatment, resulting in an IC_50_ of 14.26 mg/L (Figure 4b).

For PBP1A and PBP2A, the addition of ampicillin to either cephalosporin increased acylation, although the proportion of this effect differed between strains. In the ATCC 49175 reference strain, the IC_50_ for PBP1A with ceftriaxone plus ampicillin was reduced by half to 0.02 mg/L, with an inhibition rate of 73% (Figure 4a), whereas the IC_50_ for ceftobiprole plus ampicillin remained at 0.01 mg/L, with an inhibition of 79.67%. For PBP2A, ceftriaxone plus ampicillin decrease the IC_50_ to 0.02 mg/L representing a 33.3% reduction compared with ceftriaxone alone, and achieving 77.96% inhibition. The IC_50_ for PBP2A with ceftobiprole plus ampicillin also remained constant at 0.01 mg/L, with inhibition levels comparable to ceftriaxone alone (67.10% at 1× MIC).

In the IS 48 clinical strain, PBP1A inhibition with double-treatment with ceftriaxone plus ampicillin increased to 89.91% (IC_50_ 11.17 mg/L), compared to ceftriaxone monotreatment (72.43%—IC_50_ 15.77 mg/L), while the IC_50_ for ceftobiprole plus ampicillin remained unchanged at 0.06 mg/L (Table 3). For PBP2A, ceftriaxone plus ampicillin increased the inhibition rate to 76.51% (IC_50_ 4.83 mg/L). A more pronounced synergistic effect on PBP2A was observed with ceftobiprole plus ampicillin, for which inhibition increased from 73.10% to 86.50% (IC_50_ 0.07 mg/L), with respect to BPR monotreatment.

Overall, IC_50_ values were consistently higher in the *G. adiacens* IS 48 clinical isolate than in the ATCC 49175 reference strain, suggesting a potentially reduced target availability or affinity in the clinical setting (Table 3).

Statistical analysis of the IC_50_ values for all essential PBPs demonstrated a highly significant reduction in drug affinity in the IS 48 clinical isolate compared with the ATCC 49175 reference strain (*p* < 0.0001). In the resistant isolate, the addition of ampicillin to ceftriaxone (CRO + AMP) significantly improved binding affinity for the primary targets PBP1A and PBP2A compared with ceftriaxone alone (*p* < 0.001), providing a strong molecular basis for the observed clinical synergy. For PBP1B, the combination also produced a statistically significant, although less pronounced, reduction in IC_50_ (*p* < 0.05).

## 3. Discussion

Target alteration represents one of the most common mechanisms of beta-lactam resistance among Gram-positive bacteria. It reduces the affinity of PBPs for beta-lactams, thereby decreasing the stability of the beta-lactam/PBP complex. Particular attention is directed toward three conserved motifs—SXXK, SXN, and KTG—which constitute the catalytic extended cleft of the transpeptidase (TP) domain. Motif I (SXXK) harbours the catalytic serine residue that initiates the transpeptidation reaction through the formation of a covalent acyl-enzyme intermediate. Motif II (SXN) contributes to substrate stabilization and orientation via hydrogen bonding, whereas Motif III (KTG) is involved in substrate positioning and proton catalysis through the lysine residue [12].

Although 16S rRNA gene sequencing of *G. adiacens* revealed little similarity to *E. faecium* and *Streptococcus* spp., high sequence identity and coverage with *Carnobacterium maltaromaticum* and *Trichococcus flocculiformis* were observed. These Gram-positive, rod-shaped bacteria are facultative anaerobes and non-spore-forming environmental microorganisms. Like *G. adiacens*, they are classified within the phylum Firmicutes and the family *Carnobacteriaceae*. *C. maltaromaticum* is occasionally pathogenic in fish and can exhibit multidrug resistance, while data on antibiotic resistance of *T. flocculiformis* remain scarce [16,17]. Data on penicillin-binding proteins (PBPs) in either *T. flocculiformis* or *C. maltaromaticum* are currently lacking; consequently, we focused our comparative analysis on streptococci and enterococci, given the extensive functional characterization of the *S. pneumoniae* PBPs and the observed phylogenetic proximity with *E. faecium*. Despite *Granulicatella* being reclassified as a distinct genus, separate from *Streptococcus*, the percentage of PBP sequence identity between *G. adiacens* and streptococci remained in the range of approximately 35–42%.

In *S. pneumoniae*, PBP1A, PBP2B, and PBP2X are known to play a central role in beta-lactam resistance, particularly when mutations extend across the entire TP domain [18]; alterations confined to PBP2X and/or PBP2B confer low-level resistance, while additional mutations in PBP1A are required to achieve high-level resistance to both penicillins and cephalosporins [19].

Kocaoglu et al. [20] demonstrated that PBP2X and PBP3 bind the majority of beta-lactams, while PBP1A selectively interacts with ampicillin, cephalothin, and carbapenems. Ceftaroline and ceftriaxone exhibit high affinity for most PBPs, with the notable exception of PBP2B. In resistant pneumococcal strains, PBP1B and PBP2X display elevated IC_50_ values for ceftriaxone [21].

Analysis of the three catalytic motifs revealed that *G. adiacens* exhibits high sequence similarity to other species, as indicated by multiple sequence alignment (Figure S1), namely ST(M/I)K or SVVK for motif I, S(S/R/F/W)N for motif II, and KTG(S/T) for motif III.

Macheboeuf et al. [18] found that a threonine residue at position +1 relative to the KTG motif interacts with the N-terminus of a downstream β-sheet playing a key role in stabilizing the closed conformation of the enzyme. In the corresponding position, *G. adiacens* IS 48 PBP1B harbours a T656A substitution, which account for the marked reduction in ampicillin acylation rates. The T656A substitution is immediately adjacent to motif III (^653^KTG^655^), in proximity to the catalytic cleft; alterations in the structure and polarity of this amino acid may disrupt local interactions within the cleft, thereby decreasing affinity for beta-lactams, as observed for ampicillin but not for the two cephalosporins.

This mechanistic explanation is consistent with the apparent efficacy of dual beta-lactam treatment in the *G. adiacens* ATCC 49175 reference strain, in which both drug combinations enhanced the degree of inhibition from 10.70 to 21.32% with single agents to as much as 53.00–53.55%. In contrast, this potentiation was not observed in the clinical strain, where the inhibition achieved with the ampicillin–cephalosporin combination was comparable to that obtained with cephalosporin alone.

Exposure of *G. adiacens* PBP1A to the combination of ceftriaxone plus ampicillin resulted in a 50% reduction in target activity in the reference strain, whereas the effect was slightly attenuated in the clinical isolate (29.20%). As for PBP1B, this diminished response in IS 48 may be associated with the T581N substitution, located near the catalytic motif III, which may represent a resistance-associated mutation. The most significant shift was observed for ceftriaxone, for which the IC_50_ increased to 15.77 mg/L in the clinical strain, indicating a substantial loss of potency compared to the reference strain.

Conversely, the combination of ceftobiprole and ampicillin did not further enhance inhibition compared with ceftobiprole alone; ceftobiprole consistently exhibited the strongest overall inhibitory activity. The ability of ceftobiprole to achieve 92.95% acylation of PBP1A at 1× MIC in the clinical isolate—despite the presence of the T581N mutation in the active-site—highlights its robust binding kinetics. These findings suggest that the side-chain architecture of ceftobiprole allows for a more stable interaction within the transpeptidase pocket of *G. adiacens* PBPs than that provided by conventional cephalosporins.

Although all IC_50_ values were below the corresponding MICs, the non-susceptible clinical isolate consistently exhibited higher IC_50_ values than the susceptible reference strain for all drugs tested. A similar tendency has been reported in studies comparing ceftriaxone-susceptible and -resistant *S. pneumoniae* isolates [22].

PBP2A showed a comparable trend in both *G. adiacens* strains: ceftobiprole in combination with ampicillin did not augment the activity observed with ceftobiprole monotreatment, resulting in an unchanged IC_50_, which remained at the lower concentration. In contrast, the ceftriaxone plus ampicillin combination produced a clear potentiation of inhibitory activity, with at least 33.3% reduction in IC_50_. Remarkably, PBP2A in the IS 48 clinical isolate did not harbor mutations within the transpeptidase domain yet still showed a reduced affinity for ceftriaxone. This observation suggests that non-catalytic substitutions, such as M67I in the transmembrane region and various C-terminal changes, may indirectly affect protein stability or modulate the accessibility of the active site to larger cephalosporin molecules.

In the study by Kosowska K et al. [21], penicillin-resistant *S. pneumoniae* strains with 2 to 4 mg/L MICs, exhibited ceftriaxone IC_50_ values for PBP2A ranging from 0.25 mg/L—similar to susceptible strains—up to 1 mg/L. In addition, Davies et al. (2007) [22] reported high affinity of ceftriaxone and ceftobiprole for PBP2A in both susceptible and resistant pneumococcal isolates.

In *S. pneumoniae,* resistance is frequently associated with substitution of conserved threonine in Motif I, resulting in the S(A/S)MK sequence, often in combination with a replacement of proline downstream of Motif II, as well as additional mutations in PBP2B and PBP2X [21]. By contrast, *G. adiacens* and *E. faecalis* possess two threonine residues following motif II (SRNTT), one of which corresponds to proline position in streptococci; these residues are conserved and have not been implicated in resistance. Although multiple substitutions were identified in IS 48 PBP2A, none localized near the catalytic motifs, suggesting that they are unlikely to contribute directly to beta-lactam resistance, consistent with the similar pharmacodynamic trend observed in the two strains.

Based on the lowest IC_50_ values measured, ampicillin, ceftobiprole, and ceftriaxone can be considered as selective for the bifunctional PBP1A and PBP2A in both strains, and for PBP1B (limited to ceftriaxone in the IS 48 clinical isolate); in contrast, binding affinity for monofunctional transpeptidases was weak. As previously described in *S. pneumoniae,* the monofunctional PBP2B and PBP2X are low-affinity targets for beta-lactams. Similarly, the PBP2B and PBP2 transpeptidases in *G. adiacens* clinical isolate also exhibited low-affinity for beta-lactams under both mono- and double-treatment, with inhibition levels remaining below 40% in most experiments. Even in the *G. adiacens* ATCC 49175 reference strain, these PBPs underwent only limited acylation and did not reach IC_50_ values, consistent with intrinsically low beta-lactam binding. For both transpeptidases, although IC_50_ values could not be determined, the acylation percentages indicated that the dual beta-lactam exposure did not enhance inhibition, relative to the corresponding mono-treatments.

**Figure 5:**
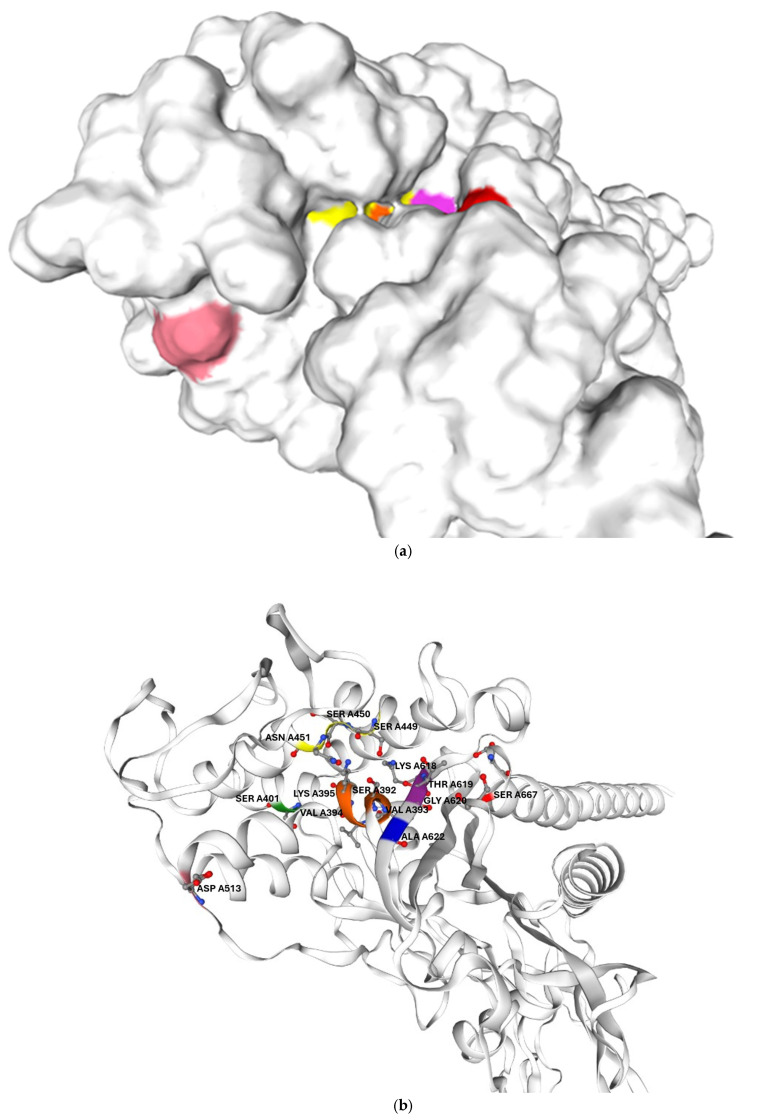

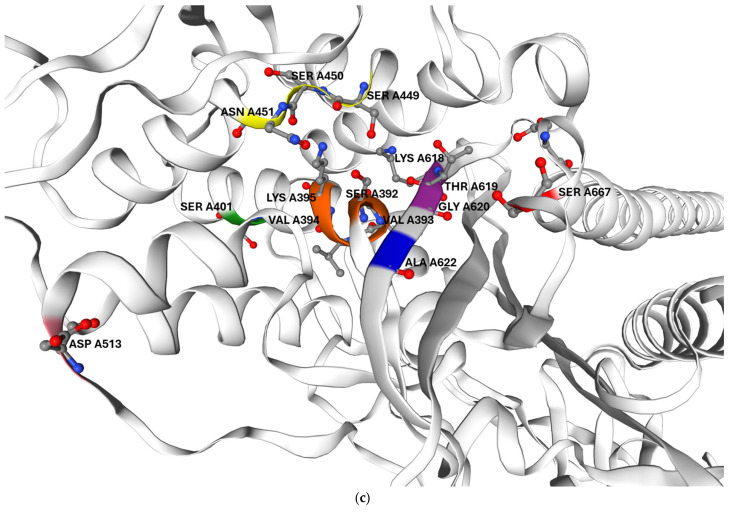
Predicted three-dimensional structure of *G. adiacens* ATCC 49175 PBP2B: (**a**) surface representation and (**b**) cartoon representation in the same orientation; (**c**) enlarged view of the cartoon representation highlighting the region proximal to the active site. The model was generated using Swiss-Model (https://swissmodel.expasy.org/, accessed on 6 September 2025). Orange: motif I; yellow: motif II; purple: motif III. Amino acid substitutions identified in *G. adiacens* IS 48 are displayed in distinct colors to emphasize their spatial proximity to the catalytic motifs: Asp 513 (D513N) in pink, Ala 622 (A622V) in blue, and Ser 667 (S667L) in red.

In addition, the mutations identified in the clinical isolate are likely to further reduced their drug affinity. Its PBP2B harbours two amino acid substitutions located in proximity to catalytic motifs I and III, which may reduce its affinity even for Bocillin-FL, as previously reported for other PBPs. This could account for the failure to detect PBP2B in *G. adiacens* IS 48 by Bocillin-FL labelling. In *G. adiacens* PBP2B, the polar serine residue at position 667, which lies on the protein surface near the beta-lactam binding site, is replaced in the clinical isolate by a nonpolar leucine residue, as shown in Figure 5. Prior studies have shown that amino acid substitutions altering the surface change in exposed regions adjacent to the beta-lactam binding pocket—though not directly involving the catalytic motif—can significantly impact antibiotic binding [23].

The clinical relevance of the synergy observed in the 1× MIC condition is supported by the comparison of our data with the pharmacokinetic (PK) parameters of the high-dose regimen successfully used to treat the patient from whom the IS 48 clinical strain was isolated [10]. In that case, continuous administration of 16 g/day ampicillin (4 g intravenously every 6 h) typically yields a free steady-state concentration (*fC_ss_*) of approximately 32–48 mg/L, whereas ceftriaxone (2 g intravenously every 12 h) achieves a free *Cmax* of ~25 mg/L. Our results demonstrate that the combination of these two beta-lactams reduces the IC_50_ values for the multidrug-resistant IS 48 clinical isolate to 4.83 mg/L for PBP2A and 11.17 mg/L for PBP1A (Table 3).

The statistically significant increase in PBP binding affinity observed with dual beta-lactam treatment provides a mechanistic basis for the in vivo synergy, underscoring that sufficient target occupancy is essential to circumvent sequence-mediated resistance in *G. adiacens*. Given that the free drugs concentrations attained in the patient markedly exceed these inhibitory thresholds, saturation of essential PBPs is readily achievable in vivo. This alignment between pharmacodynamic target achievement and clinical outcome offers a mechanistic explanation for the therapeutic success previously documented in this case of *G. adiacens* infective endocarditis.

## 4. Materials and Methods

### 4.1. Bacterial Strains

To characterize the PBP profile of *G. adiacens*, we used the ATCC 49175 reference strain and a clinical isolate (IS 48) obtained from a documented case of infective endocarditis successfully treated with double beta-lactam therapy, as described by Pagotto A. et al. [10].

### 4.2. In Silico Analysis

*In silico* analyses were performed on the *G. adiacens* ATCC 49175 genome (acc. N. CP102283.1) strain retrieved from the NCBI database to: (i) investigate the phylogenetic position of this species using 16S rRNA gene sequence analysis; and (ii) identify and localize genes encoding High Molecular Mass PBPs (>45kDa). The theoretical molecular weight of each PBP was calculated from the amino acid and nucleotide sequences deposited in GenBank (https://www.ncbi.nlm.nih.gov/nuccore/CP102283.1/, accessed on 6 September 2025) using the online bioinformatic resource “Quest CalculateTM Peptide and Protein Molecular Weight Calculator” (AAT Bioquest, Inc., Pleasanton, CA, USA) (https://www.aatbio.com/tools/calculate-peptide-and-protein-molecular-weight-mw, accessed on 6 September 2025). Additional functional and structural analyses were performed using UniProt (https://www.uniprot.org/, accessed on 6 September 2025) and SwissModel (https://swissmodel.expasy.org/, accessed on 6 September 2025).

To assess PBP homology within *G. adiacens*, PBP were aligned by BLASTp (NCBI BLAST+ 2.17.0) against the core nucleotide databases corresponding to *G. adiacens* ATCC 49175 (acc. N. CP102283.1), other deposited *G. adiacens* genomes (*G. adiacens* strain GA01, acc. N. CP198988.1; *G. adiacens* strain KHUD_009, acc. N. CP106751.1), and the genome of the *G. adiacens* clinical isolate IS 48 (acc. N. PRJNA1358263/).

Transpeptidase motifs were identified and localized through multiple sequence alignments including *Streptococcus pneumoniae* and additional closely related species (according to the former taxonomic classification of *G. adiacens*). The genomes used for alignment were: *S. pneumoniae* R6 (acc. N. NC_003098.1), *S. pneumoniae* NCTC (acc. N. LN831051.1), *S. pneumoniae* D39 (acc. N. CP000410.2), *S. pyogenes* (acc. N. NC_004070.1), *Enterococcus faecium* ATCC BAA-472/TX0016/DO (acc. N. CP003583.1), *E. faecalis* ATCC 47077 OG1RF (acc. N. CP025020.1). Multiple sequence alignments were performed using Clustal Omega v1.2.4 (EMBL-EBI Job Dispatcher sequence analysis tools framework, 2024) “https://www.ebi.ac.uk/jdispatcher/msa/clustalo” (accessed on 6 September 2025), and SnapGene v8.2.2. (www.snapgene.com, accessed on 6 September 2025).

### 4.3. PBP-Binding Assays Analysis

PBP-binding assays were carried out to evaluate the affinity of PBPs for beta-lactam antibiotics, both alone and in combination, by calculating IC_50_ values, defined as the antibiotic concentration that inhibits 50% of the enzyme activity, inferred as the degree of PBP acylation.

Assays, we performed following the protocol previously described by Conti P., Lazzaro L.M. et al. [24], with modifications to optimize growth conditions of *Granulicatella* spp.

Fresh cultures in chocolate agar plates supplemented with L-cysteine (0.001 mg/mL) were used to prepare a starting inoculum of 10^5^–10^6^ CFU/mL (OD_620_ = 0.06) in brain heart infusion (BHI) broth (OXOID) supplemented with 5% lysed horse blood 50% (LHB_50%_) and L-cysteine (Sigma-Aldrich, Milan, Italy). After incubation for 4 h at 37 °C, cultures reached OD_620_ = 0.2, after which cells were harvested by centrifugation (8000 rpm for 5 min at room temperature) and washed with Phosphate-Buffer Saline (PBS) (10 mM, pH 7.4) (OXOID). Cell suspensions were then incubated for 30 min at 37 °C under the following conditions:

− BPR 1/2× MIC and BPR 1× MIC
− CRO 1/2× MIC and CRO 1× MIC
− AMP 1/2× MIC and AMP 1× MIC
− BPR 1/2× MIC + AMP 1× MIC and BPR 1/2× MIC + AMP 1× MIC
− CRO 1/2× MIC + AMP 1× MIC and CRO 1× MIC + AMP 1× MIC

After treatment, cells were resuspended in PBS and exposed to Bocillin^FL^ (7.6 µM) for 10 min at 37 °C. Cells were then lysed with lysozyme (10,000 mg/L, 30 min at 37 °C) followed by sonication using a Bandelin Sonifier (Bandelin electronic GmbH & Co. KG, Berlin, Germany) (30 s, 30% duty cycle, 5 intervals).

Proteins were resuspended in PBS, and their concentration was measured using the Qubit Protein Assay Kit and Qubit 3.0 (Invitrogen-life Technologies, Turin, Italy) then normalized across samples. Prior to electrophoresis, protein samples were mixed with 4 × Laemmli buffer and beta-mercaptoethanol and denatured by heating (5 min, 90 °C). Samples were resolved on Criterion^TM^ TGX^TM^ (Tris-Glycine extended) 8–16% polyacrylamide gels at 100 V for 3 h. Fluorescently labelled PBPs were visualized with a Typhoon FLA 9500 scanner (Alexa fluor 488 filter, PMT 1000, pixel 50 µm; Washington, DC, USA). Total protein content was evaluated by Coomassie Brilliant Blue R-250 staining (40% methanol, 10% glacial acetic acid, 0.1% Coomassie Brilliant Blue, R-250; S. Louis, MO, USA), followed by destaining in distilled water.

Densitometric analysis of PBP acylation was carried out using ImageJ v1.54p (https://imagej.net/, accessed on 25 September 2025) [25]. PBPs labelled with Bocillin^FL^ in the absence of antibiotics were defined as 100% Bocillin^FL^ binding. IC5_0_ values were estimated by log-linear interpolation of the binding percentages at antibiotic concentrations bracketing the MIC. Calculations were performed using AAT Bioquest IC_50_ Calculator (https://www.aatbio.com/tools/ic50-calculator, accessed on 25 September 2025) applying a four-parameter logistic regression model, and confirmed using GraphPad Prism 9.0 software (GraphPad, San Diego, CA, USA).

### 4.4. DNA Extraction and Whole-Genome Sequencing

Genomic DNA was extracted using the QIAamp DNA Mini Kit (QIAGEN, Hilden, Germany) following the manufacturer’s instructions. DNA concentration was quantified using the Qubit dsDNA HS Assay Kit (Invitrogen, Carlsbad, CA, USA) and a Qubit 3.0 fluorimeter (Invitrogen-life Technologies, Turin, Italy).

Whole-genome sequencing was performed on the Illumina MiSeq platform (Illumina, San Diego, CA, USA). Library preparation and samples normalization were carried out following Eurofins standard procedures, and sequencing was outsourced to Eurofins Genomics (Ebersberg, Germany). Genome assembly and annotations were performed using the BacExplorer v1.0 tool, a locally installable, user-friendly application, that generates an interactive HTML report (available at https://github.com/gretep/BacExplorer, accessed on 25 September 2025) [26].

### 4.5. Statistical Analysis

Quantitative data are presented as mean ± standard deviation (SD) derived from three independent biological replicates (*n* = 3). Comparisons between the reference strain and the clinical isolate were performed using an unpaired Student’s *t*-test. The effect of antibiotic combinations versus single agents was evaluated by one-way analysis of variance (ANOVA), followed by Tukey’s post-hoc test for multiple comparisons. A *p*-value < 0.05 was considered statistically significant. All statistical analyses were conducted using GraphPad Prism 9.0 software (GraphPad, San Diego, CA, USA).

## 5. Conclusions

Our results demonstrate a comparatively higher affinity of class A HMM PBPs for beta-lactams in *G. adiacens*. Among the drugs tested, cephalosporins emerged as the most potent agents, exhibiting sustained and preferential reactivity toward PBP1A and PBP2A, irrespective of the mutational profiles of the IS 48 clinical strain. In the IS 48 clinical isolate, ceftriaxone also exhibited a superior binding profile for PBP1B. The absence of detectable acylation observed for PBP2B and negligible for PBP2 is likely attributable to their structural and functional similarity to the low-affinity PBP2B and PBP2X of *S. pneumoniae*, which are well-established contributors to beta-lactam resistance.

In addition, our data provide a robust rationale for the enhanced efficacy of dual beta-lactam combinations against *G. adiacens*. This synergistic interaction is plausibly mediated by near-complete PBP saturation, whereby ampicillin may engage specific low-affinity targets or induce conformational rearrangements that facilitate the binding of a second antibiotic. Such “target-level synergy” may be particularly relevant for the treatment of “fastidious” species, in which monotherapy frequently fails to achieve the ≥50% PBP inhibition threshold associated with bactericidal activity.

Although further investigations are required to evaluate *pbp* gene expression levels, their impact on architecture and dynamics of the *Granulicatella* cell wall, and additional non-PBP factors that may modulate resistance to beta-lactam, the present study highlights a promising therapeutic approach for the management of *G. adiacens* infective endocarditis, and supports continued research aimed at optimizing clinical outcomes.

## Figures and Tables

**Table 1:** In silico analysis of *pbp* genes from *G. adiacens* ATCC 49175 (accession no. CP102283.1) performed using NCBI, UniProt, Swiss-Model, and AAT BioQuest.

PBP	Gene	GenBank CP102283.1 (Position)	Locus Tag	N. Nucleotides (bp)	Product (NCBI)	Protein_id (NCBI)	Aminoacids (aa) (NCBI)	UniProt Code	Molecular Weight (kDa) (AAT BioQuest)
PBP1B	-	1406082–1408613	NQR540_06930	2532	Transglycosylase domain-containing protein	WP_005606738.1	834	C8NFU9_9LACT	94.399
PBP1A	*pbp*1A	1064311–1066794	NQ540_RS05265	2484	Transglycosylase domain-containing protein	WP_005605597.1	827	C8NEX4_9LACT	91.762
PBP2A	*pbp*2A	635391–637553	NQ540_RS03315	2163	Transglycosylase domain-containing protein	WP_005604983.1	720	C8NDT8_9LACT	80.610
PBP2B	*pbp*	1435119–1437242	NQ540_RS07070	2124	Penicillin-binding protein 2	WP_156780452.1	707	C8NFX8_9LACT	77.964
PBP2	*pbp*C	1296607–1298394	NQ540_RS06305	1788	Penicillin-binding protein 2	WP_005605984.1	595	C8NFI6_9LACT	66.034

**Table 2:** Characteristics, predicted subcellular localization, domain architecture, and transpeptidase motifs of *G. adiacens* penicillin-binding proteins (PBPs) as determined using SMART (Simple Modular Architecture Research Tool; “https://smart.embl.de/ (accessed on 6 September 2025)” indicates absence of the respective domain.

*G. adiacens* PBPs	Feature			Domains					
	Subcellular Location	Type	Position	Penicillin-Binding Protein Dimerization	Glycosyl Transferase	Transpeptidase			
				Position	Position	Position	Motif I	Motif II	Motif III
PBP1B	membrane; outer membrane-bounded periplasmic space	Transmembrane	40–62	−	95–285	421–702	^459^STIK^462^	^520^SFN^522^	^653^KTG^655^
PBP1A	membrane; outer membrane-bounded periplasmic space	Transmembrane	34–56	−	80–259	354–644	^393^STIK^396^	^449^SRN^451^	^574^KTG^576^
PBP2A	membrane; outer membrane-bounded periplasmic space	Transmembrane	62–81	−	104–281	375–653	^416^STFK^419^	^472^SWN^474^	^600^KTG^602^
PBP2B	cell membrane	Transmembrane	20–42	62–304	−	345–681	^392^SVVK^395^	^449^SSN^451^	^618^KTG^620^
PBP2	cell membrane	Transmembrane	16–38	62–237	−	277–585	^322^STMK^325^	^377^SSN^379^	^521^KTG^523^

**Table 3:** IC_50_ values (mg/L) for the PBPs of *G. adiacens* strains.

PBP	Strain	IC_50_ (mg/L)				
		AMP	BPR	BPR + AMP	CRO	CRO + AMP
PBP1B	ATCC 49175	N.A.	N.A.	0.02 ± 0.00	N.A.	0.04 ± 0.00
	IS 48	N.A.	N.A.	N.A.	15.12 ± 0.22	14.26 ± 0.09 *
PBP1A	ATCC 49175	0.01 ± 0.00	0.01 ± 0.00	0.01 ± 0.00	0.04 ± 0.00	0.02 ± 0.00
	IS 48	0.31 ± 0.02	0.07 ± 0.01	0.06 ± 0.00	15.77 ± 0.19	11.17 ± 0.13 ***
PBP2A	ATCC 49175	0.01 ± 0.00	0.01 ± 0.00	0.01 ± 0.00	0.03 ± 0.00	0.02 ± 0.00
	IS 48	0.23 ± 0.01	0.06 ± 0.01	0.07 ± 0.01	11.12 ± 0.32	4.83 ± 0.17 ***
PBP2B	ATCC 49175	N.A.	N.A.	N.A.	N.A.	N.A.
	IS 48	N.A.	N.A.	N.A.	N.A.	N.A.
PBP2	ATCC 49175	N.A.	N.A.	N.A.	N.A.	N.A.
	IS 48	N.A.	N.A.	N.A.	N.A.	N.A.

Values are expressed as mean ± standard deviation (SD) from three independent biological replicates (*n* = 3). AMP, ampicillin; BPR, ceftobiprole; CRO, ceftriaxone. N.A., not applicable (binding not detectable). Statistical significance for the CRO + AMP combination relative to CRO alone is denoted as follows: *** *p* < 0.001 (highly significant); * *p* < 0.05 (significant). All IC_50_ values for the clinical isolate IS 48 were significantly higher than those obtained for the reference strain ATCC 49175 (*p* < 0.0001).

## Data Availability

The original contributions presented in the study are publicly available. This data can be found at: https://www.ncbi.nlm.nih.gov/bioproject/PRJNA1358263/ (31 January 2026).

## Supplementary Materials

The following supporting information can be downloaded at: https://www.mdpi.com/article/10.3390/antibiotics15020168/s1. Figure S1, multiple amino acid sequence alignment of PBP generated by Clustal Omega; Figure S2, Representative original raw images of fluorescence scans and SDS-PAGE gels depicting Bocillin^FL^-labelled PBP profiles; Table S1, PIM matrix; Table S2, *pbp* gene mutations and corresponding amino acid substitutions in the *G. adiacens* clinical isolate IS 48.
