# Supplementary material for: Target Fidelity and Failure: Structure–Activity Relationship of High-Molecular-Mass Penicillin-Binding Proteins (HMM-PBPs) in Refractory Granulicatella adiacens Endocarditis
Source: Antibiotics (Basel). 2026 Feb 5;15(2):168. doi: 10.3390/antibiotics15020168 (PMC12937247; doi:10.3390/antibiotics15020168)
Supplement: Supplementary file 1 [file antibiotics-15-00168-s001.zip › Supplementary files/Table S1. PIM matrix 1m.pim.pdf]

```
#
#
#  Percent Identity  Matrix - created by Clustal2.1
#
#

     1: G.adiacensATCC49175_PBP1B      100.00   99.76   99.17   99.41   36.15   36.15   36.15   38.56   40.47   41.62   30.66
30.35   30.20   30.20   29.17   28.19   27.15   26.30   26.30   26.45   26.69   26.69   26.84   26.69   26.45   24.39   24.81
25.00   24.85   25.00   15.85   15.85   15.85   15.85   15.54   15.54   15.54   18.28   16.94   15.51   14.31   13.69   14.74
14.74   14.74   16.50   16.50   16.50   16.50   14.93   17.32
     2: G.adiacensGA01_PBP1B            99.76  100.00   99.17   99.41   36.27   36.27   36.27   38.70   40.34   41.62   30.66
30.35   30.20   30.20   29.17   28.19   27.15   26.30   26.30   26.45   26.69   26.69   26.84   26.69   26.45   24.39   24.81
25.00   24.85   25.00   15.85   15.85   15.85   15.85   15.54   15.54   15.54   18.28   16.94   15.51   14.31   13.69   14.74
14.74   14.74   16.50   16.50   16.50   16.50   14.93   17.32
     3: G.adiacensIS48_PBP1B            99.17   99.17  100.00   99.53   36.40   36.40   36.40   38.83   40.47   41.88   30.66
30.35   30.20   30.20   29.02   28.34   27.30   26.45   26.45   26.61   27.12   27.12   27.26   27.12   26.45   24.25   24.81
24.85   24.69   24.85   15.66   15.66   15.66   15.66   15.54   15.54   15.54   17.92   16.76   15.51   13.92   13.69   14.74
14.74   14.74   16.50   16.50   16.50   16.50   14.93   17.16
     4: G.adiacensKHUD009_PBP1B         99.41   99.41   99.53  100.00   36.52   36.52   36.52   38.96   40.60   41.88   30.82
30.51   30.35   30.35   29.17   28.49   27.45   26.61   26.61   26.76   27.12   27.12   27.26   27.12   26.74   24.53   24.81
25.00   24.85   25.00   15.85   15.85   15.85   15.85   15.72   15.72   15.72   18.10   16.94   15.71   14.12   13.85   14.90
14.90   14.90   16.70   16.70   16.70   16.70   15.09   17.32
     5: S.pneumoniaeD39_PBP1B           36.15   36.27   36.40   36.52  100.00  100.00   99.88   58.17   45.60   47.78   26.26
26.26   26.10   26.10   27.96   26.65   27.38   26.32   26.32   26.48   25.33   25.18   25.18   25.18   26.39   25.84   24.49
25.04   25.04   25.20   15.44   15.62   15.62   15.81   18.01   18.01   18.18   18.43   14.75   15.66   16.05   12.68   13.06
13.06   13.06   13.59   13.59   13.59   13.59   12.80   15.51
     6: S.pneumoniaeR6_PBP1B            36.15   36.27   36.40   36.52  100.00  100.00   99.88   58.17   45.60   47.78   26.26
26.26   26.10   26.10   27.96   26.65   27.38   26.32   26.32   26.48   25.33   25.18   25.18   25.18   26.39   25.84   24.49
25.04   25.04   25.20   15.44   15.62   15.62   15.81   18.01   18.01   18.18   18.43   14.75   15.66   16.05   12.68   13.06
13.06   13.06   13.59   13.59   13.59   13.59   12.80   15.51
     7: S.pneumoniaeNCTC7465_PBP1B      36.15   36.27   36.40   36.52   99.88   99.88  100.00   58.17   45.60   47.78   26.26
26.26   26.10   26.10   27.81   26.65   27.38   26.48   26.48   26.64   25.33   25.18   25.18   25.18   26.54   25.99   24.49
25.04   25.04   25.20   15.44   15.62   15.62   15.81   18.01   18.01   18.18   18.43   14.75   15.66   16.05   12.68   13.06
13.06   13.06   13.59   13.59   13.59   13.59   12.80   15.51
     8: S.pyogenesATCC-BAA-595_PBP1B    38.56   38.70   38.83   38.96   58.17   58.17   58.17  100.00   45.15   50.13   28.25
28.25   28.25   28.25   27.34   26.57   25.23   26.37   26.37   26.52   25.93   25.77   25.77   25.77   26.03   24.53   24.72
24.47   24.15   24.31   14.44   14.63   14.63   14.81   17.07   17.07   16.88   18.92   16.51   18.11   15.69   14.72   13.26
13.26   13.26   15.35   15.35   15.35   15.35   12.10   14.90
     9: E.faeciumDO_PBP1B               40.47   40.34   40.47   40.60   45.60   45.60   45.60   45.15  100.00   57.56   26.35
26.19   26.35   26.35   26.53   26.58   25.54   26.75   26.75   26.59   25.45   25.45   25.45   25.45   25.00   24.32   23.62
23.38   23.22   23.38   14.50   14.50   14.50   14.68   14.15   14.15   14.21   16.27   14.78   14.17   13.37   13.34   11.92
11.92   11.92   13.73   13.73   13.73   13.73   13.14   14.41
    10: E.faecalisOG1RF_PBP1B           41.62   41.62   41.88   41.88   47.78   47.78   47.78   50.13   57.56  100.00   26.34
26.34   26.34   26.34   26.93   26.37   27.06   26.82   26.82   26.98   24.96   24.96   24.96   24.96   27.15   25.98   24.76
```

23.27   23.11   23.27   13.79   13.79   13.79   13.97   14.36   14.36   14.50   16.09   15.54   15.46   14.29   12.86   12.44
12.44   12.44   13.93   13.93   13.93   13.93   13.31   14.55
11: G.adiacensATCC49175_PBP2A   30.66   30.66   30.66   30.82   26.26   26.26   26.26   28.25   26.35   26.34   100.00
99.72   99.17   99.03   46.33   49.93   40.79   42.25   42.25   42.40   28.23   28.08   28.08   28.08   28.07   28.55   30.87
29.34   29.18   29.34   18.65   18.85   18.85   19.04   17.50   17.50   17.31   18.62   16.44   16.97   16.77   14.77   14.31
14.31   14.31   18.40   18.61   18.61   18.61   15.12   18.15
12: G.adiacensKHUD009_PBP2A   30.35   30.35   30.35   30.51   26.26   26.26   26.26   28.25   26.19   26.34   99.72
100.00   99.17   99.03   46.33   49.93   40.93   42.25   42.25   42.40   28.23   28.08   28.08   28.08   28.07   28.55   30.87
29.34   29.18   29.34   18.65   18.85   18.85   19.04   17.50   17.50   17.31   18.62   16.44   16.97   16.77   14.77   14.31
14.31   14.31   18.40   18.61   18.61   18.61   15.12   18.33
13: G.adiacensGA01_PBP2A   30.20   30.20   30.20   30.35   26.10   26.10   26.10   28.25   26.35   26.34   99.17
99.17   100.00   99.58   46.33   49.78   40.79   42.25   42.25   42.40   27.93   27.79   27.79   27.79   27.77   28.40   31.02
29.50   29.34   29.50   18.65   18.85   18.85   19.04   17.50   17.50   17.31   18.62   16.44   16.97   16.77   14.77   14.31
14.31   14.31   18.40   18.61   18.61   18.61   15.12   18.33
14: G.adiacensIS48_PBP2A   30.20   30.20   30.20   30.35   26.10   26.10   26.10   28.25   26.35   26.34   99.03
99.03   99.58   100.00   46.19   49.63   40.79   42.25   42.25   42.40   28.23   28.08   28.08   28.08   27.77   28.40   30.87
29.50   29.34   29.50   18.65   18.85   18.85   19.04   17.50   17.50   17.31   18.62   16.44   16.97   16.77   14.77   14.31
14.31   14.31   18.40   18.61   18.61   18.61   15.12   18.33
15: E.faecalisOG1RF_PBP2A   29.17   29.17   29.02   29.17   27.96   27.96   27.81   27.34   26.53   26.93   46.33
46.33   46.33   46.19   100.00   64.67   47.47   47.71   47.71   47.86   26.19   26.34   26.19   26.19   27.26   29.12   29.05
27.76   27.76   27.91   18.23   18.23   18.23   18.43   17.85   17.85   17.66   18.36   18.49   17.29   17.92   13.77   13.84
13.84   13.84   14.75   14.75   14.75   14.75   15.83   16.49
16: E.faeciumDO_PBP2A   28.19   28.19   28.34   28.49   26.65   26.65   26.65   26.57   26.58   26.37   49.93
49.93   49.78   49.63   64.67   100.00   47.89   47.85   47.85   47.99   28.24   28.38   28.24   28.24   28.53   29.34   28.46
30.56   30.71   30.86   18.62   18.62   18.62   18.81   17.66   17.66   17.47   18.36   17.36   15.83   15.83   14.43   14.14
14.14   14.14   16.60   16.80   16.80   16.80   17.01   18.23
17: S.pyogenesATCC-BAA-595_PBP2A   27.15   27.15   27.30   27.45   27.38   27.38   27.38   25.23   25.54   27.06   40.79
40.93   40.79   40.79   47.47   47.89   100.00   56.44   56.44   56.71   26.80   26.80   26.80   26.66   26.46   28.28   28.17
27.80   27.80   27.95   18.10   18.10   17.90   18.29   17.02   17.02   16.83   15.81   16.17   15.49   18.04   15.90   14.09
14.09   14.09   16.67   16.67   16.67   16.67   14.87   16.03
18: S.pneumoniaeR6_PBP2A   26.30   26.30   26.45   26.61   26.32   26.32   26.48   26.37   26.75   26.82   42.25
42.25   42.25   42.25   47.71   47.85   56.44   100.00   100.00   99.45   27.37   27.51   27.51   27.51   24.77   26.05   25.70
26.24   25.93   26.09   17.52   17.52   17.52   17.71   17.40   17.40   17.21   17.40   16.67   15.02   16.87   16.93   15.26
15.26   15.26   17.42   17.83   17.83   17.83   16.43   16.22
19: S.pneumoniaeD39_PBP2A   26.30   26.30   26.45   26.61   26.32   26.32   26.48   26.37   26.75   26.82   42.25
42.25   42.25   42.25   47.71   47.85   56.44   100.00   100.00   99.45   27.37   27.51   27.51   27.51   24.77   26.05   25.70
26.24   25.93   26.09   17.52   17.52   17.52   17.71   17.40   17.40   17.21   17.40   16.67   15.02   16.87   16.93   15.26
15.26   15.26   17.42   17.83   17.83   17.83   16.43   16.22
20: S.pneumoniaeNCTC7465_PBP2A   26.45   26.45   26.61   26.76   26.48   26.48   26.64   26.52   26.59   26.98   42.40
42.40   42.40   42.40   47.86   47.99   56.71   99.45   99.45   100.00   27.51   27.66   27.66   27.66   24.92   26.20   25.85
26.40   26.09   26.24   17.71   17.71   17.71   17.90   17.78   17.78   17.59   17.78   17.05   15.23   17.08   16.93   15.26
15.26   15.26   17.42   17.83   17.83   17.83   16.61   16.40
21: G.adiacensKHUD009_PBP1A   26.69   26.69   27.12   27.12   25.33   25.33   25.33   25.93   25.45   24.96   28.23
28.23   27.93   28.23   26.19   28.24   26.80   27.37   27.37   27.51   100.00   98.55   98.55   98.79   44.67   42.11   38.44

```
39.27   38.98   39.12   16.67   16.67   16.67   16.67   16.00   16.00   15.81   16.23   15.02   16.22   16.42   15.10   15.20
15.20   15.20   16.50   16.30   16.30   16.50   15.01   15.64
    22: G.adiacensIS48_PBP1A            26.69   26.69   27.12   27.12   25.18   25.18   25.18   25.77   25.45   24.96   28.08
28.08   27.79   28.08   26.34   28.38   26.80   27.51   27.51   27.66   98.55  100.00   99.52   99.76   44.67   42.17   38.44
39.12   38.84   38.98   16.85   16.85   16.85   16.85   16.00   16.00   15.81   16.60   15.21   16.22   16.42   15.10   15.52
15.52   15.52   16.50   16.30   16.30   16.50   15.33   15.81
    23: G.adiacensATCC49175_PBP1A       26.84   26.84   27.26   27.26   25.18   25.18   25.18   25.77   25.45   24.96   28.08
28.08   27.79   28.08   26.19   28.24   26.80   27.51   27.51   27.66   98.55   99.52  100.00   99.76   44.80   42.30   38.58
39.12   38.84   38.98   17.04   17.04   17.04   17.04   16.00   16.00   15.81   16.60   15.02   16.22   16.42   15.10   15.52
15.52   15.52   16.70   16.50   16.50   16.70   15.33   15.81
    24: G.adiacensGA01_PBP1A            26.69   26.69   27.12   27.12   25.18   25.18   25.18   25.77   25.45   24.96   28.08
28.08   27.79   28.08   26.19   28.24   26.66   27.51   27.51   27.66   98.79   99.76   99.76  100.00   44.80   42.30   38.58
39.27   38.98   39.12   16.85   16.85   16.85   16.85   16.00   16.00   15.81   16.60   15.21   16.22   16.42   15.10   15.52
15.52   15.52   16.50   16.30   16.30   16.50   15.33   15.81
    25: E.faecalisOG1RF_PBP1A           26.45   26.45   26.45   26.74   26.39   26.39   26.54   26.03   25.00   27.15   28.07
28.07   27.77   27.77   27.26   28.53   26.46   24.77   24.77   24.92   44.67   44.67   44.80   44.80  100.00   61.63   49.93
50.84   50.56   50.70   16.01   15.83   15.83   15.83   17.05   17.05   16.86   15.92   14.45   14.99   15.81   13.06   12.61
12.61   12.61   15.71   15.90   15.90   15.90   15.86   16.20
    26: E.faeciumDO_PBP1A               24.39   24.39   24.25   24.53   25.84   25.84   25.99   24.53   24.32   25.98   28.55
28.55   28.40   28.40   29.12   29.34   28.28   26.05   26.05   26.20   42.11   42.17   42.30   42.30   61.63  100.00   48.79
53.90   53.76   53.90   18.33   18.15   18.15   18.15   16.76   16.76   16.57   16.17   14.53   13.85   16.09   14.36   12.90
12.90   12.90   17.98   18.18   18.18   17.98   16.27   16.26
    27: S.pyogenes_ATCC-BAA-595_PBP1A   24.81   24.81   24.81   24.81   24.49   24.49   24.49   24.72   23.62   24.76   30.87
30.87   31.02   30.87   29.05   28.46   28.17   25.70   25.70   25.85   38.44   38.44   38.58   38.58   49.93   48.79  100.00
58.37   58.09   58.23   16.42   16.23   16.23   16.23   17.61   17.61   17.42   15.01   14.18   15.08   16.12   14.39   16.43
16.43   16.43   14.60   14.80   14.80   14.60   15.04   16.04
    28: S.pneumoniaeNCTC7465_PBP1A      25.00   25.00   24.85   25.00   25.04   25.04   25.04   24.47   23.38   23.27   29.34
29.34   29.50   29.50   27.76   30.56   27.80   26.24   26.24   26.40   39.27   39.12   39.12   39.27   50.84   53.90   58.37
100.00   99.30   99.58   17.23   17.04   17.04   17.04   17.33   17.33   17.14   14.91   14.07   14.38   16.04   14.08   13.27
13.27   13.27   15.90   16.10   16.10   16.10   13.25   15.70
    29: S.pneumoniaeR6_PBP1A            24.85   24.85   24.69   24.85   25.04   25.04   25.04   24.15   23.22   23.11   29.18
29.18   29.34   29.34   27.76   30.71   27.80   25.93   25.93   26.09   38.98   38.84   38.84   38.98   50.56   53.76   58.09
99.30  100.00   99.72   17.23   17.04   17.04   17.04   17.33   17.33   17.14   14.91   14.07   14.38   15.83   13.91   13.10
13.10   13.10   15.69   15.90   15.90   15.90   13.25   15.70
    30: S.pneumoniaeD39_PBP1A           25.00   25.00   24.85   25.00   25.20   25.20   25.20   24.31   23.38   23.27   29.34
29.34   29.50   29.50   27.91   30.86   27.95   26.09   26.09   26.24   39.12   38.98   38.98   39.12   50.70   53.90   58.23
99.58   99.72  100.00   17.23   17.04   17.04   17.04   17.33   17.33   17.14   14.91   14.07   14.38   16.04   13.91   13.10
13.10   13.10   15.90   16.10   16.10   16.10   13.25   15.70
    31: G.adiacensIS48_PBP2B            15.85   15.85   15.66   15.85   15.44   15.44   15.44   14.44   14.50   13.79   18.65
18.65   18.65   18.65   18.23   18.62   18.10   17.52   17.52   17.71   16.67   16.85   17.04   16.85   16.01   18.33   16.42
17.23   17.23   17.23  100.00   98.87   99.29   99.29   35.27   35.27   35.38   38.38   41.90   18.40   20.07   19.15   17.41
17.41   17.41   21.39   21.21   21.21   21.21   20.65   21.15
    32: G.adiacensGA01_PBP2B            15.85   15.85   15.66   15.85   15.62   15.62   15.62   14.63   14.50   13.79   18.85
18.85   18.85   18.85   18.23   18.62   18.10   17.52   17.52   17.71   16.67   16.85   17.04   16.85   15.83   18.15   16.23
```

```
17.04   17.04   17.04   98.87  100.00   99.29   99.43   35.42   35.42   35.53   38.82   42.05   18.59   20.26   18.97   17.58
17.58   17.58   21.39   21.21   21.21   21.21   20.65   20.98
    33: G.adiacensATCC49175_PBP2B       15.85   15.85   15.66   15.85   15.62   15.62   15.62   14.63   14.50   13.79   18.85
18.85   18.85   18.85   18.23   18.62   17.90   17.52   17.52   17.71   16.67   16.85   17.04   16.85   15.83   18.15   16.23
17.04   17.04   17.04   99.29   99.29  100.00   99.58   35.57   35.57   35.68   38.68   41.90   18.59   20.26   19.15   17.58
17.58   17.58   21.57   21.39   21.39   21.39   20.83   21.15
    34: G.adiacensKHUD009_PBP2B         15.85   15.85   15.66   15.85   15.81   15.81   15.81   14.81   14.68   13.97   19.04
19.04   19.04   19.04   18.43   18.81   18.29   17.71   17.71   17.90   16.67   16.85   17.04   16.85   15.83   18.15   16.23
17.04   17.04   17.04   99.29   99.43   99.58  100.00   35.42   35.42   35.53   38.53   41.90   18.40   20.07   18.97   17.41
17.41   17.41   21.39   21.21   21.21   21.21   20.65   20.98
    35: S.pneumoniaeR6_PBP2B            15.54   15.54   15.54   15.72   18.01   18.01   18.01   17.07   14.15   14.36   17.50
17.50   17.50   17.50   17.85   17.66   17.02   17.40   17.40   17.78   16.00   16.00   16.00   16.00   17.05   16.76   17.61
17.33   17.33   17.33   35.27   35.42   35.57   35.42  100.00  100.00   99.71   42.52   42.01   17.55   19.81   18.37   18.79
18.79   18.79   20.47   20.29   20.29   20.29   21.06   21.04
    36: S.pneumoniaeD39_PBP2B           15.54   15.54   15.54   15.72   18.01   18.01   18.01   17.07   14.15   14.36   17.50
17.50   17.50   17.50   17.85   17.66   17.02   17.40   17.40   17.78   16.00   16.00   16.00   16.00   17.05   16.76   17.61
17.33   17.33   17.33   35.27   35.42   35.57   35.42  100.00  100.00   99.71   42.52   42.01   17.55   19.81   18.37   18.79
18.79   18.79   20.47   20.29   20.29   20.29   21.06   21.04
    37: S.pneumoniaeNCTC7465_PBP2B      15.54   15.54   15.54   15.72   18.18   18.18   18.18   16.88   14.21   14.50   17.31
17.31   17.31   17.31   17.66   17.47   16.83   17.21   17.21   17.59   15.81   15.81   15.81   15.81   16.86   16.57   17.42
17.14   17.14   17.14   35.38   35.53   35.68   35.53   99.71   99.71  100.00   42.84   42.32   17.33   19.62   18.15   18.75
18.75   18.75   20.26   20.07   20.07   20.07   21.03   21.01
    38: E.faecalisOG1RF_PBP2B           18.28   18.28   17.92   18.10   18.43   18.43   18.43   18.92   16.27   16.09   18.62
18.62   18.62   18.62   18.36   18.36   15.81   17.40   17.40   17.78   16.23   16.60   16.60   16.60   15.92   16.17   15.01
14.91   14.91   14.91   38.38   38.82   38.68   38.53   42.52   42.52   42.84  100.00   54.43   20.58   21.13   18.71   20.18
20.18   20.18   18.82   18.82   18.82   18.82   21.37   21.03
    39: E.faeciumDO_PBP2B               16.94   16.94   16.76   16.94   14.75   14.75   14.75   16.51   14.78   15.54   16.44
16.44   16.44   16.44   18.49   17.36   16.17   16.67   16.67   17.05   15.02   15.21   15.02   15.21   14.45   14.53   14.18
14.07   14.07   14.07   41.90   42.05   41.90   41.90   42.01   42.01   42.32   54.43  100.00   18.77   20.40   19.16   19.76
19.76   19.76   19.60   19.60   19.60   19.60   21.82   20.78
    40: E.faecalisOG1RF_PBP4            15.51   15.51   15.51   15.71   15.66   15.66   15.66   18.11   14.17   15.46   16.97
16.97   16.97   16.97   17.29   15.83   15.49   15.02   15.02   15.23   16.22   16.22   16.22   16.22   14.99   13.85   15.08
14.38   14.38   14.38   18.40   18.59   18.59   18.40   17.55   17.55   17.33   20.58   18.77  100.00   54.28   20.43   20.43
20.43   20.43   24.34   24.34   24.34   24.53   22.34   20.80
    41: E.faeciumDO_PBP5                14.31   14.31   13.92   14.12   16.05   16.05   16.05   15.69   13.37   14.29   16.77
16.77   16.77   16.77   17.92   15.83   18.04   16.87   16.87   17.08   16.42   16.42   16.42   16.42   15.81   16.09   16.12
16.04   15.83   16.04   20.07   20.26   20.26   20.07   19.81   19.81   19.62   21.13   20.40   54.28  100.00   20.97   21.88
21.88   21.88   23.78   23.78   23.78   24.16   23.42   20.80
    42: S.pyogenesATCC-BAA-595_PBP2     13.69   13.69   13.69   13.85   12.68   12.68   12.68   14.72   13.34   12.86   14.77
14.77   14.77   14.77   13.77   14.43   15.90   16.93   16.93   16.93   15.10   15.10   15.10   15.10   13.06   14.36   14.39
14.08   13.91   13.91   19.15   18.97   19.15   18.97   18.37   18.37   18.15   18.71   19.16   20.43   20.97  100.00   54.63
54.63   54.63   35.19   35.03   35.03   35.36   39.45   39.42
    43: S.pneumoniaeR6_PBP2             14.74   14.74   14.74   14.90   13.06   13.06   13.06   13.26   11.92   12.44   14.31
14.31   14.31   14.31   13.84   14.14   14.09   15.26   15.26   15.26   15.20   15.52   15.52   15.52   12.61   12.90   16.43
```

```
13.27   13.10   13.10   17.41   17.58   17.58   17.41   18.79   18.79   18.75   20.18   19.76   20.43   21.88   54.63  100.00
100.00   99.73   36.67   36.84   36.84   36.84   38.30   40.19
    44: S.pneumoniaeD39_PBP2            14.74   14.74   14.74   14.90   13.06   13.06   13.06   13.26   11.92   12.44   14.31
14.31   14.31   14.31   13.84   14.14   14.09   15.26   15.26   15.26   15.20   15.52   15.52   15.52   12.61   12.90   16.43
13.27   13.10   13.10   17.41   17.58   17.58   17.41   18.79   18.79   18.75   20.18   19.76   20.43   21.88   54.63  100.00
100.00   99.73   36.67   36.84   36.84   36.84   38.30   40.19
    45: S.pneumoniaeNCTC7465_PBP2       14.74   14.74   14.74   14.90   13.06   13.06   13.06   13.26   11.92   12.44   14.31
14.31   14.31   14.31   13.84   14.14   14.09   15.26   15.26   15.26   15.20   15.52   15.52   15.52   12.61   12.90   16.43
13.27   13.10   13.10   17.41   17.58   17.58   17.41   18.79   18.79   18.75   20.18   19.76   20.43   21.88   54.63   99.73
99.73  100.00   36.67   36.84   36.84   36.84   38.44   40.06
    46: G.adiacensATCC49175_PBP2        16.50   16.50   16.50   16.70   13.59   13.59   13.59   15.35   13.73   13.93   18.40
18.40   18.40   18.40   14.75   16.60   16.67   17.42   17.42   17.42   16.50   16.50   16.70   16.50   15.71   17.98   14.60
15.90   15.69   15.90   21.39   21.39   21.57   21.39   20.47   20.47   20.26   18.82   19.60   24.34   23.78   35.19   36.67
36.67   36.67  100.00   98.82   98.82   99.16   38.71   39.07
    47: G.adiacensIS48_PBP2             16.50   16.50   16.50   16.70   13.59   13.59   13.59   15.35   13.73   13.93   18.61
18.61   18.61   18.61   14.75   16.80   16.67   17.83   17.83   17.83   16.30   16.30   16.50   16.30   15.90   18.18   14.80
16.10   15.90   16.10   21.21   21.21   21.39   21.21   20.29   20.29   20.07   18.82   19.60   24.34   23.78   35.03   36.84
36.84   36.84   98.82  100.00  100.00   99.33   38.54   39.07
    48: G.adiacensGA01_PBP2             16.50   16.50   16.50   16.70   13.59   13.59   13.59   15.35   13.73   13.93   18.61
18.61   18.61   18.61   14.75   16.80   16.67   17.83   17.83   17.83   16.30   16.30   16.50   16.30   15.90   18.18   14.80
16.10   15.90   16.10   21.21   21.21   21.39   21.21   20.29   20.29   20.07   18.82   19.60   24.34   23.78   35.03   36.84
36.84   36.84   98.82  100.00  100.00   99.33   38.54   39.07
    49: G.adiacensKHUD009_PBP2          16.50   16.50   16.50   16.70   13.59   13.59   13.59   15.35   13.73   13.93   18.61
18.61   18.61   18.61   14.75   16.80   16.67   17.83   17.83   17.83   16.50   16.50   16.70   16.50   15.90   17.98   14.60
16.10   15.90   16.10   21.21   21.21   21.39   21.21   20.29   20.29   20.07   18.82   19.60   24.53   24.16   35.36   36.84
36.84   36.84   99.16   99.33   99.33  100.00   38.54   39.07
    50: E.faecalisOG1RF_PBP2            14.93   14.93   14.93   15.09   12.80   12.80   12.80   12.10   13.14   13.31   15.12
15.12   15.12   15.12   15.83   17.01   14.87   16.43   16.43   16.61   15.01   15.33   15.33   15.33   15.86   16.27   15.04
13.25   13.25   13.25   20.65   20.65   20.83   20.65   21.06   21.06   21.03   21.37   21.82   22.34   23.42   39.45   38.30
38.30   38.44   38.71   38.54   38.54   38.54  100.00   59.28
    51: E.faeciumDO_PBP2                17.32   17.32   17.16   17.32   15.51   15.51   15.51   14.90   14.41   14.55   18.15
18.33   18.33   18.33   16.49   18.23   16.03   16.22   16.22   16.40   15.64   15.81   15.81   15.81   16.20   16.26   16.04
15.70   15.70   15.70   21.15   20.98   21.15   20.98   21.04   21.04   21.01   21.03   20.78   20.80   20.80   39.42   40.19
40.19   40.06   39.07   39.07   39.07   39.07   59.28  100.00
```

Table S1. PBPs amino acid sequences multi alignment Percent Identity Matrix (PIM Matrix), created by Clustal2.1.
