# Supplementary material for: Target Fidelity and Failure: Structure–Activity Relationship of High-Molecular-Mass Penicillin-Binding Proteins (HMM-PBPs) in Refractory Granulicatella adiacens Endocarditis
Source: Antibiotics (Basel). 2026 Feb 5;15(2):168. doi: 10.3390/antibiotics15020168 (PMC12937247; doi:10.3390/antibiotics15020168)
Supplement: Supplementary file 1 [file antibiotics-15-00168-s001.zip › Supplementary files/Table S2. pbp genes mutations and aminoacidic substitutions of G. adiacens clinical isolate IS48 .pdf]

NT_POS	AA_POS	EFFECT	LOCUS_TAG	GENE	PRODUCT
21/2532	7/843	synonymous_variant c.21C>T p.Thr7Thr	NQ540_RS06930		transglycosylase domain-containing protein
281/2532	94/843	missense_variant c.281_285delATGATinsGTGAC p.Asn94Ser	NQ540_RS06930		transglycosylase domain-containing protein
405/2532	135/843	synonymous_variant c.405C>T p.His135His	NQ540_RS06930		transglycosylase domain-containing protein
411/2532	137/843	synonymous_variant c.411_414delAATCinsGATT p.139	NQ540_RS06930		transglycosylase domain-containing protein
534/2532	178/843	synonymous_variant c.534C>T p.Val178Val	NQ540_RS06930		transglycosylase domain-containing protein
546/2532	182/843	synonymous_variant c.546T>A p.Arg182Arg	NQ540_RS06930		transglycosylase domain-containing protein
555/2532	185/843	synonymous_variant c.555C>T p.Asn185Asn	NQ540_RS06930		transglycosylase domain-containing protein
721/2532	241/843	synonymous_variant c.721T>C p.Leu241Leu	NQ540_RS06930		transglycosylase domain-containing protein
801/2532	267/843	synonymous_variant c.801G>A p.Lys267Lys	NQ540_RS06930		transglycosylase domain-containing protein
1236/2532	412/843	synonymous_variant c.1236C>A p.Thr412Thr	NQ540_RS06930		transglycosylase domain-containing protein
1251/2532	417/843	synonymous_variant c.1251G>A p.Glu417Glu	NQ540_RS06930		transglycosylase domain-containing protein
1284/2532	428/843	synonymous_variant c.1284C>T p.Asn428Asn	NQ540_RS06930		transglycosylase domain-containing protein
1374/2532	458/843	synonymous_variant c.1374G>T p.Gly458Gly	NQ540_RS06930		transglycosylase domain-containing protein
1434/2532	478/843	synonymous_variant c.1434A>T p.Pro478Pro	NQ540_RS06930		transglycosylase domain-containing protein
1449/2532	483/843	synonymous_variant c.1449T>A p.Pro483Pro	NQ540_RS06930		transglycosylase domain-containing protein
1488/2532	496/843	synonymous_variant c.1488T>C p.Tyr496Tyr	NQ540_RS06930		transglycosylase domain-containing protein
1497/2532	499/843	synonymous_variant c.1497_1500delAACGinsGACA p.501	NQ540_RS06930		transglycosylase domain-containing protein
1521/2532	507/843	synonymous_variant c.1521A>G p.Thr507Thr	NQ540_RS06930		transglycosylase domain-containing protein
1578/2532	526/843	synonymous_variant c.1578C>T p.Ile526Ile	NQ540_RS06930		transglycosylase domain-containing protein
1641/2532	547/843	synonymous_variant c.1641_1644delTATTinsCATC p.549	NQ540_RS06930		transglycosylase domain-containing protein
1653/2532	551/843	synonymous_variant c.1653C>T p.Ile551Ile	NQ540_RS06930		transglycosylase domain-containing protein
1710/2532	570/843	synonymous_variant c.1710G>T p.Pro570Pro	NQ540_RS06930		transglycosylase domain-containing protein
1737/2532	579/843	synonymous_variant c.1737T>C p.Phe579Phe	NQ540_RS06930		transglycosylase domain-containing protein
1743/2532	581/843	synonymous_variant c.1743G>T p.Thr581Thr	NQ540_RS06930		transglycosylase domain-containing protein
1767/2532	589/843	synonymous_variant c.1767T>C p.His589His	NQ540_RS06930		transglycosylase domain-containing protein
1773/2532	591/843	synonymous_variant c.1773T>A p.Ala591Ala	NQ540_RS06930		transglycosylase domain-containing protein
1785/2532	595/843	synonymous_variant c.1785A>G p.Glu595Glu	NQ540_RS06930		transglycosylase domain-containing protein
1797/2532	599/843	synonymous_variant c.1797C>T p.Asp599Asp	NQ540_RS06930		transglycosylase domain-containing protein
1810/2532	604/843	missense_variant c.1810A>G p.Ile604Val	NQ540_RS06930		transglycosylase domain-containing protein
1824/2532	608/843	synonymous_variant c.1824C>T p.His608His	NQ540_RS06930		transglycosylase domain-containing protein
1845/2532	615/843	synonymous_variant c.1845C>T p.Val615Val	NQ540_RS06930		transglycosylase domain-containing protein
1854/2532	618/843	missense_variant c.1854A>C p.Glu618Asp	NQ540_RS06930		transglycosylase domain-containing protein
1861/2532	621/843	missense_variant c.1861T>G p.Ser621Ala	NQ540_RS06930		transglycosylase domain-containing protein
1890/2532	630/843	synonymous_variant c.1890_1893delTATTinsCATC p.632	NQ540_RS06930		transglycosylase domain-containing protein
1950/2532	650/843	synonymous_variant c.1950A>G p.Leu650Leu	NQ540_RS06930		transglycosylase domain-containing protein
1966/2532	656/843	missense_variant c.1966A>G p.Thr656Ala	NQ540_RS06930		transglycosylase domain-containing protein
2142/2532	714/843	synonymous_variant c.2142A>T p.Ala714Ala	NQ540_RS06930		transglycosylase domain-containing protein
2265/2532	755/843	synonymous_variant c.2265T>C p.Gly755Gly	NQ540_RS06930		transglycosylase domain-containing protein
2298/2532	766/843	synonymous_variant c.2298T>C p.Thr766Thr	NQ540_RS06930		transglycosylase domain-containing protein
2313/2532	771/843	synonymous_variant c.2313G>A p.Lys771Lys	NQ540_RS06930		transglycosylase domain-containing protein
2394/2532	798/843	synonymous_variant c.2394T>C p.Ala798Ala	NQ540_RS06930		transglycosylase domain-containing protein
2403/2532	801/843	synonymous_variant c.2403G>A p.Lys801Lys	NQ540_RS06930		transglycosylase domain-containing protein
2409/2532	803/843	synonymous_variant c.2409A>G p.Glu803Glu	NQ540_RS06930		transglycosylase domain-containing protein
2443/2532	815/843	conservative_inframe_insertion c.2442_2443insAACGAGAAAAAA p.Lys814_Asp815insAsnGluLysLys	NQ540_RS06930		transglycosylase domain-containing protein
2448/2532	816/843	synonymous_variant c.2448G>A p.Glu816Glu	NQ540_RS06930		transglycosylase domain-containing protein
2487/2532	829/843	synonymous_variant c.2487C>T p.Thr829Thr	NQ540_RS06930		transglycosylase domain-containing protein
2502/2532	834/843	synonymous_variant c.2502G>T p.Ser834Ser	NQ540_RS06930		transglycosylase domain-containing protein

2510/2532	837/843	missense_variant c.2510G>A p.Ser837Asn	NQ540_RS06930		transglycosylase domain-containing protein
2517/2532	839/843	synonymous_variant c.2517A>G p.Thr839Thr	NQ540_RS06930		transglycosylase domain-containing protein
18/2484	6/827	synonymous_variant c.18_21delTGGAinsCGGC p.8	NQ540_RS05265	*pbp* 1A	transglycosylase domain-containing protein
142/2484	48/827	missense_variant c.142T>G p.Ser48Ala	NQ540_RS05265	*pbp* 1A	transglycosylase domain-containing protein
249/2484	83/827	synonymous_variant c.249A>G p.Leu83Leu	NQ540_RS05265	*pbp* 1A	transglycosylase domain-containing protein
528/2484	176/827	synonymous_variant c.528A>G p.Ala176Ala	NQ540_RS05265	*pbp* 1A	transglycosylase domain-containing protein
687/2484	229/827	synonymous_variant c.687C>T p.Ala229Ala	NQ540_RS05265	*pbp* 1A	transglycosylase domain-containing protein
798/2484	266/827	synonymous_variant c.798C>T p.Asn266Asn	NQ540_RS05265	*pbp* 1A	transglycosylase domain-containing protein
912/2484	304/827	synonymous_variant c.912C>T p.Val304Val	NQ540_RS05265	*pbp* 1A	transglycosylase domain-containing protein
1042/2484	348/827	missense_variant c.1042G>A p.Val348Ile	NQ540_RS05265	*pbp* 1A	transglycosylase domain-containing protein
1098/2484	366/827	synonymous_variant c.1098_1099delTCinsCA p.368	NQ540_RS05265	*pbp* 1A	transglycosylase domain-containing protein
1143/2484	381/827	synonymous_variant c.1143C>T p.Tyr381Tyr	NQ540_RS05265	*pbp* 1A	transglycosylase domain-containing protein
1164/2484	388/827	synonymous_variant c.1164A>G p.Gln388Gln	NQ540_RS05265	*pbp* 1A	transglycosylase domain-containing protein
1197/2484	399/827	synonymous_variant c.1197T>C p.Ile399Ile	NQ540_RS05265	*pbp* 1A	transglycosylase domain-containing protein
1371/2484	457/827	synonymous_variant c.1371T>G p.Ala457Ala	NQ540_RS05265	*pbp* 1A	transglycosylase domain-containing protein
1413/2484	471/827	missense_variant c.1413A>T p.Lys471Asn	NQ540_RS05265	*pbp* 1A	transglycosylase domain-containing protein
1488/2484	496/827	synonymous_variant c.1488A>T p.Ser496Ser	NQ540_RS05265	*pbp* 1A	transglycosylase domain-containing protein
1742/2484	581/827	missense_variant c.1742C>A p.Thr581Asn	NQ540_RS05265	*pbp* 1A	transglycosylase domain-containing protein
1767/2484	589/827	synonymous_variant c.1767A>C p.Gly589Gly	NQ540_RS05265	*pbp* 1A	transglycosylase domain-containing protein
1785/2484	595/827	synonymous_variant c.1785C>T p.Asp595Asp	NQ540_RS05265	*pbp* 1A	transglycosylase domain-containing protein
1860/2484	620/827	synonymous_variant c.1860G>A p.Pro620Pro	NQ540_RS05265	*pbp* 1A	transglycosylase domain-containing protein
2061/2484	687/827	synonymous_variant c.2061A>G p.Thr687Thr	NQ540_RS05265	*pbp* 1A	transglycosylase domain-containing protein
2067/2484	689/827	synonymous_variant c.2067A>G p.Leu689Leu	NQ540_RS05265	*pbp* 1A	transglycosylase domain-containing protein
2073/2484	691/827	synonymous_variant c.2073T>A p.Val691Val	NQ540_RS05265	*pbp* 1A	transglycosylase domain-containing protein
2121/2484	707/827	synonymous_variant c.2121A>C p.Ile707Ile	NQ540_RS05265	*pbp* 1A	transglycosylase domain-containing protein
2340/2484	780/827	synonymous_variant c.2340A>T p.Ser780Ser	NQ540_RS05265	*pbp* 1A	transglycosylase domain-containing protein
2367/2484	789/827	synonymous_variant c.2367C>T p.Ile789Ile	NQ540_RS05265	*pbp* 1A	transglycosylase domain-containing protein
2430/2484	810/827	synonymous_variant c.2430T>C p.Asn810Asn	NQ540_RS05265	*pbp* 1A	transglycosylase domain-containing protein
28/2163	10/720	missense_variant c.28T>C p.Tyr10His	NQ540_RS03315	*pbp* 2A	transglycosylase domain-containing protein
49/2163	17/720	missense_variant c.49A>G p.Asn17Asp	NQ540_RS03315	*pbp* 2A	transglycosylase domain-containing protein
104/2163	35/720	missense_variant c.104A>C p.Gln35Pro	NQ540_RS03315	*pbp* 2A	transglycosylase domain-containing protein
135/2163	45/720	synonymous_variant c.135G>A p.Lys45Lys	NQ540_RS03315	*pbp* 2A	transglycosylase domain-containing protein
177/2163	59/720	synonymous_variant c.177C>G p.Val59Val	NQ540_RS03315	*pbp* 2A	transglycosylase domain-containing protein
201/2163	67/720	missense_variant c.201G>T p.Met67Ile	NQ540_RS03315	*pbp* 2A	transglycosylase domain-containing protein
267/2163	89/720	synonymous_variant c.267C>T p.Gly89Gly	NQ540_RS03315	*pbp* 2A	transglycosylase domain-containing protein
300/2163	100/720	synonymous_variant c.300T>C p.Val100Val	NQ540_RS03315	*pbp* 2A	transglycosylase domain-containing protein
309/2163	103/720	synonymous_variant c.309T>C p.Arg103Arg	NQ540_RS03315	*pbp* 2A	transglycosylase domain-containing protein
336/2163	112/720	synonymous_variant c.336T>C p.Asn112Asn	NQ540_RS03315	*pbp* 2A	transglycosylase domain-containing protein
342/2163	114/720	synonymous_variant c.342T>C p.Asn114Asn	NQ540_RS03315	*pbp* 2A	transglycosylase domain-containing protein
405/2163	135/720	synonymous_variant c.405T>C p.Thr135Thr	NQ540_RS03315	*pbp* 2A	transglycosylase domain-containing protein
468/2163	156/720	synonymous_variant c.468C>T p.Phe156Phe	NQ540_RS03315	*pbp* 2A	transglycosylase domain-containing protein
585/2163	195/720	synonymous_variant c.585C>A p.Leu195Leu	NQ540_RS03315	*pbp* 2A	transglycosylase domain-containing protein
663/2163	221/720	synonymous_variant c.663_669delCAATGGCinsTAACGGT p.224	NQ540_RS03315	*pbp* 2A	transglycosylase domain-containing protein
684/2163	228/720	synonymous_variant c.684A>G p.Glu228Glu	NQ540_RS03315	*pbp* 2A	transglycosylase domain-containing protein
729/2163	243/720	synonymous_variant c.729_732delCATCinsGATT p.245	NQ540_RS03315	*pbp* 2A	transglycosylase domain-containing protein
852/2163	284/720	synonymous_variant c.852G>A p.Gly284Gly	NQ540_RS03315	*pbp* 2A	transglycosylase domain-containing protein
882/2163	294/720	synonymous_variant c.882_885delCGGAinsTGGG p.296	NQ540_RS03315	*pbp* 2A	transglycosylase domain-containing protein
957/2163	319/720	synonymous_variant c.957_960delTGTCinsCGTT p.321	NQ540_RS03315	*pbp* 2A	transglycosylase domain-containing protein

1026/2163	342/720	synonymous_variant c.1026C>A p.Ile342Ile	NQ540_RS03315	*pbp* 2A	transglycosylase domain-containing protein
1032/2163	344/720	synonymous_variant c.1032T>A p.Thr344Thr	NQ540_RS03315	*pbp* 2A	transglycosylase domain-containing protein
1107/2163	369/720	synonymous_variant c.1107C>T p.Asp369Asp	NQ540_RS03315	*pbp* 2A	transglycosylase domain-containing protein
1134/2163	378/720	synonymous_variant c.1134G>A p.Val378Val	NQ540_RS03315	*pbp* 2A	transglycosylase domain-containing protein
1149/2163	383/720	synonymous_variant c.1149G>A p.Gln383Gln	NQ540_RS03315	*pbp* 2A	transglycosylase domain-containing protein
1155/2163	385/720	synonymous_variant c.1155A>G p.Gly385Gly	NQ540_RS03315	*pbp* 2A	transglycosylase domain-containing protein
1161/2163	387/720	synonymous_variant c.1161T>C p.Val387Val	NQ540_RS03315	*pbp* 2A	transglycosylase domain-containing protein
1281/2163	427/720	synonymous_variant c.1281A>T p.Ala427Ala	NQ540_RS03315	*pbp* 2A	transglycosylase domain-containing protein
1314/2163	438/720	synonymous_variant c.1314T>C p.Leu438Leu	NQ540_RS03315	*pbp* 2A	transglycosylase domain-containing protein
1389/2163	463/720	synonymous_variant c.1389T>G p.Val463Val	NQ540_RS03315	*pbp* 2A	transglycosylase domain-containing protein
1404/2163	468/720	synonymous_variant c.1404G>T p.Ala468Ala	NQ540_RS03315	*pbp* 2A	transglycosylase domain-containing protein
1428/2163	476/720	synonymous_variant c.1428A>T p.Pro476Pro	NQ540_RS03315	*pbp* 2A	transglycosylase domain-containing protein
1434/2163	478/720	synonymous_variant c.1434G>A p.Val478Val	NQ540_RS03315	*pbp* 2A	transglycosylase domain-containing protein
1524/2163	508/720	synonymous_variant c.1524G>A p.Gly508Gly	NQ540_RS03315	*pbp* 2A	transglycosylase domain-containing protein
1578/2163	526/720	synonymous_variant c.1578C>T p.Ala526Ala	NQ540_RS03315	*pbp* 2A	transglycosylase domain-containing protein
1605/2163	535/720	synonymous_variant c.1605C>T p.Val535Val	NQ540_RS03315	*pbp* 2A	transglycosylase domain-containing protein
1662/2163	554/720	synonymous_variant c.1662C>T p.Val554Val	NQ540_RS03315	*pbp* 2A	transglycosylase domain-containing protein
1695/2163	565/720	synonymous_variant c.1695G>A p.Thr565Thr	NQ540_RS03315	*pbp* 2A	transglycosylase domain-containing protein
1732/2163	578/720	synonymous_variant c.1732T>C p.Leu578Leu	NQ540_RS03315	*pbp* 2A	transglycosylase domain-containing protein
1797/2163	599/720	synonymous_variant c.1797C>A p.Gly599Gly	NQ540_RS03315	*pbp* 2A	transglycosylase domain-containing protein
1920/2163	640/720	synonymous_variant c.1920T>C p.Ser640Ser	NQ540_RS03315	*pbp* 2A	transglycosylase domain-containing protein
1926/2163	642/720	synonymous_variant c.1926T>A p.Ser642Ser	NQ540_RS03315	*pbp* 2A	transglycosylase domain-containing protein
1935/2163	645/720	synonymous_variant c.1935C>T p.Ser645Ser	NQ540_RS03315	*pbp* 2A	transglycosylase domain-containing protein
1983/2163	661/720	synonymous_variant c.1983C>G p.Leu661Leu	NQ540_RS03315	*pbp* 2A	transglycosylase domain-containing protein
2001/2163	667/720	synonymous_variant c.2001_2004delAGCCinsGGCA p.669	NQ540_RS03315	*pbp* 2A	transglycosylase domain-containing protein
2028/2163	676/720	synonymous_variant c.2028G>C p.Thr676Thr	NQ540_RS03315	*pbp* 2A	transglycosylase domain-containing protein
2035/2163	679/720	missense_variant c.2035T>G p.Ser679Ala	NQ540_RS03315	*pbp* 2A	transglycosylase domain-containing protein
2043/2163	681/720	synonymous_variant c.2043G>A p.Gln681Gln	NQ540_RS03315	*pbp* 2A	transglycosylase domain-containing protein
2070/2163	690/720	missense_variant c.2070T>A p.Asn690Lys	NQ540_RS03315	*pbp* 2A	transglycosylase domain-containing protein
2082/2163	694/720	synonymous_variant c.2082T>C p.Asp694Asp	NQ540_RS03315	*pbp* 2A	transglycosylase domain-containing protein
2132/2163	711/720	missense_variant c.2132A>G p.Lys711Arg	NQ540_RS03315	*pbp* 2A	transglycosylase domain-containing protein
2046/2124	682/707	synonymous_variant c.2046T>G p.Ala682Ala	NQ540_RS07070	*pbp*	peptidoglycan D,D-transpeptidase FtsI family protein
2000/2124	667/707	missense_variant c.2000C>T p.Ser667Leu	NQ540_RS07070	*pbp*	peptidoglycan D,D-transpeptidase FtsI family protein
1986/2124	662/707	synonymous_variant c.1986T>C p.Tyr662Tyr	NQ540_RS07070	*pbp*	peptidoglycan D,D-transpeptidase FtsI family protein
1968/2124	656/707	synonymous_variant c.1968A>G p.Val656Val	NQ540_RS07070	*pbp*	peptidoglycan D,D-transpeptidase FtsI family protein
1865/2124	622/707	missense_variant c.1865C>T p.Ala622Val	NQ540_RS07070	*pbp*	peptidoglycan D,D-transpeptidase FtsI family protein
1833/2124	611/707	synonymous_variant c.1833C>T p.Tyr611Tyr	NQ540_RS07070	*pbp*	peptidoglycan D,D-transpeptidase FtsI family protein
1777/2124	592/707	synonymous_variant c.1776_1777delACinsGT p.594	NQ540_RS07070	*pbp*	peptidoglycan D,D-transpeptidase FtsI family protein
1758/2124	586/707	synonymous_variant c.1758G>A p.Leu586Leu	NQ540_RS07070	*pbp*	peptidoglycan D,D-transpeptidase FtsI family protein
1746/2124	581/707	synonymous_variant c.1743_1746delAGAGinsCGAA p.583	NQ540_RS07070	*pbp*	peptidoglycan D,D-transpeptidase FtsI family protein
1734/2124	578/707	synonymous_variant c.1734T>C p.Thr578Thr	NQ540_RS07070	*pbp*	peptidoglycan D,D-transpeptidase FtsI family protein
1704/2124	567/707	synonymous_variant c.1701_1704delTGAAinsAGAG p.569	NQ540_RS07070	*pbp*	peptidoglycan D,D-transpeptidase FtsI family protein
1626/2124	541/707	synonymous_variant c.1623_1626delTAACinsAAAT p.543	NQ540_RS07070	*pbp*	peptidoglycan D,D-transpeptidase FtsI family protein
1617/2124	538/707	synonymous_variant c.1614_1617delTACGinsGACC p.540	NQ540_RS07070	*pbp*	peptidoglycan D,D-transpeptidase FtsI family protein
1599/2124	533/707	synonymous_variant c.1599G>A p.Leu533Leu	NQ540_RS07070	*pbp*	peptidoglycan D,D-transpeptidase FtsI family protein
1537/2124	513/707	missense_variant c.1537G>A p.Asp513Asn	NQ540_RS07070	*pbp*	peptidoglycan D,D-transpeptidase FtsI family protein
1512/2124	504/707	synonymous_variant c.1512A>G p.Gly504Gly	NQ540_RS07070	*pbp*	peptidoglycan D,D-transpeptidase FtsI family protein
1503/2124	500/707	synonymous_variant c.1500_1503delACCAinsTCCG p.502	NQ540_RS07070	*pbp*	peptidoglycan D,D-transpeptidase FtsI family protein

1491/2124	496/707	synonymous_variant c.1488_1491delTGGGinsCGGA p.498	NQ540_RS07070	*pbp*	peptidoglycan D,D-transpeptidase FtsI family protein
1473/2124	491/707	synonymous_variant c.1473A>T p.Gly491Gly	NQ540_RS07070	*pbp*	peptidoglycan D,D-transpeptidase FtsI family protein
1467/2124	489/707	synonymous_variant c.1467A>G p.Gln489Gln	NQ540_RS07070	*pbp*	peptidoglycan D,D-transpeptidase FtsI family protein
1455/2124	485/707	synonymous_variant c.1455A>G p.Glu485Glu	NQ540_RS07070	*pbp*	peptidoglycan D,D-transpeptidase FtsI family protein
1449/2124	483/707	synonymous_variant c.1449G>A p.Leu483Leu	NQ540_RS07070	*pbp*	peptidoglycan D,D-transpeptidase FtsI family protein
1410/2124	470/707	synonymous_variant c.1410C>T p.Gly470Gly	NQ540_RS07070	*pbp*	peptidoglycan D,D-transpeptidase FtsI family protein
1386/2124	462/707	synonymous_variant c.1386T>C p.Gly462Gly	NQ540_RS07070	*pbp*	peptidoglycan D,D-transpeptidase FtsI family protein
1344/2124	448/707	synonymous_variant c.1344T>C p.Arg448Arg	NQ540_RS07070	*pbp*	peptidoglycan D,D-transpeptidase FtsI family protein
1323/2124	441/707	synonymous_variant c.1323C>A p.Thr441Thr	NQ540_RS07070	*pbp*	peptidoglycan D,D-transpeptidase FtsI family protein
1221/2124	407/707	synonymous_variant c.1221A>T p.Ala407Ala	NQ540_RS07070	*pbp*	peptidoglycan D,D-transpeptidase FtsI family protein
1202/2124	401/707	missense_variant c.1202C>T p.Ser401Leu	NQ540_RS07070	*pbp*	peptidoglycan D,D-transpeptidase FtsI family protein
1194/2124	398/707	synonymous_variant c.1194T>G p.Thr398Thr	NQ540_RS07070	*pbp*	peptidoglycan D,D-transpeptidase FtsI family protein
990/2124	330/707	synonymous_variant c.990C>T p.Ala330Ala	NQ540_RS07070	*pbp*	peptidoglycan D,D-transpeptidase FtsI family protein
963/2124	321/707	synonymous_variant c.963A>G p.Glu321Glu	NQ540_RS07070	*pbp*	peptidoglycan D,D-transpeptidase FtsI family protein
936/2124	312/707	synonymous_variant c.936T>C p.Ser312Ser	NQ540_RS07070	*pbp*	peptidoglycan D,D-transpeptidase FtsI family protein
798/2124	266/707	synonymous_variant c.798C>T p.Asn266Asn	NQ540_RS07070	*pbp*	peptidoglycan D,D-transpeptidase FtsI family protein
786/2124	262/707	synonymous_variant c.786A>C p.Gly262Gly	NQ540_RS07070	*pbp*	peptidoglycan D,D-transpeptidase FtsI family protein
778/2124	259/707	missense_variant c.777_778delATinsTG p.Ser260Ala	NQ540_RS07070	*pbp*	peptidoglycan D,D-transpeptidase FtsI family protein
750/2124	249/707	synonymous_variant c.747_750delATTGinsGCTA p.251	NQ540_RS07070	*pbp*	peptidoglycan D,D-transpeptidase FtsI family protein
732/2124	244/707	synonymous_variant c.732T>C p.Ser244Ser	NQ540_RS07070	*pbp*	peptidoglycan D,D-transpeptidase FtsI family protein
702/2124	234/707	synonymous_variant c.702C>T p.Asp234Asp	NQ540_RS07070	*pbp*	peptidoglycan D,D-transpeptidase FtsI family protein
660/2124	219/707	synonymous_variant c.657_660delAGTAinsCGTG p.221	NQ540_RS07070	*pbp*	peptidoglycan D,D-transpeptidase FtsI family protein
612/2124	204/707	synonymous_variant c.612G>A p.Glu204Glu	NQ540_RS07070	*pbp*	peptidoglycan D,D-transpeptidase FtsI family protein
324/2124	108/707	synonymous_variant c.324T>C p.Thr108Thr	NQ540_RS07070	*pbp*	peptidoglycan D,D-transpeptidase FtsI family protein
249/2124	83/707	synonymous_variant c.249T>A p.Pro83Pro	NQ540_RS07070	*pbp*	peptidoglycan D,D-transpeptidase FtsI family protein
240/2124	80/707	synonymous_variant c.240G>A p.Gly80Gly	NQ540_RS07070	*pbp*	peptidoglycan D,D-transpeptidase FtsI family protein
198/2124	66/707	synonymous_variant c.198A>T p.Pro66Pro	NQ540_RS07070	*pbp*	peptidoglycan D,D-transpeptidase FtsI family protein
183/2124	61/707	synonymous_variant c.183T>C p.Ser61Ser	NQ540_RS07070	*pbp*	peptidoglycan D,D-transpeptidase FtsI family protein
144/2124	48/707	synonymous_variant c.144A>G p.Glu48Glu	NQ540_RS07070	*pbp*	peptidoglycan D,D-transpeptidase FtsI family protein
129/2124	43/707	synonymous_variant c.129G>A p.Leu43Leu	NQ540_RS07070	*pbp*	peptidoglycan D,D-transpeptidase FtsI family protein
123/2124	41/707	synonymous_variant c.123T>C p.Val41Val	NQ540_RS07070	*pbp*	peptidoglycan D,D-transpeptidase FtsI family protein
117/2124	39/707	synonymous_variant c.117A>G p.Ala39Ala	NQ540_RS07070	*pbp*	peptidoglycan D,D-transpeptidase FtsI family protein
78/2124	26/707	synonymous_variant c.78A>T p.Ile26Ile	NQ540_RS07070	*pbp*	peptidoglycan D,D-transpeptidase FtsI family protein
1715/1788	572/595	missense_variant c.1715G>T p.Gly572Val	NQ540_RS06305	*pbp* C	peptidoglycan D,D-transpeptidase FtsI family protein
1686/1788	562/595	synonymous_variant c.1686C>A p.Thr562Thr	NQ540_RS06305	*pbp* C	peptidoglycan D,D-transpeptidase FtsI family protein
1650/1788	550/595	synonymous_variant c.1650C>T p.Ala550Ala	NQ540_RS06305	*pbp* C	peptidoglycan D,D-transpeptidase FtsI family protein
1579/1788	527/595	missense_variant c.1579A>T p.Ile527Phe	NQ540_RS06305	*pbp* C	peptidoglycan D,D-transpeptidase FtsI family protein
1539/1788	513/595	synonymous_variant c.1539C>T p.Ile513Ile	NQ540_RS06305	*pbp* C	peptidoglycan D,D-transpeptidase FtsI family protein
1410/1788	470/595	synonymous_variant c.1410C>T p.Thr470Thr	NQ540_RS06305	*pbp* C	peptidoglycan D,D-transpeptidase FtsI family protein
1401/1788	467/595	missense_variant c.1401G>C p.Glu467Asp	NQ540_RS06305	*pbp* C	peptidoglycan D,D-transpeptidase FtsI family protein
1172/1788	391/595	missense_variant c.1172C>T p.Ala391Val	NQ540_RS06305	*pbp* C	peptidoglycan D,D-transpeptidase FtsI family protein
1140/1788	380/595	synonymous_variant c.1140G>T p.Val380Val	NQ540_RS06305	*pbp* C	peptidoglycan D,D-transpeptidase FtsI family protein
1119/1788	373/595	synonymous_variant c.1119C>T p.Gly373Gly	NQ540_RS06305	*pbp* C	peptidoglycan D,D-transpeptidase FtsI family protein
1104/1788	368/595	synonymous_variant c.1104A>C p.Ile368Ile	NQ540_RS06305	*pbp* C	peptidoglycan D,D-transpeptidase FtsI family protein
1011/1788	337/595	synonymous_variant c.1011G>A p.Gly337Gly	NQ540_RS06305	*pbp* C	peptidoglycan D,D-transpeptidase FtsI family protein
948/1788	316/595	synonymous_variant c.948T>C p.Asp316Asp	NQ540_RS06305	*pbp* C	peptidoglycan D,D-transpeptidase FtsI family protein
720/1788	239/595	synonymous_variant c.717_720delGACCinsAACG p.241	NQ540_RS06305	*pbp* C	peptidoglycan D,D-transpeptidase FtsI family protein
690/1788	230/595	synonymous_variant c.690A>C p.Gly230Gly	NQ540_RS06305	*pbp* C	peptidoglycan D,D-transpeptidase FtsI family protein

648/1788	216/595	synonymous_variant c.648T>C p.Thr216Thr	NQ540_RS06305	*pbp* C	peptidoglycan D,D-transpeptidase FtsI family protein
510/1788	170/595	synonymous_variant c.510G>A p.Ala170Ala	NQ540_RS06305	*pbp* C	peptidoglycan D,D-transpeptidase FtsI family protein
483/1788	161/595	synonymous_variant c.483A>C p.Pro161Pro	NQ540_RS06305	*pbp* C	peptidoglycan D,D-transpeptidase FtsI family protein
392/1788	131/595	missense_variant c.392A>G p.Asn131Ser	NQ540_RS06305	*pbp* C	peptidoglycan D,D-transpeptidase FtsI family protein
277/1788	93/595	missense_variant c.277A>G p.Asn93Asp	NQ540_RS06305	*pbp* C	peptidoglycan D,D-transpeptidase FtsI family protein
207/1788	69/595	synonymous_variant c.207A>C p.Thr69Thr	NQ540_RS06305	*pbp* C	peptidoglycan D,D-transpeptidase FtsI family protein
183/1788	61/595	synonymous_variant c.183A>G p.Arg61Arg	NQ540_RS06305	*pbp* C	peptidoglycan D,D-transpeptidase FtsI family protein
61/1788	21/595	missense_variant c.61A>T p.Met21Leu	NQ540_RS06305	*pbp* C	peptidoglycan D,D-transpeptidase FtsI family protein

Table S2. *pbp* genes mutations and aminoacidic substitutions of *G. adiacens* clinical isolate IS48
