# Supplementary material for: Target Fidelity and Failure: Structure–Activity Relationship of High-Molecular-Mass Penicillin-Binding Proteins (HMM-PBPs) in Refractory Granulicatella adiacens Endocarditis
Source: Antibiotics (Basel). 2026 Feb 5;15(2):168. doi: 10.3390/antibiotics15020168 (PMC12937247; doi:10.3390/antibiotics15020168)
Supplement: Supplementary file 1 [file antibiotics-15-00168-s001.zip › Supplementary files/Figure S2a ATCC 49175 PBP bocillin-labeled profiles on SDSΓÇôPAGE gel and blue coomassie staining.pdf]

G. adiacens ATCC 49175

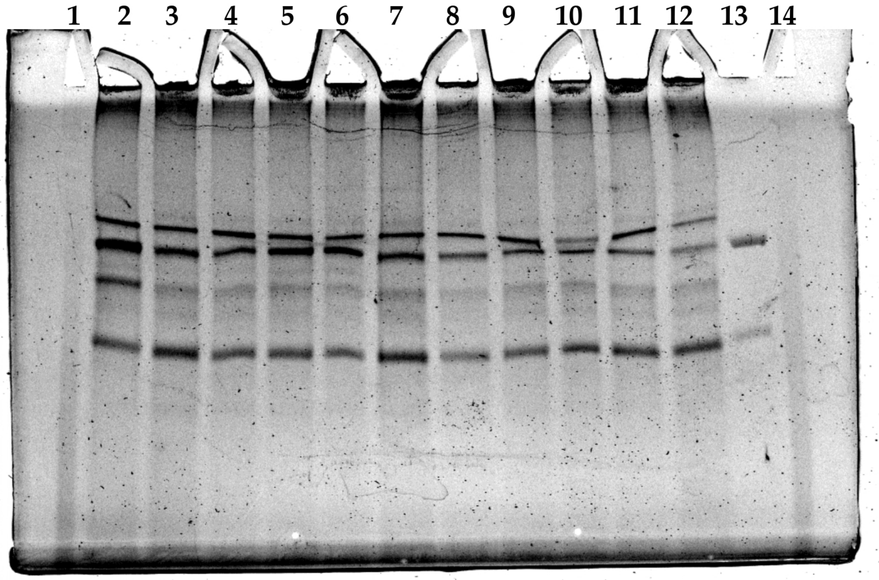

(a)

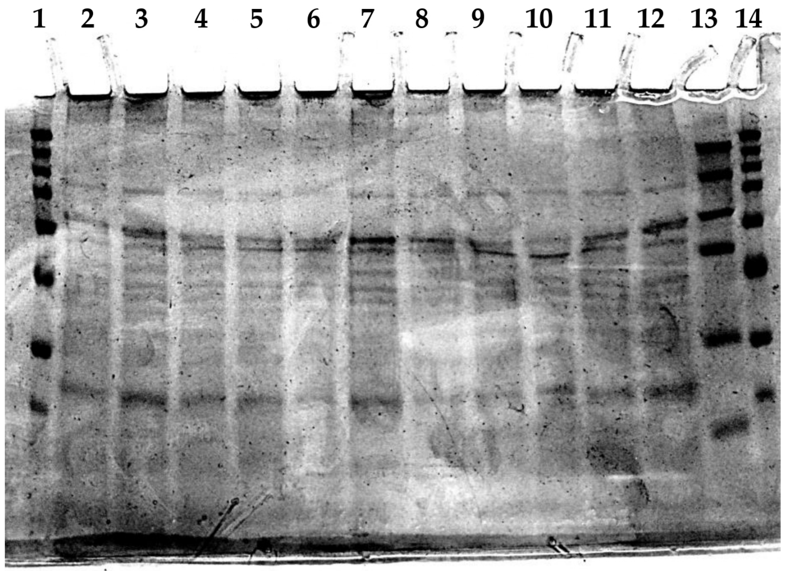

(b)

Loading scheme:

1. Ladder Precision Plus Protein Dual Color Standards (BIORAD)
2. *G. adiacens* ATCC 49175 not treated
3. *G. adiacens* ATCC 49175 BPR 1/2X MIC
4. *G. adiacens* ATCC 49175 BPR 1X MIC
5. *G. adiacens* ATCC 49175 AMP 1/2X MIC
6. *G. adiacens* ATCC 49175 AMP 1X MIC
7. *G. adiacens* ATCC 49175 CRO 1/2X MIC
8. *G. adiacens* ATCC 49175 CRO 1X MIC
9. *G. adiacens* ATCC 49175 BPR 1/2X MIC + AMP 1X MIC
10. *G. adiacens* ATCC 49175 BPR 1X MIC + AMP 1X MIC
11. *G. adiacens* ATCC 49175 CRO 1/2X MIC + AMP 1X MIC
12. *G. adiacens* ATCC 49175 CRO 1X MIC + AMP 1X MIC
13. Precision Plus Protein WesternC (BIORAD)
14. Ladder Precision Plus Protein Dual Color Standards (BIORAD)

(a) PBP/Bocillin-labelled profiles on SDS-PAGE gel for *G. adiacens* ATCC 49175
(b) Coomassie blue staining gel corresponding to PBP/Bocillin-labelled profiles on SDS-PAGE gel for *G. adiacens* ATCC 49175
