# Supplementary material for: Target Fidelity and Failure: Structure–Activity Relationship of High-Molecular-Mass Penicillin-Binding Proteins (HMM-PBPs) in Refractory Granulicatella adiacens Endocarditis
Source: Antibiotics (Basel). 2026 Feb 5;15(2):168. doi: 10.3390/antibiotics15020168 (PMC12937247; doi:10.3390/antibiotics15020168)
Supplement: Supplementary file 1 [file antibiotics-15-00168-s001.zip › Supplementary files/Figure S1. PBPs amino acid sequences multi alignment via clustal omega.aln-clustal_num.pdf]

CLUSTAL O(1.2.4) multiple sequence alignment

```
G.adiacensATCC49175_PBP1B         ---------------------------------------------------MSVQNQTPD 9
G.adiacensGA01_PBP1B              ---------------------------------------------------MSVQNQTPD 9
G.adiacensIS48_PBP1B              ---------------------------------------------------MSVQNQTPD 9
G.adiacensKHUD009_PBP1B           ---------------------------------------------------MSVQNQTPD 9
S.pneumoniaeD39_PBP1B             -----------------------------------------------------MQNQLNE 7
S.pneumoniaeR6_PBP1B              -----------------------------------------------------MQNQLNE 7
S.pneumoniaeNCTC7465_PBP1B        -----------------------------------------------------MQNQLNE 7
S.pyogenesATCC-BAA-595_PBP1B      ------------------------------------------------------------ 0
E.faeciumDO_PBP1B                 ------------------------------------------------------------ 0
E.faecalisOG1RF_PBP1B             ------------------------------------------------------------ 0
G.adiacensATCC49175_PBP2A         ---------------------------------MNEEFNEKDYVDHVT------HNQEQE 21
G.adiacensKHUD009_PBP2A           ---------------------------------MNEEFNEKDYVDHVT------HNQEQE 21
G.adiacensGA01_PBP2A              ---------------------------------MNEEFNEKDHVDHVT------HDQEQE 21
G.adiacensIS48_PBP2A              ---------------------------------MNEEFNEKDHVDHVT------HDQEQE 21
E.faecalisOG1RF_PBP2A             ------------------------------------------------------------ 0
E.faeciumDO_PBP2A                 ------------------------------------------------------------ 0
S.pyogenesATCC-BAA-595_PBP2A      ---------------------MRFLE-LLQKKFFPKAYQEKQFLMHQKARLTPQHNQKQY 38
S.pneumoniaeR6_PBP2A              ---------------------MKLDK-LF-EKFLS-----------------------LF 14
S.pneumoniaeD39_PBP2A             ---------------------MKLDK-LF-EKFLS-----------------------LF 14
S.pneumoniaeNCTC7465_PBP2A        ---------------------MKLDK-LF-EKFLS-----------------------LF 14
G.adiacensKHUD009_PBP1A           ---------------------------------------------------MTENNTGQS 9
G.adiacensIS48_PBP1A              ---------------------------------------------------MTENNTGQS 9
G.adiacensATCC49175_PBP1A         ---------------------------------------------------MTENNTGQS 9
G.adiacensGA01_PBP1A              ---------------------------------------------------MTENNTGQS 9
E.faecalisOG1RF_PBP1A             ---------------------------------------------------MPTANSGSR 9
E.faeciumDO_PBP1A                 -----------------------------------------------------MANEQTR 7
S.pyogenes_ATCC-BAA-595_PBP1A     ------------------------------------------------------------ 0
S.pneumoniaeNCTC7465_PBP1A        ------------------------------------------------------------ 0
S.pneumoniaeR6_PBP1A              ------------------------------------------------------------ 0
S.pneumoniaeD39_PBP1A             ------------------------------------------------------------ 0
G.adiacensIS48_PBP2B              ------------------------------------------------------------ 0
G.adiacensGA01_PBP2B              ------------------------------------------------------------ 0
G.adiacensATCC49175_PBP2B         ------------------------------------------------------------ 0
G.adiacensKHUD009_PBP2B           ------------------------------------------------------------ 0
S.pneumoniaeR6_PBP2B              ------------------------------------------------------------ 0
S.pneumoniaeD39_PBP2B             ------------------------------------------------------------ 0
S.pneumoniaeNCTC7465_PBP2B        ------------------------------------------------------------ 0
E.faecalisOG1RF_PBP2B             ------------------------------------------------------------ 0
E.faeciumDO_PBP2B                 ------------------------------------------------------------ 0
E.faecalisOG1RF_PBP4              MERSNRNKKSSKKPLILGVSALVLIAAAGGGYYAYSQWQAKQELAEAK------------ 48
```

```
E.faeciumDO_PBP5                   MKRSDKHGKNRTGAYI--AGAVILIAAAGGGYFYYQHYQETQAVEAGE------------ 46
S.pyogenesATCC-BAA-595_PBP2        ----------------------------------MKKWQK-------------------- 6
S.pneumoniaeR6_PBP2                -----------------------------------MKWTK-------------------- 5
S.pneumoniaeD39_PBP2               -----------------------------------MKWTK-------------------- 5
S.pneumoniaeNCTC7465_PBP2          -----------------------------------MKWTK-------------------- 5
G.adiacensATCC49175_PBP2           ------------------------------------------------------------ 0
G.adiacensIS48_PBP2                ------------------------------------------------------------ 0
G.adiacensGA01_PBP2                ------------------------------------------------------------ 0
G.adiacensKHUD009_PBP2             ------------------------------------------------------------ 0
E.faecalisOG1RF_PBP2               -----------------------------------MSKRH-------------------- 5
E.faeciumDO_PBP2                   -----------------------------------MSLKN-------------------- 5


G.adiacensATCC49175_PBP1B          -----------------------------WREALLTFWNKPI---------------L-- 23
G.adiacensGA01_PBP1B               -----------------------------WREALLTFWNKPI---------------L-- 23
G.adiacensIS48_PBP1B               -----------------------------WREALLTFWNKPI---------------L-- 23
G.adiacensKHUD009_PBP1B            -----------------------------WREALLTFWNKPI---------------L-- 23
S.pneumoniaeD39_PBP1B              -----------------------------LKRKMLEFFQQKQ---------------KNK 23
S.pneumoniaeR6_PBP1B               -----------------------------LKRKMLEFFQQKQ---------------KNK 23
S.pneumoniaeNCTC7465_PBP1B         -----------------------------LKRKMLEFFQQKQ---------------KNK 23
S.pyogenesATCC-BAA-595_PBP1B       ---------------------------------MVKW-NTKQ---------------KRI 11
E.faeciumDO_PBP1B                  ---------------------------------------MTL---------------LPR 6
E.faecalisOG1RF_PBP1B              ---------------------------------MYHFIEVKL---------------LKN 12
G.adiacensATCC49175_PBP2A          ET-------VEHRHTEEPTQQSIIK------------RVWN------------------- 43
G.adiacensKHUD009_PBP2A            ET-------VEHRHTEEPTQQSIIK------------RVWN------------------- 43
G.adiacensGA01_PBP2A               ET-------VEHRHTEEPTQPSIIK------------RVWN------------------- 43
G.adiacensIS48_PBP2A               ET-------VEHRHTEEPTQPSIIK------------RVWN------------------- 43
E.faecalisOG1RF_PBP2A              --------------MD--NLKQFFSKVG---VGLRHFWTWIK------------------ 23
E.faeciumDO_PBP2A                  ---------------M--DFQSILEKIK---AALISFWRWIR------------------ 22
S.pyogenesATCC-BAA-595_PBP2A       SPNAHHLDSSATKNSE-QDPAIALQRSRAYEGSPKSRPAWLQ------------------ 79
S.pneumoniaeR6_PBP2A               KK--------ETSELE-DSDSTILRRSR-------------------------------- 33
S.pneumoniaeD39_PBP2A              KK--------ETSELE-DSDSTILRRSR-------------------------------- 33
S.pneumoniaeNCTC7465_PBP2A         KK--------ETSELE-DSDSTILRRSR-------------------------------- 33
G.adiacensKHUD009_PBP1A            R----------SSQHRQ------------------------------------------- 16
G.adiacensIS48_PBP1A               R----------SSQHRQ------------------------------------------- 16
G.adiacensATCC49175_PBP1A          R----------SSQHRQ------------------------------------------- 16
G.adiacensGA01_PBP1A               R----------SSQHRQ------------------------------------------- 16
E.faecalisOG1RF_PBP1A              A----------ARHASNTSSK----------------SSYK------------------- 24
E.faeciumDO_PBP1A                  S----------SRRQKQPTPK----------------KSVKK------------------ 23
S.pyogenes_ATCC-BAA-595_PBP1A      ------------------------------------------------------------ 0
S.pneumoniaeNCTC7465_PBP1A         ------------------------------------------------------------ 0
S.pneumoniaeR6_PBP1A               ------------------------------------------------------------ 0
S.pneumoniaeD39_PBP1A              ------------------------------------------------------------ 0
```

```
G.adiacensIS48_PBP2B              ---------------------------------------------ML------------- 2
G.adiacensGA01_PBP2B              ---------------------------------------------ML------------- 2
G.adiacensATCC49175_PBP2B         ---------------------------------------------ML------------- 2
G.adiacensKHUD009_PBP2B           ---------------------------------------------ML------------- 2
S.pneumoniaeR6_PBP2B              ------------------------------------------------------------ 0
S.pneumoniaeD39_PBP2B             ------------------------------------------------------------ 0
S.pneumoniaeNCTC7465_PBP2B        ------------------------------------------------------------ 0
E.faecalisOG1RF_PBP2B             ----------------------------------------MKKNSFID------------ 8
E.faeciumDO_PBP2B                 --------------------------------MMKDFMKKILNTSWLE------------ 16
E.faecalisOG1RF_PBP4              -------------------------------KTATTFLNVLSKQEFDKLPSVVQEASLKK 77
E.faeciumDO_PBP5                  -------------------------------KTVEQFVQALNKGDYNKAAEMTSKKAANK 75
S.pyogenesATCC-BAA-595_PBP2       --------------------------------YVLDYVVR-------------------- 14
S.pneumoniaeR6_PBP2               --------------------------------RVIRYATK-------------------- 13
S.pneumoniaeD39_PBP2              --------------------------------RVIRYATK-------------------- 13
S.pneumoniaeNCTC7465_PBP2         --------------------------------RVIRYATK-------------------- 13
G.adiacensATCC49175_PBP2          ------------------------------------MKKT-------------------- 4
G.adiacensIS48_PBP2               ------------------------------------MKKT-------------------- 4
G.adiacensGA01_PBP2               ------------------------------------MKKT-------------------- 4
G.adiacensKHUD009_PBP2            ------------------------------------MKKT-------------------- 4
E.faecalisOG1RF_PBP2              --------------------------------KFKQFMKK-------------------- 13
E.faeciumDO_PBP2                  --------------------------------KIRRFMEK-------------------- 13


G.adiacensATCC49175_PBP1B         ----------------------KNIRFIFNIAYSVLKNIFVIAILFLMLLGIFGGGIGLG 61
G.adiacensGA01_PBP1B              ----------------------KNIRFIFNIAYSVLKNIFVIAILFLMLLGIFGGGIGLG 61
G.adiacensIS48_PBP1B              ----------------------KNIRFIFNIAYSVLKNIFVIAILFLMLLGIFGGGIGLG 61
G.adiacensKHUD009_PBP1B           ----------------------KNIRFIFNIAYSVLKNIFVIAILFLMLLGIFGGGIGLG 61
S.pneumoniaeD39_PBP1B             KS-----ARPGKKGSSTKKSKTLDKSAIFPAILLSIKALFNLLFVLGFLGGMLGAGIALG 78
S.pneumoniaeR6_PBP1B              KS-----ARPGKKGSSTKKSKTLDKSAIFPAILLSIKALFNLLFVLGFLGGMLGAGIALG 78
S.pneumoniaeNCTC7465_PBP1B        KS-----ARPGKKGSSTKKSKTLDKSAIFPAILLSIKALFNLLFVLGFLGGMLGVGIALG 78
S.pyogenesATCC-BAA-595_PBP1B      ------------------SHQRLGLLDLGPVLLRTLRLLSNFFYIVIFLFGMMGFGMAFG 53
E.faeciumDO_PBP1B                 KN-----TR--KKQ----NRRKKEKWFVPKIIFRVFQSLTVFITVLLLMFAALGIGIGAG 55
E.faecalisOG1RF_PBP1B             NS-----SN--NKQKPTTSSGGNVFLLILNVIIRVFQSLVVFGVILIVLGGSLGLGIGMG 65
G.adiacensATCC49175_PBP2A         ------------------------HKIMLAIRRFWRKFHVTKLILVLMLTAITAFSA--- 76
G.adiacensKHUD009_PBP2A           ------------------------HKIMLAIRRFWRKFHVTKLILVLMLTAITAFSA--- 76
G.adiacensGA01_PBP2A              ------------------------HKIMLAIRRFWRKFHVTKLILVLILTAITAFSA--- 76
G.adiacensIS48_PBP2A              ------------------------HKIMLAIRRFWRKFHVTKLILVLILTAITAFSA--- 76
E.faecalisOG1RF_PBP2A             ---------------------PYLIQFHQARKRIWKKYQINKIFLLIGLVVALGASI--- 59
E.faeciumDO_PBP2A                 ---------------------PYLGQFHRWRKRIWKKYHVNKLILLIGLICVLVTSI--- 58
S.pyogenesATCC-BAA-595_PBP2A      ------------------KLEAVLPSPQHPIRRFWRRYHIGKLLMILIGTLVLLLGS--- 118
S.pneumoniaeR6_PBP2A              -------------------SDRKKLAQVGPIRKFWRRYHLTKIILILGLSAGLLVGI--- 71
S.pneumoniaeD39_PBP2A             -------------------SDRKKLAQVGPIRKFWRRYHLTKIILILGLSAGLLVGI--- 71
S.pneumoniaeNCTC7465_PBP2A        -------------------SDRKKLAQVGPIRKFWRRYHLTKIILILGLSAGLLVGI--- 71
```

```
G.adiacensKHUD009_PBP1A          ---------------------PKKKASSPSNKKKILKKVLIGLGAFIGVALIAIIAI--- 52
G.adiacensIS48_PBP1A             ---------------------PKKKASSPSNKKKILKKVLIGLGAFIGVALIAIIAI--- 52
G.adiacensATCC49175_PBP1A        ---------------------PKKKASSPSNKKKILKKVLIGLGAFIGVALISIIAI--- 52
G.adiacensGA01_PBP1A             ---------------------PKKKASSPSNKKKILKKVLIGLGAFIGVALIAIIAI--- 52
E.faecalisOG1RF_PBP1A            -----------------------KKTQPPKKKKRLVLKIFLGLLIAGMVAFLAGVGL--- 58
E.faeciumDO_PBP1A                ------------------NSGKGSGKSSGTHKKGLFIKILLGILSFFCILFLAGVGL--- 62
S.pyogenes_ATCC-BAA-595_PBP1A    -----------------------MITIKNPKILKWLKYVLSAILSLIILIIIIGGLL--- 34
S.pneumoniaeNCTC7465_PBP1A       --------------------------MNKPTILRLIKYLSISFLSLVIAAIVLGGGV--- 31
S.pneumoniaeR6_PBP1A             --------------------------MNKPTILRLIKYLSISFLSLVIAAIVLGGGV--- 31
S.pneumoniaeD39_PBP1A            --------------------------MNKPTILRLIKYLSISFLSLVIAAIVLGGGV--- 31
G.adiacensIS48_PBP2B             ------------NKRKS---KNKKKKSHIPFRLN----LLFLIVFFSFIALISRLAY--- 40
G.adiacensGA01_PBP2B             ------------NKRKS---KNKKKKSHIPFRLN----LLFLIVFFSFIALISRLAY--- 40
G.adiacensATCC49175_PBP2B        ------------NKRKS---KNKKKKSHIPFRLN----LLFLIVFFSFIALISRLAY--- 40
G.adiacensKHUD009_PBP2B          ------------NKRKS---KNKKKKSHIPFRLN----LLFLIVFFSFIALISRLAY--- 40
S.pneumoniaeR6_PBP2B             ------------MRLIC---MRKFNSHSIPIRLN----LLFSIVILLFMTIIGRLLY--- 38
S.pneumoniaeD39_PBP2B            ------------MRLIC---MRKFNSHSIPIRLN----LLFSIVILLFMTIIGRLLY--- 38
S.pneumoniaeNCTC7465_PBP2B       --------------------MRKFNSHSIPIRLN----LLFSIVILLFMTIIGRLLY--- 33
E.faecalisOG1RF_PBP2B            ------KIKK-TDAAAK---SQRGRKPHIPFRLN----LLFFVIFTLFVSLIVRLGY--- 51
E.faeciumDO_PBP2B                ------KIRK-GAQEPK---NKTFRRSHVPFRLN----FLFFIIFTLFVALIARLGY--- 59
E.faecalisOG1RF_PBP4             NGYDTKSVVE-KYQAIY---SGIQAEGVKASDVQVKKAKDNQYTFTYKLSMSTPLGE--- 130
E.faeciumDO_PBP5                 SALSEKEILD-KYQNIY---GAADVKGLQISNLKVDKKDDSTYSFSYKAKMNTSLGE--- 128
S.pyogenesATCC-BAA-595_PBP2       DR-----------RTPV---ENRVRVGQNMMLLT----IFIFFIFIINFMIIIGTDQ--- 53
S.pneumoniaeR6_PBP2              NR-----------KSPA---ENRRRVGKSLSLLS----VFVFAIFLVNFAVIIGTGT--- 52
S.pneumoniaeD39_PBP2             NR-----------KSPA---ENRRRVGKSLSLLS----VFVFAIFLVNFAVIIGTGT--- 52
S.pneumoniaeNCTC7465_PBP2        NR-----------KSPA---ENRRRVGKSLSLLS----VFVFAIFLVNFAVIIGTGT--- 52
G.adiacensATCC49175_PBP2         NN-----------NNIA---RNRRRASFILMGLA----SVLFLIFSVRFFRIMVLGN--- 43
G.adiacensIS48_PBP2              NN-----------NNIA---RNRRRASFILLGLA----SVLFLIFSVRFFRIMVLGN--- 43
G.adiacensGA01_PBP2              NN-----------NNIA---RNRRRASFILLGLA----SVLFLIFSVRFFRIMVLGN--- 43
G.adiacensKHUD009_PBP2           NN-----------NNIA---RNRRRASFIIMGLA----SVLFLIFSVRFFRIMVLGN--- 43
E.faecalisOG1RF_PBP2             KN-----------LNPM---NNRKKVGIILFATS----IGLFFLFAFRTTYIVATGK--- 52
E.faeciumDO_PBP2                 KN-----------LNPM---NNRKKVGIILFATS----IGLFFLFAVRFSYIVIGGH--- 52


G.adiacensATCC49175_PBP1B        YFASLTSNEEP-------------------LTQEQMSDAIGNLNLISSFHYNDGTKISDV 102
G.adiacensGA01_PBP1B             YFASLTSNEEP-------------------LTQEQMSDAIGNLNLISSFHYSDGTKISDV 102
G.adiacensIS48_PBP1B             YFASLTSNEEP-------------------LTQEQMSDAIGNLNLISSFHYSDGTKISDV 102
G.adiacensKHUD009_PBP1B          YFASLTSNEEP-------------------LTQEQMSDAIGNLNLISSFHYSDGTKISDV 102
S.pneumoniaeD39_PBP1B            YGVALFDKVRV-------------------PQTEELVNQVKDISSISEITYSDGTVIASI 119
S.pneumoniaeR6_PBP1B             YGVALFDKVRV-------------------PQTEELVNQVKDISSISEITYSDGTVIASI 119
S.pneumoniaeNCTC7465_PBP1B       YGVALFDKVRV-------------------PQTEELVNQVKDISSISEITYSDGTVIASI 119
S.pyogenesATCC-BAA-595_PBP1B      YLASQIESVKV-------------------PSKESLVKQVESLTMISQMNYSDNSLISTL 94
E.faeciumDO_PBP1B                YFAYLVEDTQL-------------------PTKKALQTELGNITETSKIVYADNTEISKI 96
E.faecalisOG1RF_PBP1B            YFAFLVEDTQP-------------------PTKEELQKEISDITEVSKMTYADGTPIANI 106
```

```
G.adiacensATCC49175_PBP2A        -FLVYT--AKT-------------------TDVSGLRAG----MVQVTEVYDRNNQEAGV 110
G.adiacensKHUD009_PBP2A          -FLVYT--AKT-------------------TDVSGLRAG----MVQVTEVYDRNNQEAGV 110
G.adiacensGA01_PBP2A             -FLVYT--AKT-------------------TDVSGLRAG----MVQVTEVYDRNNQEAGV 110
G.adiacensIS48_PBP2A             -FLVYT--AKT-------------------TDVSGLRAG----MVQVTEVYDRNNQEAGV 110
E.faecalisOG1RF_PBP2A            -YLFYL--AKS-------------------ANVETLKSG----LSESTRVYDESGEEVGK 93
E.faeciumDO_PBP2A                -YLFIL--AKQ-------------------ANVETLKSG----LSQSTVIYDKKNEEAGT 92
S.pyogenesATCC-BAA-595_PBP2A     -YLFYL--SKT-------------------AKVSDLQDA----LKATTVIYDHKGEYAGS 152
S.pneumoniaeR6_PBP2A             -YLFAV--AKS-------------------TNVNDLQNA----LKTRTLIFDREEKEAGA 105
S.pneumoniaeD39_PBP2A            -YLFAV--AKS-------------------TNVNDLQNA----LKTRTLIFDREEKEAGA 105
S.pneumoniaeNCTC7465_PBP2A       -YLFAV--AKS-------------------TNVNDLQNA----LKTRTLIFDREEKEAGA 105
G.adiacensKHUD009_PBP1A          -FAYYG--STA-------------------PEIKVSDLQ----GATETKIYDKDGELISS 86
G.adiacensIS48_PBP1A             -FAYYG--STA-------------------PEIKASDLQ----GATETKIYDKDGELISS 86
G.adiacensATCC49175_PBP1A        -FAYYG--STA-------------------PEIKASDLQ----GATETKIYDKDGELISS 86
G.adiacensGA01_PBP1A             -FAYYG--STA-------------------PEIKASDLQ----GATETKIYDKDGELISS 86
E.faecalisOG1RF_PBP1A            -FWFYA--RQA-------------------PKLEDDKLN----ATVSSKLYDINNEIFED 92
E.faeciumDO_PBP1A                -FWYYA--KDA-------------------PELTDKKLD----ATVSSKLYTQDGELFED 96
S.pyogenes_ATCC-BAA-595_PBP1A    -FTFYI--SSA-------------------PKLSEAQLK----STNSSLVYDGNNNLIAD 68
S.pneumoniaeNCTC7465_PBP1A       -FFYYV--SKA-------------------PSLSESKLV----ATTSSKIYDNKNQLIAD 65
S.pneumoniaeR6_PBP1A             -FFYYV--SKA-------------------PSLSESKLV----ATTSSKIYDNKNQLIAD 65
S.pneumoniaeD39_PBP1A            -FFYYV--SKA-------------------PSLSESKLV----ATTSSKIYDNKNQLIAD 65
G.adiacensIS48_PBP2B             -V-QLVKGDE------------------FVALVQRTETTTSKKAVPRGSIYDSQGRVLVG 80
G.adiacensGA01_PBP2B             -V-QLVKGDE------------------FVALVQRTETTTSKKAVPRGSIYDSQGRVLVG 80
G.adiacensATCC49175_PBP2B        -V-QLVKGDE------------------FVALVQRTETTTSKKAVPRGSIYDSQGRVLVG 80
G.adiacensKHUD009_PBP2B          -V-QLVKGDE------------------FVALVQRTETTTSKKAVPRGSIYDSQGRVLVG 80
S.pneumoniaeR6_PBP2B             -M-QVLNKDF------------------YEKKLASASQTKITSSSARGEIYDASGKPLVE 78
S.pneumoniaeD39_PBP2B            -M-QVLNKDF------------------YEKKLASASQTKITSSSARGEIYDASGKPLVE 78
S.pneumoniaeNCTC7465_PBP2B       -M-QVLNKDF------------------YEKKLASASQTKITSSSARGEIYDASGKPLVE 73
E.faecalisOG1RF_PBP2B            -L-QIVEGEE------------------FNKKITANSSLQITTPSPRGQIYDSQGKVLVS 91
E.faeciumDO_PBP2B                -L-QIVNGEE------------------MEARVKSTSTITVQESTPRGMIYDSTGKALVT 99
E.faecalisOG1RF_PBP4             -M-KDLSYQSSIAKKGDTYQIAWKPSLIFPDMSGNDKISIQVDNAKRGEIVDRNGSGLAI 188
E.faeciumDO_PBP5                 -L-KDLSYKGTLDRNDGQTTINWQPNLVFPEMEGNDKVSLTTQEAARGNIIDRNGEPLAT 186
S.pyogenesATCC-BAA-595_PBP2       -K-FGVSLSE---------------------GAKKVYQETVTIQAKRGTIYDRNGTAIAV 90
S.pneumoniaeR6_PBP2              -R-FGTDLAK---------------------EAKKVHQTTRTVPAKRGTIYDRNGVPIAE 89
S.pneumoniaeD39_PBP2             -R-FGTDLAK---------------------EAKKVHQTTRTVPAKRGTIYDRNGVPIAE 89
S.pneumoniaeNCTC7465_PBP2        -R-FGTDLAK---------------------EAKKVHQTTRTVPAKRGTIYDRNGVPIAE 89
G.adiacensATCC49175_PBP2         -I-HNVDLRA---------------------EINDKIHQKRTLAAKRGTIYDASGSPIAV 80
G.adiacensIS48_PBP2              -I-HNVDLRA---------------------EINDKIHQKRTLAAKRGTIYDASGSPIAV 80
G.adiacensGA01_PBP2              -I-HNVDLRA---------------------EINDKIHQKRTLAAKRGTIYDASGSPIAV 80
G.adiacensKHUD009_PBP2           -I-HNVDLRA---------------------EINDKIHQKRTLAAKRGTIYDASGSPIAV 80
E.faecalisOG1RF_PBP2             -V-AGVSLKE---------------------KTASLYEGSQVVKAKRGSILDRYGNPIAE 89
E.faeciumDO_PBP2                 -V-AGTSLAE---------------------KTKQLYQGSEVVKAKRGTIYDRNGVALAE 89
```

```
G.adiacensATCC49175_PBP1B         NSDELRIVKPLSEISQYVIDGIISTEDSSFY-EHNGIVPKALLRAILQEAVSSGS-GATG 160
G.adiacensGA01_PBP1B              NSDELRIVKPLSEISQYVIDGIISTEDSSFY-EHNGIVPKALLRAILQEAVSSGS-GATG 160
G.adiacensIS48_PBP1B              NSDELRIVKPLSEISQYVIDGIISTEDSSFY-EHNGIVPKALLRAILQEAVSSGS-GATG 160
G.adiacensKHUD009_PBP1B           NSDELRIVKPLSEISQYVIDGIISTEDSSFY-EHNGIVPKALLRAILQEAVSSGS-GATG 160
S.pneumoniaeD39_PBP1B             ESDLLRTSISSEQISENLKKAIIATEDEHFK-EHKGVVPKAVIRATLGKFVGLG--SSSG 176
S.pneumoniaeR6_PBP1B              ESDLLRTSISSEQISENLKKAIIATEDEHFK-EHKGVVPKAVIRATLGKFVGLG--SSSG 176
S.pneumoniaeNCTC7465_PBP1B        ESDLLRTSISSEQISENLKKAIIATEDEHFK-EHKGVVPKAVIRATLGKFVGLG--SSSG 176
S.pyogenesATCC-BAA-595_PBP1B      DTDLLRTPVANDAISENIKKAIVSTEDEHFQ-EHKGVVPKAVFRATLASVLGFG--EASG 151
E.faeciumDO_PBP1B                 QTDLMRTTISSDKISPLLKTAIISTEDEYFD-KHQGYVPKAVLRALVSEATGIG---SSG 152
E.faecalisOG1RF_PBP1B             KSDLIRTRINGDQMSPLLKKAIISTEDEYFE-EHHGVVPKALVRALISDATGIG--GSSG 163
G.adiacensATCC49175_PBP2A         LNINKGEFVTIDQISPNMINALVSTEDKRFY-DHHGFDPMGFLRATFGLLLNRGR-VTGG 168
G.adiacensKHUD009_PBP2A           LNINKGEFVTIDQISPNMINALVSTEDKRFY-DHHGFDPMGFLRATFGLLLNRGR-VTGG 168
G.adiacensGA01_PBP2A              LNINKGEFVTIDQISPNMINALVSTEDKRFY-DHHGFDPMGFLRATFGLLLNRGR-VTGG 168
G.adiacensIS48_PBP2A              LNINKGEFVTIDQISPNMINALVSTEDKRFY-DHHGFDPMGFLRATFGLLLNRGR-VTGG 168
E.faecalisOG1RF_PBP2A             LFGQKGTFVELDNISPYIQDAVISTEDRGFY-QHKGYSIKGIARAVVGKLTFGKIGGGGG 152
E.faeciumDO_PBP2A                 LYAQKGTYVELNAISPYIQDAVISTEDRNFY-EHHGFDIKGIARAAVRMVLSGGT-SGGG 150
S.pyogenesATCC-BAA-595_PBP2A      LSGQKGSYVELNAISDDLENAVIATEDRTFY-SNSGINLKRFLLAVVT------AGRFGG 205
S.pneumoniaeR6_PBP2A              LSGQKGTYVELTDISKNLQNAVIATEDRSFY-KNDGINYGRFFLAIVT------AGRSGG 158
S.pneumoniaeD39_PBP2A             LSGQKGTYVELTDISKNLQNAVIATEDRSFY-KNDGINYGRFFLAIVT------AGRSGG 158
S.pneumoniaeNCTC7465_PBP2A        LSGQKGTYVELTDISKNLQNAVIATEDRSFY-KNDGINYGRFFLAIVT------AGRSGG 158
G.adiacensKHUD009_PBP1A           LGGEKRDVITSDQVPQLLKDAVTSIEDKRFY-SHMGIDPIRILGSFFRNAKAGQI--TQG 143
G.adiacensIS48_PBP1A              LGGEKRDVITSDQVPQLLKDAVTSIEDKRFY-SHMGIDPIRILGSFFRNAKAGQI--TQG 143
G.adiacensATCC49175_PBP1A         LGGEKRDVITSDQVPQLLKDAVTSIEDKRFY-SHMGIDPIRILGSFFRNAKAGQI--TQG 143
G.adiacensGA01_PBP1A              LGGEKRDVITSDQVPQLLKDAVTSIEDKRFY-SHMGIDPIRILGSFFRNAKAGQI--TQG 143
E.faecalisOG1RF_PBP1A             LGAEKRELIQPNDVPQLLKDAIVSVEDRRFY-KHIGVDPIRIIGSALSNVKNGG---LQG 148
E.faeciumDO_PBP1A                 LGAEKREKISANELPKTLEDAIVSVEDRRFY-KHIGVDPIRIIGSALSNFTSGG---LQG 152
S.pyogenes_ATCC-BAA-595_PBP1A     LGSEKRENVTADSIPINLVNAITSIEDKRFF-NHRGVDLYRIFGAAFHNLTSQT---TQG 124
S.pneumoniaeNCTC7465_PBP1A        LGSERRVNAQANDIPTDLVKAIVSIEDHRFF-DHRGIDTIRILGAFLRNLQSNS---LQG 121
S.pneumoniaeR6_PBP1A              LGSERRVNAQANDIPTDLVKAIVSIEDHRFF-DHRGIDTIRILGAFLRNLQSNS---LQG 121
S.pneumoniaeD39_PBP1A             LGSERRVNAQANDIPTDLVKAIVSIEDHRFF-DHRGIDTIRILGAFLRNLQSNS---LQG 121
G.adiacensIS48_PBP2B              NKPKLSINYTRPA-------D----------AKASKMLEIAK--K-L------------- 107
G.adiacensGA01_PBP2B              NKPKLAINYTRPA-------D----------AKSSTMLEIAK--K-L------------- 107
G.adiacensATCC49175_PBP2B         NKPKLSINYTRPA-------D----------AKASKMLEIAK--K-L------------- 107
G.adiacensKHUD009_PBP2B           NKPKLSINYTRPA-------D----------AKASKMLEIAK--K-L------------- 107
S.pneumoniaeR6_PBP2B              NTLKQVVSFTRSN-------K----------MTATDLKETAK--K-L------------- 105
S.pneumoniaeD39_PBP2B             NTLKQVVSFTRSN-------K----------MTATDLKETAK--K-L------------- 105
S.pneumoniaeNCTC7465_PBP2B        NTLKQVVSFTRSN-------K----------MTATDLKETAK--K-L------------- 100
E.faecalisOG1RF_PBP2B             NKANLAITYTRGK-------N----------IEGKDILPIAN--K-V------------- 118
E.faeciumDO_PBP2B                 NKANQAITYTRGT-------Q----------VTAKDLLEIAT--K-L------------- 126
E.faecalisOG1RF_PBP4              NKVFDEVGVVPGK-------LGSGA-EKTAN---------IK--A-F------------- 215
E.faeciumDO_PBP5                  TGKLKQLGVVPSK-------LGDGG-EKTAN---------IK--A-I------------- 213
S.pyogenesATCC-BAA-595_PBP2       DSTTYSIYAILDK-------SFVSASDEKLYVQPSQYETVAD--I-L------------- 127
S.pneumoniaeR6_PBP2               DATSYNVYAVIDE-------NYKSATGKILYVEKTQFNKVAE--V-F------------- 126
```

```
S.pneumoniaeD39_PBP2              DATSYNVYAVIDE-------NYKSATGKILYVEKTQFNKVAE--V-F------------- 126
S.pneumoniaeNCTC7465_PBP2         DATSYNVYAVIDE-------NYKSATGKILYVEKTQFNKVAE--V-F------------- 126
G.adiacensATCC49175_PBP2          DATNYSIYAVLTN-------QWSKNAETPDYVT--DINKTAE--A-L------------- 115
G.adiacensIS48_PBP2               DATNYSIYAVLTD-------QWSKNAETPDYVT--DINKTAE--A-L------------- 115
G.adiacensGA01_PBP2               DATNYSIYAVLTD-------QWSKNAETPDYVT--DINKTAE--A-L------------- 115
G.adiacensKHUD009_PBP2            DATNYSIYAVLTD-------QWSKNAETPDYVT--DINKTAE--A-L------------- 115
E.faecalisOG1RF_PBP2              DATSYSLYVVLSK-------KYTGQNNEKLYAEKKDFDDIAE--I-L------------- 126
E.faeciumDO_PBP2                  DASSYSIKAILSK-------TYTSG-DKKLYVEEKNFDKIAE--I-L------------- 125
                                                                             .

G.adiacensATCC49175_PBP1B         GSTLTQQLIKQQILT---SEVTFKRKANEILYALRLEKHFTKDQILEAYL---------- 207
G.adiacensGA01_PBP1B              GSTLTQQLIKQQILT---SEVTFKRKANEILYALRLEKHFTKDQILEAYL---------- 207
G.adiacensIS48_PBP1B              GSTLTQQLIKQQILT---SEVTFKRKANEILYALRLEKHFTKDQILEAYL---------- 207
G.adiacensKHUD009_PBP1B           GSTLTQQLIKQQILT---SEVTFKRKANEILYALRLEKHFTKDQILEAYL---------- 207
S.pneumoniaeD39_PBP1B             GSTLTQQLIKQQVVG---DAPTLARKAAEIVDALALERAMNKDEILTTYL---------- 223
S.pneumoniaeR6_PBP1B              GSTLTQQLIKQQVVG---DAPTLARKAAEIVDALALERAMNKDEILTTYL---------- 223
S.pneumoniaeNCTC7465_PBP1B        GSTLTQQLIKQQVVG---DAPTLARKAAEIVDALALERAMNKDEILTTYL---------- 223
S.pyogenesATCC-BAA-595_PBP1B      GSTLTQQLVKQQVLG---DDPTFKRKSKEIVYALALERYMSKDNILCDYL---------- 198
E.faeciumDO_PBP1B                 GSTLTQQLVKQQILT---DETTFKRKANEIILSAQVEKYFSKDEIIATYL---------- 199
E.faecalisOG1RF_PBP1B             GSTLTQQLVKQQILT---DETTFKRKANEILLALRIEKYFSKDEIVTTYL---------- 210
G.adiacensATCC49175_PBP2A         GSTITQQLAKNAFLT---QDQTFLRKAKELFLSFELEKKYSKDQILEMYL---------- 215
G.adiacensKHUD009_PBP2A           GSTITQQLAKNAFLT---QDQTFLRKAKELFLSFELEKKYSKDQILEMYL---------- 215
G.adiacensGA01_PBP2A              GSTITQQLAKNAFLT---QDQTFLRKAKELFLSFELEKKYSKDQILEMYL---------- 215
G.adiacensIS48_PBP2A              GSTITQQLAKNAFLT---QDQTFLRKAKELFLSFELEKKYSKDQILEMYL---------- 215
E.faecalisOG1RF_PBP2A             GSTITQQLAKNAYLT---QEQTLDRKARELFLAIEIEKKYSKKDILAMYL---------- 199
E.faeciumDO_PBP2A                 GSTITQQLAKNAYLT---LDQTFDRKAKELFLAIEIEKKYSKEEILTMYL---------- 197
S.pyogenesATCC-BAA-595_PBP2A      GSTITQQLAKNAYLS---QDQTIKRKAREFFLALELTKKYSKKDILTMYL---------- 252
S.pneumoniaeR6_PBP2A              GSTITQQLAKNAYLS---QDQTVERKAKEFFLALELSKKYSKEQILTMYL---------- 205
S.pneumoniaeD39_PBP2A             GSTITQQLAKNAYLS---QDQTVERKAKEFFLALELSKKYSKEQILTMYL---------- 205
S.pneumoniaeNCTC7465_PBP2A        GSTITQQLAKNAYLS---QDQTVERKAKEFFLALELSKKYSKEQILTMYL---------- 205
G.adiacensKHUD009_PBP1A           GSTITQQLIKLSVFSTKKEDQTYQRKIQEAILALKLEREFSKEQILTFYL---------- 193
G.adiacensIS48_PBP1A              GSTITQQLIKLSVFSTKKEDQTYQRKIQEAILALKLEREFSKEQILTFYL---------- 193
G.adiacensATCC49175_PBP1A         GSTITQQLIKLSVFSTKKEDQTYQRKIQEAILALKLEREFSKEQILTFYL---------- 193
G.adiacensGA01_PBP1A              GSTITQQLIKLSVFSTKKEDQTYQRKIQEAILALKLEREFSKEQILTFYL---------- 193
E.faecalisOG1RF_PBP1A             GSTLTQQLIKLSYFSTKESDQTLKRKAQEAWMAVRLEREKSKEEILTYYI---------- 198
E.faeciumDO_PBP1A                 GSTLTQQLIKLSFFSTSAEDQTLKRKAQEAWMAVRLEQKKSKQEILTYYV---------- 202
S.pyogenes_ATCC-BAA-595_PBP1A     GSTLDQQLIKLAYFSTNESDQTLKRKAQEVWLALQMERKYTKQEILTFYI---------- 174
S.pneumoniaeNCTC7465_PBP1A        GSTLTQQLIKLTYFSTSTSDQTISRKAQEAWLAIQLEQKATKQEILTYYI---------- 171
S.pneumoniaeR6_PBP1A              GSALTQQLIKLTYFSTSTSDQTISRKAQEAWLAIQLEQKATKQEILTYYI---------- 171
S.pneumoniaeD39_PBP1A             GSTLTQQLIKLTYFSTSTSDQTISRKAQEAWLAIQLEQKATKQEILTYYI---------- 171
G.adiacensIS48_PBP2B              ----------TSLISV-DTSELKERDLKDYWVALNPDKLDG---LLTAEEKKQISKENL- 152
G.adiacensGA01_PBP2B              ----------TSLISV-DTSELKERDLKDYWVALNPDKLDG---LLTAEEKKQISKENL- 152
G.adiacensATCC49175_PBP2B         ----------TSLISV-DTSELKERDLKDYWVALNPDKLDG---LLTAEEKKQISKENL- 152
```

```
G.adiacensKHUD009_PBP2B            ----------TSLISV-DTSELKERDLKDYWVALNPDKLDG---LLTAEEKKQISKENL- 152
S.pneumoniaeR6_PBP2B               ----------LTYVSI-SSPNLTERQLADYYLADP-EIYKKIVEALPSEKRLDSDGNRL- 152
S.pneumoniaeD39_PBP2B              ----------LTYVSI-SSPNLTERQLADYYLADP-EIYKKIVEALPSEKRLDSDGNRL- 152
S.pneumoniaeNCTC7465_PBP2B         ----------LTYVSI-SSPNLTERQLADYYLADP-EIYKKTVEALPSEKRLDSDGNRL- 147
E.faecalisOG1RF_PBP2B              ----------NELINVPVDPNLTDRDKKDYWLANP-ENLKAAQARLTDQDKEDEKGNKIT 167
E.faeciumDO_PBP2B                  ----------NSLIDVPVDENLTDRDKKDFWLADP-KHLKEATKRLSAKEKQL------- 168
E.faecalisOG1RF_PBP4               ----------SDKFGVSVDE--INQKLSQGWVQA-------------------------- 237
E.faeciumDO_PBP5                   ----------ASSFDLTEDA--INQAISQSWVQP-------------------------- 235
S.pyogenesATCC-BAA-595_PBP2        ----------KKHLGMKKTD--VIKQLKR------KGLFQV---------SFGPSGS--- 157
S.pneumoniaeR6_PBP2                ----------HKYLDMEESY--VREQLSQ------PNLKQV---------SFGAKGN--- 156
S.pneumoniaeD39_PBP2               ----------HKYLDMEESY--VREQLSQ------PNLKQV---------SFGAKGN--- 156
S.pneumoniaeNCTC7465_PBP2          ----------HKYLDMEESY--VREQLSQ------PNLKQV---------SFGAKGN--- 156
G.adiacensATCC49175_PBP2           ----------SKHISLSKEE--IVKILNQ------KDVSQV---------EFGNAGK--- 145
G.adiacensIS48_PBP2                ----------SKHISLSKEE--IVKILSQ------KDVSQV---------EFGNAGK--- 145
G.adiacensGA01_PBP2                ----------SKHISLSKEE--IVKILSQ------KDVSQV---------EFGNAGK--- 145
G.adiacensKHUD009_PBP2             ----------SKHISLSKEE--IVKILSQ------KDVSQV---------EFGNAGK--- 145
E.faecalisOG1RF_PBP2               ----------AKYTKLDKKT--ALKYLNNGIHEDGSTQYQV---------EFGTGGQ--- 162
E.faeciumDO_PBP2                   ----------HKNLSIDKKD--ALNILEDGAK---KELYQV---------EFGSYGK--- 158
                                                           .

G.adiacensATCC49175_PBP1B          --------------------------NVSSFGRNH---------------NGLNIAGIEE 226
G.adiacensGA01_PBP1B               --------------------------NVSSFGRNH---------------NGLNIAGIEE 226
G.adiacensIS48_PBP1B               --------------------------NVSSFGRNH---------------NGLNIAGIEE 226
G.adiacensKHUD009_PBP1B            --------------------------NVSSFGRNH---------------NGLNIAGIEE 226
S.pneumoniaeD39_PBP1B              --------------------------NVAPFGRNN---------------KGQNIAGARQ 242
S.pneumoniaeR6_PBP1B               --------------------------NVAPFGRNN---------------KGQNIAGARQ 242
S.pneumoniaeNCTC7465_PBP1B         --------------------------NVAPFGRNN---------------KGQNIAGARQ 242
S.pyogenesATCC-BAA-595_PBP1B       --------------------------NVSPFGRNN---------------KGQNIAGVEE 217
E.faeciumDO_PBP1B                  --------------------------NVSPFGRNN---------------KGQNIAGVQE 218
E.faecalisOG1RF_PBP1B              --------------------------NVSPFGRNN---------------KGENIAGVEE 229
G.adiacensATCC49175_PBP2A          --------------------------NNAYFGNG--------------------AYGIEN 229
G.adiacensKHUD009_PBP2A            --------------------------NNAYFGNG--------------------AYGIEN 229
G.adiacensGA01_PBP2A               --------------------------NNAYFGNG--------------------AYGIEN 229
G.adiacensIS48_PBP2A               --------------------------NNAYFGNG--------------------AYGIEN 229
E.faecalisOG1RF_PBP2A              --------------------------NNSYFGNG--------------------VWGVQD 213
E.faeciumDO_PBP2A                  --------------------------NNAYFGNG--------------------VWGVQD 211
S.pyogenesATCC-BAA-595_PBP2A       --------------------------NNSYFGNG--------------------VWGVED 266
S.pneumoniaeR6_PBP2A               --------------------------NNAYFGNG--------------------VWGVED 219
S.pneumoniaeD39_PBP2A              --------------------------NNAYFGNG--------------------VWGVED 219
S.pneumoniaeNCTC7465_PBP2A         --------------------------NNAYFGNG--------------------VWGVED 219
G.adiacensKHUD009_PBP1A            --------------------------NKVYMANS--------------------VYGFGT 207
G.adiacensIS48_PBP1A               --------------------------NKVYMANS--------------------VYGFGT 207
G.adiacensATCC49175_PBP1A          --------------------------NKVYMANS--------------------VYGFGT 207
```

G.adiacensGA01_PBP1A	--------------------------NKVYMANS--------------------VYGFGT	207
E.faecalisOG1RF_PBP1A	--------------------------NKVYMANG--------------------FYGMET	212
E.faeciumDO_PBP1A	--------------------------NKVYMSNG--------------------LYGMET	216
S.pyogenes_ATCC-BAA-595_PBP1A	--------------------------NKVYMGNG--------------------NYGMLT	188
S.pneumoniaeNCTC7465_PBP1A	--------------------------NKVYMSNG--------------------NYGMQT	185
S.pneumoniaeR6_PBP1A	--------------------------NKVYMSNG--------------------NYGMQT	185
S.pneumoniaeD39_PBP1A	--------------------------NKVYMSNG--------------------NYGMQT	185
G.adiacensIS48_PBP2B	SSSQTYEMQLAHIPSDELNYSEAEKQVIAIFTKMNSAYSLSTVTLKNEGVTEQEVAKISE	212
G.adiacensGA01_PBP2B	SSSQTYEMQLAHISSDELNYSEAEKQVIAIFTKMNSAYSLSTVTLKNEGVTEQEVAKISE	212
G.adiacensATCC49175_PBP2B	SSSQTYEMQLAHIPSDELNYSEAEKQVIAIFTKMNSAYSLSTVTLKNEGVTEQEVAKISE	212
G.adiacensKHUD009_PBP2B	SSSQTYEMQLAHISSDELNYSEAEKQVIAIFTKMNSAYSLSTVTLKNEGVTEQEVAKISE	212
S.pneumoniaeR6_PBP2B	SESELYNNAVDSVQTSQLNYTEDEKKEIYLFSQLNAVGNFATGTIATDPLNDSQVAVIAS	212
S.pneumoniaeD39_PBP2B	SESELYNNAVDSVQTSQLNYTEDEKKEIYLFSQLNAVGNFATGTIATDPLNDSQVAVIAS	212
S.pneumoniaeNCTC7465_PBP2B	SESELYNNAVDSVQTSQLNYTEDEKKEIYLFSQLNAVGNFATGTIATDPLNDSQVAVIAS	207
E.faecalisOG1RF_PBP2B	DEGTLYAKAVEKVTPEEIAFDDRTLQAVTIFKRMNAASQMNTVFIKNEGVTEGEIATIGE	227
E.faeciumDO_PBP2B	GNSEQYSAMVNKVTADEINLNEEQLKIATIFKRMNSAYELNTVYIKNSGVTDQELAVVAE	228
E.faecalisOG1RF_PBP4	----------------------------DSFVPIT---------VAS-----------EP	249
E.faeciumDO_PBP5	----------------------------DYFVPLK---------IID-----------GA	247
S.pyogenesATCC-BAA-595_PBP2	--------------------------GISYSTMST---------IQK-----------AM	171
S.pneumoniaeR6_PBP2	--------------------------GITYANMMS---------IKK-----------EL	170
S.pneumoniaeD39_PBP2	--------------------------GITYANMMS---------IKK-----------EL	170
S.pneumoniaeNCTC7465_PBP2	--------------------------GITYANMMS---------IKK-----------EL	170
G.adiacensATCC49175_PBP2	--------------------------NLSVQVKDK---------IEA-------------	157
G.adiacensIS48_PBP2	--------------------------NLSVQVKDK---------IEA-------------	157
G.adiacensGA01_PBP2	--------------------------NLSVQVKDK---------IEA-------------	157
G.adiacensKHUD009_PBP2	--------------------------NLSVQVKDK---------IEA-------------	157
E.faecalisOG1RF_PBP2	--------------------------NITLETRQK---------IEA-----------DL	176
E.faeciumDO_PBP2	--------------------------NISQETKQN---------IEA-----------DM	172

G.adiacensATCC49175_PBP1B	AAQGVFGVAAKDL---TLPQAAYLVGMPQNPIVYTPYTNQATLKE--DTSAGIERMKFVL	281
G.adiacensGA01_PBP1B	AAQGVFGVAAKDL---TLPQAAYLVGMPQNPIVYTPYTNQATLKE--DTSAGIERMKFVL	281
G.adiacensIS48_PBP1B	AAQGVFGVAAKDL---TLPQAAYLVGMPQNPIVYTPYTNQATLKE--DTSAGIERMKFVL	281
G.adiacensKHUD009_PBP1B	AAQGVFGVAAKDL---TLPQAAYLVGMPQNPIVYTPYTNQATLKE--DTSAGIERMKFVL	281
S.pneumoniaeD39_PBP1B	AAEGIFGVDASQL---TVPQAAFLAGLPQSPITYSPYENTGELKSDEDLEIGLRRAKAVL	299
S.pneumoniaeR6_PBP1B	AAEGIFGVDASQL---TVPQAAFLAGLPQSPITYSPYENTGELKSDEDLEIGLRRAKAVL	299
S.pneumoniaeNCTC7465_PBP1B	AAEGIFGVDASQL---TVPQAAFLAGLPQSPITYSPYENTGELKSDEDLEIGLRRAKAVL	299
S.pyogenesATCC-BAA-595_PBP1B	AARGIFGVSAKDL---TVPQAAFLAGLPQSPIVYSPYLSTGQLKSEKDMAYGIKRQQNVL	274
E.faeciumDO_PBP1B	AARGIFGVDAKDV---TLPQAAYIAGLPQSPITYSPYTNTGALKE--DLSAGLARKDFVL	273
E.faecalisOG1RF_PBP1B	AAKGLFGKSAKDL---NLPQAAFIAGLPQSPIVYTPYTNTGALKD--DLSLGMKRKDFVL	284
G.adiacensATCC49175_PBP2A	ASLRYFGKSAKDL---TISEAAVLTGSLKAPNVYNPIDDMEATV---------KRRATVL	277
G.adiacensKHUD009_PBP2A	ASLRYFGKSAKDL---TISEAAVLTGSLKAPNVYNPIDDMEATV---------KRRATVL	277
G.adiacensGA01_PBP2A	ASLRYFGKSAKDL---TISEAAVLTGSLKAPNVYNPIDDMEATV---------KRRATVL	277

```
G.adiacensIS48_PBP2A            ASLRYFGKSAKDL---TISEAAVLTGSLKAPNVYNPIDDMEATV---------KRRATVL 277
E.faecalisOG1RF_PBP2A           AARKYFGVDASQV---TVGEAATLAGMLKGPGIYNPIDYIDNAT---------ARRNTVL 261
E.faeciumDO_PBP2A               ASRKYFGVDATNV---TLSEAATLAGMLKGPGIYNPIDHPKNAN---------DRKNTVL 259
S.pyogenesATCC-BAA-595_PBP2A    ASQKYFGTTAANL---TLDEAATLAGMLKGPEIYNPYHSLKNAT---------HRRDTVL 314
S.pneumoniaeR6_PBP2A            ASKKYFGVSASEV---SLDQAATLAGMLKGPELYNPLNSVEDST---------NRRDTVL 267
S.pneumoniaeD39_PBP2A           ASKKYFGVSASEV---SLDQAATLAGMLKGPELYNPLNSVEDST---------NRRDTVL 267
S.pneumoniaeNCTC7465_PBP2A      ASKKYFGVSASEV---SLDQAATLAGMLKGPELYNPLNSVEYST---------NRRDTVL 267
G.adiacensKHUD009_PBP1A         ASHYYFNKELSEL---TLPQVALLAGMPQAPNSYDPYAHPEEAK---------ERRDTVL 255
G.adiacensIS48_PBP1A            ASHYYFNKELSEL---SLPQVALLAGMPQAPNSYDPYAHPEEAK---------ERRDTVL 255
G.adiacensATCC49175_PBP1A       ASHYYFNKELSEL---SLPQVALLAGMPQAPNSYDPYAHPEEAK---------ERRDTVL 255
G.adiacensGA01_PBP1A            ASHYYFNKELSEL---SLPQVALLAGMPQAPNSYDPYAHPEEAK---------ERRDTVL 255
E.faecalisOG1RF_PBP1A           AAENYYGKHLSEL---DLPQTALLAGMPQAPNSYDPYTKPDTAK---------ERRDVVL 260
E.faeciumDO_PBP1A               ASEMYFGKKLSEL---SLPQTALLAGMPQAPSAYDPYVYPDQAK---------KRRDTVL 264
S.pyogenes_ATCC-BAA-595_PBP1A   AAKSYYGKDLKDL---SYAQLALLAGIPQAPSQYDPYLHPEAAQ---------NRRNVVL 236
S.pneumoniaeNCTC7465_PBP1A      AAQNYYGKDLNNL---SLPQLALLAGMPQAPNQYDPYSHPEAAQ---------DRRNLVL 233
S.pneumoniaeR6_PBP1A            AAQNYYGKDLNNL---SLPQLALLAGMPQAPNQYDPYSHPEAAQ---------DRRNLVL 233
S.pneumoniaeD39_PBP1A           AAQNYYGKDLNNL---SLPQLALLAGMPQAPNQYDPYSHPEAAQ---------DRRNLVL 233
G.adiacensIS48_PBP2B            RLGELRGVDIDSDWDREYPMGDMLKTIL-------------------------------G 241
G.adiacensGA01_PBP2B            RLGELRGVDIDSDWDREYPMGDMLKTIL-------------------------------G 241
G.adiacensATCC49175_PBP2B       RLGELRGVDIDSDWDREYPMGDMLKTIL-------------------------------G 241
G.adiacensKHUD009_PBP2B         RLGELRGVDIDSDWDREYPMGDMLKTIL-------------------------------G 241
S.pneumoniaeR6_PBP2B            ISKEMPGISISTSWDRKVLE-TSLSSIV-------------------------------G 240
S.pneumoniaeD39_PBP2B           ISKEMPGISISTSWDRKVLE-TSLSSIV-------------------------------G 240
S.pneumoniaeNCTC7465_PBP2B      ISKEMPGISISTSWDRKVLE-TSLSSIV-------------------------------G 235
E.faecalisOG1RF_PBP2B           HTAEISGVSTGTDWTRDYSQSGALRSLL-------------------------------G 256
E.faeciumDO_PBP2B               RASELPGVSTGTDWTREYNAASSLKSIL-------------------------------G 257
E.faecalisOG1RF_PBP4            VTELPTGAATKDTESRYYPLGEAAAQLI-------------------------------G 278
E.faeciumDO_PBP5                TPELPAGATIQEVDGRYYPLGEAAAQLI-------------------------------G 276
S.pyogenesATCC-BAA-595_PBP2     EDAKIKGIAFTTSPGRMYPNGTFASEFI-------------------------------G 200
S.pneumoniaeR6_PBP2             EAAEVKGIDFTTSPNRSYPNGQFASSFI-------------------------------G 199
S.pneumoniaeD39_PBP2            EAAEVKGIDFTTSPNRSYPNGQFASSFI-------------------------------G 199
S.pneumoniaeNCTC7465_PBP2       EAAEVKGIDFTTSPNRSYPNGQFASSFI-------------------------------G 199
G.adiacensATCC49175_PBP2        --EKLPGIKFSESPARYYPNGIFASHLI-------------------------------G 184
G.adiacensIS48_PBP2             --EKLPGIKFSESPARYYPNGIFASHLI-------------------------------G 184
G.adiacensGA01_PBP2             --EKLPGIKFSESPARYYPNGIFASHLI-------------------------------G 184
G.adiacensKHUD009_PBP2          --EKLPGIKFSESPARYYPNGIFASHLI-------------------------------G 184
E.faecalisOG1RF_PBP2            KKKKISGVYFNEHPARLYPNGQFASHFI-------------------------------G 205
E.faeciumDO_PBP2                KKEGVAGLYFVDHQARMYPNGVFSSHFI-------------------------------G 201
                                      .

G.adiacensATCC49175_PBP1B       FSMYRENKITKEQYEEALAYDITKDFLPPKEIS-SDRQSYLYQ----AVNREAIKVLITK 336
G.adiacensGA01_PBP1B            FSMYRENKITKEQYEEALAYDITKDFLPPKEIS-SDRQSYLYQ----AVNREAIKVLITK 336
G.adiacensIS48_PBP1B            FSMYRENKITKEQYEEALAYDITKDFLPPKEIS-SDRQSYLYQ----AVNREAIKVLITK 336
```

```
G.adiacensKHUD009_PBP1B          FSMYRENKITKEQYEEALAYDITKDFLPPKEIS-SDRQSYLYQ----AVNREAIKVLITK 336
S.pneumoniaeD39_PBP1B            YSMYRTGALSKDEYSQYKDYDLKQDFLPSGTVT-GISRDYLYF----TTLAEAQERMYDY 354
S.pneumoniaeR6_PBP1B             YSMYRTGALSKDEYSQYKDYDLKQDFLPSGTVT-GISRDYLYF----TTLAEAQERMYDY 354
S.pneumoniaeNCTC7465_PBP1B       YSMYRTGALSKDEYSQYKDYDLKQDFLPSGTVT-GISRDYLYF----TTLAEAQERMYDY 354
S.pyogenesATCC-BAA-595_PBP1B     YNMYRTGVLSKKEYEDYKAYPIQKDFIQPGSAI-VNNHDYLYY----TVLADAKKAMYSY 329
E.faeciumDO_PBP1B                FSMYREGQITKEQYEEAKAYDLTKDFLPQQIAE-QTDREFLYY----TVMNEATRIIAQQ 328
E.faecalisOG1RF_PBP1B            FSMYREKAISQKEYEEAKAYDLKKDFLPTEQAN-VNTEGYLYY----TVLDKAVEIVMDL 339
G.adiacensATCC49175_PBP2A        QLMVTNGKITQEEADKAGAEVIT--VQDAYV----ANSRYKYPYYFDAVINEIT------ 325
G.adiacensKHUD009_PBP2A          QLMVTNGKITQEEADKAGAEVIT--VQDAYV----ANSRYKYPYYFDAVINEIT------ 325
G.adiacensGA01_PBP2A             QLMVTNGKITQEEADKAGAEVIT--VQDAYV----ANSRYKYPYYFDAVINEIT------ 325
G.adiacensIS48_PBP2A             QLMVTNGKITQEEADKAGAEVIT--VQDAYV----ANSRYKYPYYFDAVINEIT------ 325
E.faecalisOG1RF_PBP2A            QLMVDNKKLSQEEANQEASVNLASLLNDTYV---GDENSYKYPYYFDAVIDEAV------ 312
E.faeciumDO_PBP2A                SVMEENGKITKEQEQAESQTDISAYLNDTYD---NSDSGYRYPYYFDAVIDEAV------ 310
S.pyogenesATCC-BAA-595_PBP2A     GAMVDAKKITQTKAQQARAVGLKNRLADTYV---GKTDDYKYPSYFDAVISEAI------ 365
S.pneumoniaeR6_PBP2A             QNMVAAGYIDKNQETEAAEVDMTSQLHDKYE---GKISDYRYPSYFDAVVNEAV------ 318
S.pneumoniaeD39_PBP2A            QNMVAAGYIDKNQETEAAEVDMTSQLHDKYE---GKISDYRYPSYFDAVVNEAV------ 318
S.pneumoniaeNCTC7465_PBP2A       QNMVAAGYIDKNQETEAAEVDMTSQLHDKYE---GKISDYRYPSYFDAVVNEAV------ 318
G.adiacensKHUD009_PBP1A          YTMKTNGKITNEQYEQALATPINDGLIAHNNNVDSSDKALVYDSFVTMVLKEVQ------ 309
G.adiacensIS48_PBP1A             YTMKTNGKITNEQYEQALATPINDGLIAHDNNVDSSDKALVYDSFVTMVLKEVQ------ 309
G.adiacensATCC49175_PBP1A        YTMKTNGKITNEQYEQALATPINDGLIAHDNNVDSSDKALVYDSFVTMVLKEVQ------ 309
G.adiacensGA01_PBP1A             YTMKTNGKITNEQYEQALATPINDGLIAHDNNVDSSDKALVYDSFVTMVLKEVQ------ 309
E.faecalisOG1RF_PBP1A            YTMYDNKKISKAEYEKAKATPIDEGLVPLKA---SDDNRKVVDNYVKEVINEVK------ 311
E.faeciumDO_PBP1A                YTMLQNEKISQTEYDQAVNVPVTDGLQELTQ---SDDNTKIVDNYVKEVINEVQ------ 315
S.pyogenes_ATCC-BAA-595_PBP1A    QQMYMEKHLTKAEYETAIATPVAEGLQSLQQ---RSTYPKYMDNYLKQVIEEVK------ 287
S.pneumoniaeNCTC7465_PBP1A       SEMKNQGYISAEQYEKAVNTPITDGLQSLKS---ASNYPAYMDNYLKEVINQVE------ 284
S.pneumoniaeR6_PBP1A             SEMKNQGYISAEQYEKAVNTPITDGLQSLKS---ASNYPAYMDNYLKEVINQVE------ 284
S.pneumoniaeD39_PBP1A            SEMKNQGYISAEQYEKAVNTPITDGLQSLKS---ASNYPAYMDNYLKEVINQVE------ 284
G.adiacensIS48_PBP2B             TVSSEKTGLPSSKVK--------SLLAQGYSLNDRVGTSYLEEQYEN-VLSGSK------ 286
G.adiacensGA01_PBP2B             TVSSEKTGLPSSKVK--------SLLAQGYSLNDRVGTSYLEEQYEN-VLSGSK------ 286
G.adiacensATCC49175_PBP2B        TVSSEKTGLPSSKVK--------SLLSQGYSLNDRVGTSYLEEQYEN-VLSGSK------ 286
G.adiacensKHUD009_PBP2B          TVSSEKTGLPSSKVK--------SLLAQGYSLNDRVGTSYLEEQYEN-VLSGSK------ 286
S.pneumoniaeR6_PBP2B             SVSSEKAGLPAEEAE--------AYLKKGYSLNDRVGTSYLEKQYEE-TLQGKR------ 285
S.pneumoniaeD39_PBP2B            SVSSEKAGLPAEEAE--------AYLKKGYSLNDRVGTSYLEKQYEE-TLQGKR------ 285
S.pneumoniaeNCTC7465_PBP2B       SVSSEKAGLPAEEAE--------AYLKKGYSLNDRVGTSYLEKQYEE-TLQGKR------ 280
E.faecalisOG1RF_PBP2B            TVSTEKQGLPAEEVD--------EYLKKGYARNDRVGTSYLEKQYED-VLQGKK------ 301
E.faeciumDO_PBP2B                SVTTEKQGLPADEAE--------KYLAKGYSRNDRVGQSYLEKQYED-VLQGTK------ 302
E.faecalisOG1RF_PBP4             YTGTITAE------D--------IEKNPELSSTGVIGKTGLERAFDK-ELRGQD------ 317
E.faeciumDO_PBP5                 YVGDITAE------D--------IDKNPELSSNGKIGRSGLEMAFDK-DLRGTT------ 315
S.pyogenesATCC-BAA-595_PBP2      LASLTED--------------------KKTGVKSLVGKTGLEASFDK-ILSGQD------ 233
S.pneumoniaeR6_PBP2              LAQLHEN---------------------EDGSKSLLGTSGMESSLNS-ILAGTD------ 231
S.pneumoniaeD39_PBP2             LAQLHEN---------------------EDGSKSLLGTSGMESSLNS-ILAGTD------ 231
S.pneumoniaeNCTC7465_PBP2        LAQLHEN---------------------EDGSKSLLGTSGMESSLNS-ILAGTD------ 231
G.adiacensATCC49175_PBP2         YTDSVEET------------------VDHKTTISLVGKTGLEGLYNE-QLTGTA------ 219
```

```
G.adiacensIS48_PBP2             YTDSVEET------------------VDHKTTISLVGKTGLEGLYNE-QLTGTA------ 219
G.adiacensGA01_PBP2             YTDSVEET------------------VDHKTTISLVGKTGLEGLYNE-QLTGTA------ 219
G.adiacensKHUD009_PBP2          YTDSVEET------------------VDHKTTISLVGKTGLEGLYNE-QLTGTA------ 219
E.faecalisOG1RF_PBP2            YTKAANP---------------------DDDKEGLVGAMGLEQTYND-ILSGTD------ 237
E.faeciumDO_PBP2                YA---VP---------------------DKDENGLVGKLGLESAYND-ILSGKD------ 230


G.adiacensATCC49175_PBP1B       AAQKNKLTYDEVRNDSELYSKYYDEASRELSSSGYKITSTIDQKVYDAMQKAIASYGNQ- 395
G.adiacensGA01_PBP1B            AAQKNKLTYDEVRNDSELYSKYYDEASRELSSSGYKITSTIDQKVYDAMQKAIASYGNQ- 395
G.adiacensIS48_PBP1B            AAQKNKLTYDEVRNDSELYSKYYDEASRELSSSGYKITSTIDQKVYDAMQKAIASYGNQ- 395
G.adiacensKHUD009_PBP1B         AAQKNKLTYDEVRNDSELYSKYYDEASRELSSSGYKITSTIDQKVYDAMQKAIASYGNQ- 395
S.pneumoniaeD39_PBP1B           LAQRDNVSAKELKNEA-TQKFYRDLAAKEIENGGYKITTTIDQKIHSAMQSAVADYGYL- 412
S.pneumoniaeR6_PBP1B            LAQRDNVSAKELKNEA-TQKFYRDLAAKEIENGGYKITTTIDQKIHSAMQSAVADYGYL- 412
S.pneumoniaeNCTC7465_PBP1B      LAQRDNVSAKELKNEA-TQKFYRDLAAKEIENGGYKITTTIDQKIHSAMQSAVADYGYL- 412
S.pyogenesATCC-BAA-595_PBP1B    LIKRDKVSSRDLKNDE-TKAAYEERALTELQQGGYTITTTINKPIYNAMQTAAAQFGGL- 387
E.faeciumDO_PBP1B               LAEKD--NADMSDS-S-VSDAYYQKAQQTIQNKGYTIHSTIDKDVYAALQNGVENYGYM- 383
E.faecalisOG1RF_PBP1B           DMKKAKVNRDDLDQ-V-GLDQYEEQARREIQSQGYTIQSTIDQNIYNTMQTAVANYGYL- 396
G.adiacensATCC49175_PBP2A       --------------------NNYGISEEELLNKGYKIYTGLDQKMQADMEETFSNSWLF- 364
G.adiacensKHUD009_PBP2A         --------------------NNYGISEEELLNKGYKIYTGLDQKMQADMEETFSNSWLF- 364
G.adiacensGA01_PBP2A            --------------------NNYGISEEELLNKGYKIYTGLDQKMQADMEETFSNSWLF- 364
G.adiacensIS48_PBP2A            --------------------NNYGISEEELLNKGYKIYTGLDQKMQADMEETFSNSWLF- 364
E.faecalisOG1RF_PBP2A           --------------------NRYKFKEEDILNKGYKIYTSLNQKYQDAMDATYKNDALF- 351
E.faeciumDO_PBP2A               --------------------NEYGLDEEDVMNKGYKIYTALDQDYQKQLEATYQNSALF- 349
S.pyogenesATCC-BAA-595_PBP2A    --------------------ATYGLSEKDIVNNGYKVYTELDQNYQTGMQTTFNNDELF- 404
S.pneumoniaeR6_PBP2A            --------------------SKYNLTEEEIVNNGYRIYTELDQNYQANMQIVYENTSLF- 357
S.pneumoniaeD39_PBP2A           --------------------SKYNLTEEEIVNNGYRIYTELDQNYQANMQIVYENTSLF- 357
S.pneumoniaeNCTC7465_PBP2A      --------------------SKYNLTEEEIVNNGYRIYTELDQNYQANMQIVYENTSLF- 357
G.adiacensKHUD009_PBP1A         --------------------DK---TGLDPYNDGLVIETTIDSKAQQRLNDIVNTNDYI- 345
G.adiacensIS48_PBP1A            --------------------DK---TGLDPYNDGLVIETTIDSKAQQKLNDIVNTNDYI- 345
G.adiacensATCC49175_PBP1A       --------------------DK---TGLDPYNDGLVIETTIDSKAQQKLNDIVNTNDYI- 345
G.adiacensGA01_PBP1A            --------------------DK---TGLDPYNDGLVIETTIDSKAQQKLNDIVNTNDYI- 345
E.faecalisOG1RF_PBP1A           --------------------AK---TGKNVYTDGLDIYTNLDMNAQKQLYDIVNSDQYV- 347
E.faeciumDO_PBP1A               --------------------EK---TDKNVYTDGLEIYTNLDLDAQKKLYDIVNTDQYV- 351
S.pyogenes_ATCC-BAA-595_PBP1A   --------------------KE---TNKDIFTAGLKVYTNIIPDAQQTLYNIYHSGDYV- 323
S.pneumoniaeNCTC7465_PBP1A      --------------------EE---TGYNLLTTGMDVYTNVDQEAQKHLWDIYNTDEYV- 320
S.pneumoniaeR6_PBP1A            --------------------EE---TGYNLLTTGMDVYTNVDQEAQKHLWDIYNTDEYV- 320
S.pneumoniaeD39_PBP1A           --------------------EE---TGYNLLTTGMDVYTNVDQEAQKHLWDIYNTDEYV- 320
G.adiacensIS48_PBP2B            -----TVVQSQTNTKGQV--I--KNNETYPGKAGSNLVLTIDTEFQKKIEEIAKTSVEE- 336
G.adiacensGA01_PBP2B            -----TVVQSQTNTKGQV--I--KNNETYPGKAGSNLVLTIDTEFQKKIEEIAKTSVEE- 336
G.adiacensATCC49175_PBP2B       -----TVVQSQTNTKGQV--I--KNNETYPGKAGSNLVLTIDTEFQKKIEEIAKTSVEE- 336
G.adiacensKHUD009_PBP2B         -----TVVQSQTNTKGQV--I--KNNETYPGKAGSNLVLTIDTEFQKKIEEIAKTSVEE- 336
S.pneumoniaeR6_PBP2B            -----SVKEIHLDKYGNM--E--SVDTIEEGSKGNNIKLTIDLAFQDSVDALLKSYFNSE 336
S.pneumoniaeD39_PBP2B           -----SVKEIHLDKYGNM--E--SVDTIEEGSKGNNIKLTIDLAFQDSVDALLKSYFNSE 336
```

```
S.pneumoniaeNCTC7465_PBP2B          -----SVKEIHLDKYGNM--E--SVDTIEEGSKGNNIKLTIDLAFQDSVDALLKSYFNSE 331
E.faecalisOG1RF_PBP2B               -----AKSEVVLDNNGKI--V--SQTPISKGEKGSNLKLTIDSNFQNKVDEILQRNYSQI 352
E.faeciumDO_PBP2B                   -----TQYEVSLDNEGNV--S--NQKEIFSGEKGSNLMLSMNAEFQSKVEEILKRNYQTL 353
E.faecalisOG1RF_PBP4                -----GGSLVILDDKENV--K--KALQTKEKKDGQTIKLTIDSGVQQQAFA-------I- 360
E.faeciumDO_PBP5                    -----GGKLSITDADGVE--K--KVLIEHEVQNGKDIKLTIDAKAQKTAFD-------S- 358
S.pyogenesATCC-BAA-595_PBP2         -----GVITYQKDRNGAT--LLGTGKTVKKAIDGKDIYTTLSEPIQTFLETQMDVFQAK- 285
S.pneumoniaeR6_PBP2                 -----GIITYEKDRLGNI--VPGTEQVSQRTMDGKDVYTTISSPLQSFMETQMDAFQEK- 283
S.pneumoniaeD39_PBP2                -----GIITYEKDRLGNI--VPGTEQVSQRTMDGKDVYTTISSPLQSFMETQMDAFQEK- 283
S.pneumoniaeNCTC7465_PBP2           -----GIITYEKDRLGNI--VPGTEQVSQRTMDGKDVYTTISSPLQSFMETQMDAFQEK- 283
G.adiacensATCC49175_PBP2            -----GEVEYTVDGNGYV--ISDTEKVTKQPKDGKDITLTLDKRLQTYLESLVSAADKQ- 271
G.adiacensIS48_PBP2                 -----GEVEYTVDGNGYV--ISDTEKVTKQPKDGKDITLTLDKRLQTYLESLVSAADKQ- 271
G.adiacensGA01_PBP2                 -----GEVEYTVDGNGYV--ISDTEKVTKQPKDGKDITLTLDKRLQTYLESLVSAADKQ- 271
G.adiacensKHUD009_PBP2              -----GEVEYTVDGNGYV--ISDTEKVTKQPKDGKDITLTLDKRLQTYLESLVSAADKQ- 271
E.faecalisOG1RF_PBP2                -----GRVYFEKDIYGNA--LPGTVAEEKKAVDGQDIYTTLDSRLQNTLEDLMTQVNEK- 289
E.faeciumDO_PBP2                    -----GKIIYQKDNFQNP--LPGTVAEEEKAVDGQDIYTTLDSRLQSYLETLMDQVNEE- 282
                                                                     *  :   :

G.adiacensATCC49175_PBP1B           IGPTYNTQYVDQNTGETKTQTEPAQNGAVMIENKTGRILGFVAGRD-FESN-------QV 447
G.adiacensGA01_PBP1B                IGPTYNTQYVDQNTGETKTQTEPAQNGAVMIENKTGRILGFVAGRD-FESN-------QV 447
G.adiacensIS48_PBP1B                IGPTYNTQYVDQNTGETKTQTEPAQNGAVMIENKTGRILGFVAGRD-FESN-------QV 447
G.adiacensKHUD009_PBP1B             IGPTYNTQYVDQNTGETKTQTEPAQNGAVMIENKTGRILGFVAGRD-FESN-------QV 447
S.pneumoniaeD39_PBP1B               LDD---------GTG-------RVEVGNVLMDNQTGAILGFVGGRN-YQEN-------QN 448
S.pneumoniaeR6_PBP1B                LDD---------GTG-------RVEVGNVLMDNQTGAILGFVGGRN-YQEN-------QN 448
S.pneumoniaeNCTC7465_PBP1B          LDD---------GTG-------RVEVGNVLMDNQTGAILGFVGGRN-YQEN-------QN 448
S.pyogenesATCC-BAA-595_PBP1B        LDD---------GTG-------TVQMGNVLTDNATGAVLGFVGGRD-YALN-------QN 423
E.faeciumDO_PBP1B                   LDD---------GAG------SQVETGNVLMDNRTGRIYGFVGGRN-YSQN-------QN 420
E.faecalisOG1RF_PBP1B               LDD---------GTADVN-GNTMIETGNILMDNATGRILGFIGGRN-FDIN-------QN 438
G.adiacensATCC49175_PBP2A           PKA-S--------------DGTIAQAASVAMDPQNGDVLATVGGRT--NEKHTFR---GF 404
G.adiacensKHUD009_PBP2A             PKA-S--------------DGTIAQAASVAMDPQNGDVLATVGGRT--NEKHTFR---GF 404
G.adiacensGA01_PBP2A                PKA-S--------------DGTIAQAASVAMDPQNGDVLATVGGRT--NEKHTFR---GF 404
G.adiacensIS48_PBP2A                PKA-S--------------DGTIAQAASVAMDPQNGDVLATVGGRT--NEKHTFR---GF 404
E.faecalisOG1RF_PBP2A               PPNAE--------------DGAMVQSGSVAIDPKTGGVQALVGGRG-E---HVYR---GF 390
E.faeciumDO_PBP2A                   PANAS--------------DGAMVQSASVALDPNTGGVQALVGRRG-D---HVFR---GF 388
S.pyogenesATCC-BAA-595_PBP2A        PVSAY--------------DGSSAQAASVALDPKTGGVRGLIGRVN-SSENPTFR---SF 446
S.pneumoniaeR6_PBP2A                PRA-E--------------DGTFAQSGSVALEPKTGGVRGVVGQVA-DNDKTGFR---NF 398
S.pneumoniaeD39_PBP2A               PRA-E--------------DGTFAQSGSVALEPKTGGVRGVVGQVA-DNDKTGFR---NF 398
S.pneumoniaeNCTC7465_PBP2A          PRA-E--------------DGTSAQSGSVALEPKTGGVRGVVGQVA-DNDKTGFR---NF 398
G.adiacensKHUD009_PBP1A             N---Y--------------VNDKIQSASVMLDSKTGAVRAVSGGRK-QTT---LF---AY 381
G.adiacensIS48_PBP1A                N---Y--------------INDKIQSASVMLDSKTGAVRAVSGGRK-QTT---LF---AY 381
G.adiacensATCC49175_PBP1A           N---Y--------------VNDKIQSASVMLDSKTGAVRAVSGGRK-QTT---LF---AY 381
G.adiacensGA01_PBP1A                N---Y--------------VNDKIQSASVMLDSKTGAVRAVSGGRK-QTT---LF---AY 381
E.faecalisOG1RF_PBP1A               A---F--------------PDDKMQVASTVIDVASGQVRAQIGGRH-IPDD-VQL---GN 385
E.faeciumDO_PBP1A                   S---Y--------------PDDEMQVASTLIDTNTGKVKAQIGGRH-IAED-VTL---GN 389
```

```
S.pyogenes_ATCC-BAA-595_PBP1A      Y---Y--------------PDQDFQVASTIVDVTNGHVIAQLGGRN-QDEN-VSF---GT 361
S.pneumoniaeNCTC7465_PBP1A         A---Y--------------PDDELQVASTIVDVSNGKVIAQLGARH-QSSN-VSF---GI 358
S.pneumoniaeR6_PBP1A               A---Y--------------PDDELQVASTIVDVSNGKVIAQLGARH-QSSN-VSF---GI 358
S.pneumoniaeD39_PBP1A              A---Y--------------PDDELQVASTIVDVSNGKVIAQLGARH-QSSN-VSF---GI 358
G.adiacensIS48_PBP2B               ---MT--------------DPAADRVYIVVMNPKNGDVLGITGKKKKFDGNFQSNGVEDD 379
G.adiacensGA01_PBP2B               ---MT--------------DPAADRVYIVVMNPKNGDVLGITGKKKKFDGNFQSNGVEDD 379
G.adiacensATCC49175_PBP2B          ---MT--------------DPAADRVYIVVMNPKNGDVLGITGKKKKFDGNFQSNGVEDD 379
G.adiacensKHUD009_PBP2B            ---MT--------------DPAADRVYIVVMNPKNGDVLGITGKKKKFDGNFQSNGVEDD 379
S.pneumoniaeR6_PBP2B               LENGG--------------AKYSEGVYAVALNPKTGAVLSMSGIKH----DLKTGELTPD 378
S.pneumoniaeD39_PBP2B              LENGG--------------AKYSEGVYAVALNPKTGAVLSMSGIKH----DLKTGELTPD 378
S.pneumoniaeNCTC7465_PBP2B         LENGG--------------AKYSEGVYAVALNPKTGAVLSMSGIKH----DLKTGELTPD 373
E.faecalisOG1RF_PBP2B              VK-TI--------------GPYSENAYVVAMNPQTGAILAMSGFHH----DLATGEVTPN 393
E.faeciumDO_PBP2B                  INNGK--------------AQYSPGAYAVAMNPQTGEVLAMTGFSH----EQGSKEITEN 395
E.faecalisOG1RF_PBP4               FD--------------------KRPGSAVITDPQKGDLLATVSSPS-YDPNKMANGISQK 399
E.faeciumDO_PBP5                   LG--------------------GKAGSTVATTPKTGDLLALASSPS-YDPNKMTNGISQE 397
S.pyogenesATCC-BAA-595_PBP2        SN--------------------GQLASATLVNAKTGEILATTQRPT-YNADTL-KGLENT 323
S.pneumoniaeR6_PBP2                VK--------------------GKYMTATLVSAKTGEILATTQRPT-FDADTK-EGIT-E 320
S.pneumoniaeD39_PBP2               VK--------------------GKYMTATLVSAKTGEILATTQRPT-FDADTK-EGIT-E 320
S.pneumoniaeNCTC7465_PBP2          VK--------------------GKYMTATLVSAKTGEILATTQRPT-FDADTK-EGIT-E 320
G.adiacensATCC49175_PBP2           YQ--------------------PVQMTAMLVDPKSGNIVAATQRPT-YNSTTK-EGID-- 307
G.adiacensIS48_PBP2                YQ--------------------PVQMTAMLVDPKSGNIVAATQRPT-YNSTTK-EGID-- 307
G.adiacensGA01_PBP2                YQ--------------------PVQMTAMLVDPKSGNIVAATQRPT-YNSTTK-EGID-- 307
G.adiacensKHUD009_PBP2             YQ--------------------PVQMTAMLVDPKSGNIVAATQRPT-YNSTTK-EGID-- 307
E.faecalisOG1RF_PBP2               YE--------------------PVSMTAMLMEAKTGEIVAMSQRPT-FNPETK-QGLDD- 326
E.faeciumDO_PBP2                   YQ--------------------PEELTAVLMKAKTGEILAMGQRPT-FNPETM-EGLTGK 320
                                                                     .* : .

G.adiacensATCC49175_PBP1B          DH-------------AFTTHRSPGSTIKPILVYAPAIENNLIY-PASIVPD-TKVSIPQG 492
G.adiacensGA01_PBP1B               DH-------------AFTTHRSPGSTIKPILVYAPAIENNLIY-PASIVPD-TKVSIPQG 492
G.adiacensIS48_PBP1B               DH-------------AFTTHRSPGSTIKPILVYAPAIENNLIY-PASIVPD-TKVSIPQG 492
G.adiacensKHUD009_PBP1B            DH-------------AFTTHRSPGSTIKPILVYAPAIENNLIY-PASIVPD-TKVSIPQG 492
S.pneumoniaeD39_PBP1B              NH-------------AFDTKRSPASTTKPLLAYGIAIDQGLMG-SETILSN-YPTNFANG 493
S.pneumoniaeR6_PBP1B               NH-------------AFDTKRSPASTTKPLLAYGIAIDQGLMG-SETILSN-YPTNFANG 493
S.pneumoniaeNCTC7465_PBP1B         NH-------------AFDTKRSPASTTKPLLAYGIAIDQGLMG-SETILSN-YPTNFANG 493
S.pyogenesATCC-BAA-595_PBP1B       NH-------------AFNTVRSPGSSIKPIIAYGPAIDQGLMG-SASVLSN-YPTTYSSG 468
E.faeciumDO_PBP1B                  NH-------------AFDTERQAGSSIKPVLVYGPAIDMGLIG-SESRVSD-YATTWQEG 465
E.faecalisOG1RF_PBP1B              NH-------------AFNADRQVGSTIKPISVYGPAIDQGLIG-SESRLAN-YPTTYADG 483
G.adiacensATCC49175_PBP2A          NR-------------ATQLQAQPGSTFKPLAVYTSALEEGYQ--PNSVLVD-EKRSYGSD 448
G.adiacensKHUD009_PBP2A            NR-------------ATQLQAQPGSTFKPLAVYTSALEEGYQ--PNSVLVD-EKRSYGSD 448
G.adiacensGA01_PBP2A               NR-------------ATQLQAQPGSTFKPLAVYTSALEEGYQ--PNSVLVD-EKRSYGSD 448
G.adiacensIS48_PBP2A               NR-------------ATQLQAQPGSTFKPLAVYTSALEEGYQ--PNSVLVD-EKRSYGSD 448
E.faecalisOG1RF_PBP2A              NF-------------ATQTKRSPGSSLKPISVYTPALEAGYK--PDSVLED-KPQDY--- 431
E.faeciumDO_PBP2A                  SF-------------ATQMRRSPGSTIKPLSVYTPALEAGYK--PSSILKD-EPQSY--- 429
```

```
S.pyogenesATCC-BAA-595_PBP2A        NY-------------ATQAKRSPASTIKPLVVYAPAVASGWS--IEKELPN-TVQDF--D 488
S.pneumoniaeR6_PBP2A                NY-------------ATQSKRSPGSTIKPLVVYTPAVEAGWA--LNKQLDN-HTMQY--D 440
S.pneumoniaeD39_PBP2A               NY-------------ATQSKRSPGSTIKPLVVYTPAVEAGWA--LNKQLDN-HTMQY--D 440
S.pneumoniaeNCTC7465_PBP2A          NY-------------ATQSKRSPGSTIKPLVVYTPAVEAGWA--LNKQLDN-HTMQY--D 440
G.adiacensKHUD009_PBP1A             NR-------------ATDNQRSTGSTIKPIIDYGPAIEYLNYS-TGQTLLD-QKTTYS-N 425
G.adiacensIS48_PBP1A                NR-------------ATDNQRSTGSTIKPIIDYGPAIEYLNYS-TGQTLLD-QKTTYS-N 425
G.adiacensATCC49175_PBP1A           NR-------------ATDNQRSTGSTIKPIIDYGPAIEYLNYS-TGQTLLD-QKTTYS-N 425
G.adiacensGA01_PBP1A                NR-------------ATDNQRSTGSTIKPIIDYGPAIEYLNYS-TGQTLLD-QKTTYS-N 425
E.faecalisOG1RF_PBP1A               NL-------------AVNTQRDVGSTVKPIMDYGPAIENLNYS-TGRLMVD-KPTKYPGT 430
E.faeciumDO_PBP1A                   NL-------------AVNTSRDFGSTMKPVTDYGPAFEYLKYS-TGKTITD-APYNYEGT 434
S.pyogenes_ATCC-BAA-595_PBP1A       NQ-------------AVLTDRDWGSTMKPITAYAPAIESGVYTSTAQSTND-SVYYWPGT 407
S.pneumoniaeNCTC7465_PBP1A          NQ-------------AVETNRDWGSTMKPITDYAPALEYGVYDSTATIVHD-EPYNYPGT 404
S.pneumoniaeR6_PBP1A                NQ-------------AVETNRDWGSTMKPITDYAPALEYGVYESTATIVHD-EPYNYPGT 404
S.pneumoniaeD39_PBP1A               NQ-------------AVETNRDWGSTMKPITDYAPALEYGVYDSTATIVHD-EPYNYPGT 404
G.adiacensIS48_PBP2B                AL------------GAINNSFGMGSVVKPATV-LLGYMDGALTLEDNKIVD-EPIEFEAS 425
G.adiacensGA01_PBP2B                AL------------GAINNSFGMGSVVKPATV-LSGYMDGALTLEDNKIVD-EPIEFEAS 425
G.adiacensATCC49175_PBP2B           AL------------GAINNSFGMGSVVKPATV-LSGYMDGALTLEDNKIVD-EPIEFEAS 425
G.adiacensKHUD009_PBP2B             AL------------GAINNSFGMGSVVKPATV-LSGYMDGALTLEDNKIVD-EPIEFEAS 425
S.pneumoniaeR6_PBP2B                SL------------GTVTNVFVPGSVVKAATI-SSGWENGVLSG-NQTLTD-QSIVFQGS 423
S.pneumoniaeD39_PBP2B               SL------------GTVTNVFVPGSVVKAATI-SSGWENGVLSG-NQTLTD-QSIVFQGS 423
S.pneumoniaeNCTC7465_PBP2B          SL------------GTVTNVFVPGSVVKAATI-SSGWENGVLSG-NQTLTD-QSIVFQGS 418
E.faecalisOG1RF_PBP2B               PL------------APILNSEVPGSVVKAGTL-TAGYETGVIKG-NDVLTD-EAILLAGS 438
E.faeciumDO_PBP2B                   AL------------GTITSAFAPGSVVKAGTL-TAGWASNAISG-NQVLID-EPIRLQGA 440
E.faecalisOG1RF_PBP4                EYDAYNNNKDLPFTARFATGYAPGSTFKTITG-AIGLDAGTLKP-DEELEI-NGLKWQKD 456
E.faeciumDO_PBP5                    DYKAYEENPEQPFISRFATGYAPGSTFKMITA-AIGLDNGTIDP-NEVLTI-NGLKWQKD 454
S.pyogenesATCC-BAA-595_PBP2         NYKWYSA--------LHQGNFEPGSTMKVMTL-AAAIDDKVFNP-NETFSNANGLTIADA 373
S.pneumoniaeR6_PBP2                 DFVWRDI--------LYQSNYEPGSTMKVMML-AAAIDNNTFPG-GEVF-NSSELKIADA 369
S.pneumoniaeD39_PBP2                DFVWRDI--------LYQSNYEPGSTMKVMML-AAAIDNNTFPG-GEVF-NSSELKIADA 369
S.pneumoniaeNCTC7465_PBP2           DFVWRDI--------LYQSNYEPGSTMKVMML-AAAIDNNTFPG-GEVF-NSSELKIADA 369
G.adiacensATCC49175_PBP2            -VQWNNL--------LTDQAYEPGSTMKVLAL-AAAINEGVFDP-NERYQS-GSVKIYTD 355
G.adiacensIS48_PBP2                 -VQWNNL--------LTDQAYEPGSTMKVLAL-AAAINEGVFDP-NERYQS-GSVKIYTD 355
G.adiacensGA01_PBP2                 -VQWNNL--------LTDQAYEPGSTMKVLAL-AAAINEGVFDP-NERYQS-GSVKIYTD 355
G.adiacensKHUD009_PBP2              -VQWNNL--------LTDQAYEPGSTMKVLAL-AAAINEGVFDP-NERYQS-GSVKIYTD 355
E.faecalisOG1RF_PBP2                NGTWQNL--------LVESPYEPGSTIKLFTT-AASMEQGQFNP-NELFNRVGGIQVGDV 376
E.faeciumDO_PBP2                    DAIWRNF--------LVQDSYEPGSTMKVFTT-AAAIEEGEFNE-NETFQ-SGKIQVEDA 369
                                                           .*  *       .

G.adiacensATCC49175_PBP1B           NGTYWQPTNYGNTITN--QFLTVRYALFRSFNNPVIKIYQTMLQK--------------- 535
G.adiacensGA01_PBP1B                NGTYWQPTNYGNTITN--QFLTVRYALFRSFNNPVIKIYQTMLQK--------------- 535
G.adiacensIS48_PBP1B                NGTYWQPTNYGNTITN--QFLTVRYALFRSFNNPVIKIYQTMLQK--------------- 535
G.adiacensKHUD009_PBP1B             NGTYWQPTNYGNTITN--QFLTVRYALFRSFNNPVIKIYQTMLQK--------------- 535
S.pneumoniaeD39_PBP1B               N----PIMYANSK-GT--GMMTLGEALNYSWNIPAYWTYRMLREN--------------- 531
S.pneumoniaeR6_PBP1B                N----PIMYANSK-GT--GMMTLGEALNYSWNIPAYWTYRMLREN--------------- 531
```

```
S.pneumoniaeNCTC7465_PBP1B       N----PIMYANSK-GT--GMMTLGEALNYSWNIPAYWTYRMLREN--------------- 531
S.pyogenesATCC-BAA-595_PBP1B     Q----KIMHADSE-GT--AMMPLQEALNTSWNIPAFWTQKLLREK--------------- 506
E.faeciumDO_PBP1B                ENAGEKIVNATNEGSN--TFQTVRESLEWSNNIPAYHLYQDVLNS--------------- 508
E.faecalisOG1RF_PBP1B            ----TEFVNSTNVDLN--QFVTVRNALNWSFNIPVVHVNNELRKK--------------- 522
G.adiacensATCC49175_PBP2A        K---YTPENWNKQ-YQ--GTVTMTEALNQSWNAPAVWLLDKIGLK--------------- 487
G.adiacensKHUD009_PBP2A          K---YTPENWNKQ-YQ--GTVTMTEALNQSWNAPAVWLLDKIGLK--------------- 487
G.adiacensGA01_PBP2A             K---YTPENWNKQ-YQ--GTVTMTEALNQSWNAPAVWLLDKIGLK--------------- 487
G.adiacensIS48_PBP2A             K---YTPENWNKQ-YQ--GTVTMTEALNQSWNAPAVWLLDKIGLK--------------- 487
E.faecalisOG1RF_PBP2A            ----YPAQNYSRT-YS--GEVPMYQALGESLNLPAVWLLHQIGLD--------------- 469
E.faeciumDO_PBP2A                ----YDAHNFDGT-YQ--GEVPMYQAVAQSLNLPTVWLLHEIGLQ--------------- 467
S.pyogenesATCC-BAA-595_PBP2A     G---YQPHNYGNY-ES--EDVPMYQALANSYNIPAVSTLNDIGID--------------- 527
S.pneumoniaeR6_PBP2A             S---YKVDNYAGIKTS--REVPMYQSLAESLNLPAVATVNDLGVD--------------- 480
S.pneumoniaeD39_PBP2A            S---YKVDNYAGIKTS--REVPMYQSLAESLNLPAVATVNDLGVD--------------- 480
S.pneumoniaeNCTC7465_PBP2A       S---YKVDNYAGIKTS--REVPMYQALAESLNLPAVATVNDLGVD--------------- 480
G.adiacensKHUD009_PBP1A          G---VELNNWDFR-HN--GPMTLRRALVYSRNTTALEAFKAVGET--------------- 464
G.adiacensIS48_PBP1A             G---VELNNWDFR-HN--GPMTLRRALVYSRNTTALEAFKAVGET--------------- 464
G.adiacensATCC49175_PBP1A        G---VELNNWDFR-HN--GPMTLRRALVYSRNTTALEAFKAVGET--------------- 464
G.adiacensGA01_PBP1A             G---VELNNWDFR-HN--GPMTLRRALVYSRNTTALEAFKAVGET--------------- 464
E.faecalisOG1RF_PBP1A            D---IDVFNSDLT-YQ--GVITMRRAIMGSRNTTAVQTFDEVGKE--------------- 469
E.faeciumDO_PBP1A                S---TPVGNWDNQ-YM--GTITLRQALYLSRNVPAVKLFNEVGSD--------------- 473
S.pyogenes_ATCC-BAA-595_PBP1A    T---TQLFNWDLR-YN--GWMTIQAAIMLSRNVPAVRALEAAGLD--------------- 446
S.pneumoniaeNCTC7465_PBP1A       N---TPVYNWDRG-YF--GNITLQYALQQSRNVPAVETLNKVGLN--------------- 443
S.pneumoniaeR6_PBP1A             N---TPVYNWDRG-YF--GNITLQYALQQSRNVPAVETLNKVGLN--------------- 443
S.pneumoniaeD39_PBP1A            N---TPVYNWDRG-YF--GNITLQYALQQSRNVPAVETLNKVGLN--------------- 443
G.adiacensIS48_PBP2B             K----PKSSWFNRNGQIE--LTDLDALERSSNVYMIKLAMKMGGQAEYVKG--GKLNINL 477
G.adiacensGA01_PBP2B             K----PKSSWFNRNGQIE--LTDLDALERSSNVYMIKLAMKMGGQAEYVKG--GKLNINL 477
G.adiacensATCC49175_PBP2B        K----PKSSWFNRNGQIE--LTDLDALERSSNVYMIKLAMKMGGQAEYVKG--GKLNINL 477
G.adiacensKHUD009_PBP2B          K----PKSSWFNRNGQIE--LTDLDALERSSNVYMIKLAMKMGGQAEYVKG--GKLNINL 477
S.pneumoniaeR6_PBP2B             A----PINSWYTQAYG-SFPITAVQALEYSSNTYMVQTALGLMGQTYQPNMFVGTSN-LE 477
S.pneumoniaeD39_PBP2B            A----PINSWYTQAYG-SFPITAVQALEYSSNTYMVQTALGLMGQTYQPNMFVGTSN-LE 477
S.pneumoniaeNCTC7465_PBP2B       A----PINSWYTQAYG-SFPITAVQALEYSSNTYMVQTALGLMGQTYQPNMFVGTSN-LE 472
E.faecalisOG1RF_PBP2B            N----PKASWWNASGGTTMQLTAEQALEYSSNAYMMKLVFKMMGVNYYPNMIFPYEVGDD 494
E.faeciumDO_PBP2B                S----EKSSVFNRSGQVA--LDAVKALELSSNTYMIKVALKMLGLDYTPGMGLPSLDEEA 494
E.faecalisOG1RF_PBP4             K----SWGGYFATRVKEASPVNLRTALVNSDNIYFAQQTLRMGED--------------- 497
E.faeciumDO_PBP5                 S----SWGSYQVTRVSDVSQVDLKTALIYSDNIYMAQETLKMGEK--------------- 495
S.pyogenesATCC-BAA-595_PBP2       T----IQDWSINEGISTGQYMNYAQGFAFSSNVGMTKLEQKMGNA--------------- 414
S.pneumoniaeR6_PBP2              T----IRDWDVNEGLTGGRMMTFSQGFAHSSNVGMTLLEQKMGDA--------------- 410
S.pneumoniaeD39_PBP2             T----IRDWDVNEGLTGGRMMTFSQGFAHSSNVGMTLLEQKMGDA--------------- 410
S.pneumoniaeNCTC7465_PBP2        T----IRDWDVNEGLTGGRMMTFSQGFAHSSNVGMTLLEQKMGDA--------------- 410
G.adiacensATCC49175_PBP2         L----VRD--YNK--VGWGNITYLEGLAHSSNVAFVHVIQKIGAE--------------- 392
G.adiacensIS48_PBP2              L----VRD--YNK--VGWGNITYLEGLAHSSNVAFVHVIQKIGVE--------------- 392
G.adiacensGA01_PBP2              L----VRD--YNK--VGWGNITYLEGLAHSSNVAFVHVIQKIGVE--------------- 392
G.adiacensKHUD009_PBP2           L----VRD--YNK--VGWGNITYLEGLAHSSNVAFVHVIQKIGVE--------------- 392
```

```
E.faecalisOG1RF_PBP2               T----VNDHDYTR-LNGKEYLNYRQAISWSSNIGMVKLEQKMGDE--------------- 416
E.faeciumDO_PBP2                   T----INDHDFGE----KGVLTMRQALSWSSNVGMVILEQRLGG---------------- 405
                                                             ..  * *

G.adiacensATCC49175_PBP1B          ----GI----NAGEYLKKMGIKGITEDE--------------------YQNIAL---SIG 564
G.adiacensGA01_PBP1B               ----GI----NAGEYLKKMGIKGITEDE--------------------YQNIAL---SIG 564
G.adiacensIS48_PBP1B               ----GI----NAGEYLKKMGIKGITEDE--------------------YQNIAL---SIG 564
G.adiacensKHUD009_PBP1B            ----GI----NAGEYLKKMGIKGITEDE--------------------YQNIAL---SIG 564
S.pneumoniaeD39_PBP1B              ----GV----DVKGYMEKMGYEIP---E--------------------YGIESL---PMG 557
S.pneumoniaeR6_PBP1B               ----GV----DVKGYMEKMGYEIP---E--------------------YGIESL---PMG 557
S.pneumoniaeNCTC7465_PBP1B         ----GV----DVKGYMEKMGYEIP---E--------------------YGIESL---PMG 557
S.pyogenesATCC-BAA-595_PBP1B       ----GV----DVENYMTKMGYKIA---D--------------------YSIESL---PLG 532
E.faeciumDO_PBP1B                  ----GGSKQYAYEHYLAKMNYPAND--N--------------------WGVESA---PLG 539
E.faecalisOG1RF_PBP1B              ----MGDDNFSYNHYLSKMNYPASD--A--------------------WAYESA---PLG 553
G.adiacensATCC49175_PBP2A          ----KG------IEKVHQFGIETDPGDE--------------------YLGIAL-----G 512
G.adiacensKHUD009_PBP2A            ----KG------IEKVHQFGIETDPGDE--------------------YLGIAL-----G 512
G.adiacensGA01_PBP2A               ----KG------IEKVHQFGIETDPGDE--------------------YLGIAL-----G 512
G.adiacensIS48_PBP2A               ----KG------IEKVHQFGIETDPGDE--------------------YLGIAL-----G 512
E.faecalisOG1RF_PBP2A              ----KG------YEKTEKFGIPLSEKDR--------------------YYGLAL-----G 494
E.faeciumDO_PBP2A                  ----KG------YDKAEEFGLPLAKSDK--------------------YYGLAL-----G 492
S.pyogenesATCC-BAA-595_PBP2A       ----KA------FTYGKTFGLDMSSAKK--------------------ELGVAL-----G 552
S.pneumoniaeR6_PBP2A               ----KA------FEAGEKFGLNMEKVDR--------------------VLGVAL-----G 505
S.pneumoniaeD39_PBP2A              ----KA------FEAGEKFGLNMEKVDR--------------------VLGVAL-----G 505
S.pneumoniaeNCTC7465_PBP2A         ----KA------FEAGEKFGLNMEKVDR--------------------VLGVAL-----G 505
G.adiacensKHUD009_PBP1A            ----NI------KSFLNNLDIQIKNDGQ--------------------DYLVESNAI--- 491
G.adiacensIS48_PBP1A               ----NI------KSFLNNLDIQIKNDGQ--------------------DYLVESNAI--- 491
G.adiacensATCC49175_PBP1A          ----NI------KSFLKNLDIQIKNDGQ--------------------DYLVESNAI--- 491
G.adiacensGA01_PBP1A               ----NI------KSFLNNLDIQIKNDGQ--------------------DYLVESNAI--- 491
E.faecalisOG1RF_PBP1A              ----NI------MPFIKGLGIDYKNLEA--------------------SNAISSNTSDVD 499
E.faeciumDO_PBP1A                  ----KV------ASFLKNLGIEYSTIHQ--------------------SNAISSNTEEQD 503
S.pyogenes_ATCC-BAA-595_PBP1A      ----YA------RSFLSSLGINYPEMHY--------------------SNAISSNNSSSD 476
S.pneumoniaeNCTC7465_PBP1A         ----RA------KTFLNGLGIDYPSIHY--------------------SNAISSNTTESD 473
S.pneumoniaeR6_PBP1A               ----RA------KTFLNGLGIDYPSIHY--------------------SNAISSNTTESD 473
S.pneumoniaeD39_PBP1A              ----RA------KTFLNGLGIDYPSIHY--------------------SNAISSNTTESD 473
G.adiacensIS48_PBP2B               SLFDKLREYYAQFGLGVRTGIDLPNEGKGYNGGT--------------ANAFSALDFAFG 523
G.adiacensGA01_PBP2B               SLFDKLREYYAQFGLGVRTGIDLPNEGKGYNGGT--------------ADAFSALDFAFG 523
G.adiacensATCC49175_PBP2B          SLFDKLREYYAQFGLGVRTGIDLPNEGKGYNGGT--------------ADAFSALDFAFG 523
G.adiacensKHUD009_PBP2B            SLFDKLREYYAQFGLGVRTGIDLPNEGKGYNGGT--------------ADAFSALDFAFG 523
S.pneumoniaeR6_PBP2B               SAMEKLRSTFGEYGLGTATGIDLPDESTGFVPKE--------------YSFANYITNAFG 523
S.pneumoniaeD39_PBP2B              SAMEKLRSTFGEYGLGTATGIDLPDESTGFVPKE--------------YSFANYITNAFG 523
S.pneumoniaeNCTC7465_PBP2B         SAMEKLRSTFGEYGLGTATGIDLPDESTGFVPKE--------------YSFANYITNAFG 518
E.faecalisOG1RF_PBP2B              TVFKELRKAFAEYGMGTKTGIDIPGETTGIQNKD-FK------DSSSAPQGGNLLDLSFG 547
E.faeciumDO_PBP2B                  KAYQQLRDSFKEFGLGTTTGIDLPNESPGISRSVDYMKKFNADNGKEWYTPGNFTDLAFG 554
```

```
E.faecalisOG1RF_PBP4              ----KFRAGLNKFIFGEELDLPIAMTPAQISNEDKF------------NSEILLADTGYG 541
E.faeciumDO_PBP5                  ----KFRTGLDKFIFGEDLDLPISMNPAQISNEDSF------------NSDILLADTGYG 539
S.pyogenesATCC-BAA-595_PBP2       ----KWMNYLTKFRFGFPTRFGLKDEDA---GIFPS------------DNIVTQAMSAFG 455
S.pneumoniaeR6_PBP2               ----TWLDYLNRFKFGVPTRFGLTDEYA---GQLPA------------DNIVNIAQSSFG 451
S.pneumoniaeD39_PBP2              ----TWLDYLNRFKFGVPTRFGLTDEYA---GQLPA------------DNIVNIAQSSFG 451
S.pneumoniaeNCTC7465_PBP2         ----TWLDYLNRFKFGVPTRFGLTDEYA---GQLPA------------DNIVNIAQSSFG 451
G.adiacensATCC49175_PBP2          ----KWKQYLEAFGFSKSTNSGFANEIS---GSNPF------------NSYLQQLSTGFG 433
G.adiacensIS48_PBP2               ----KWKQYLEAFGFSKSTNSGFANEIS---GSNPF------------NSYLQQLSTGFG 433
G.adiacensGA01_PBP2               ----KWKQYLEAFGFSKSTNSGFANEIS---GSNPF------------NSYLQQLSTGFG 433
G.adiacensKHUD009_PBP2            ----KWKQYLEAFGFSKSTNSGFANEIS---GSNPF------------NSYLQQLSTGFG 433
E.faecalisOG1RF_PBP2              ----KWMEYLKKFGFGTSTHSGLSGESA---GKLPG------------TNFVDRAMSAFG 457
E.faeciumDO_PBP2                  ----RWYNYLQKLGFGQSTHSGLDDEVN---GALPT------------SNIVDRAMSAYG 446


G.adiacensATCC49175_PBP1B         GTRTGPTVLEQTSAFSTLANGGEHHDAYLIEKIEDSRGNIVYQHE-D------KKERVFS 617
G.adiacensGA01_PBP1B              GTRTGPTVLEQTSAFSTLANGGEHHDAYLIEKIEDSRGNIVYQHE-D------KKERVFS 617
G.adiacensIS48_PBP1B              GTRTGPTVLEQTSAFSTLANGGEHHDAYLIEKIEDSRGNVVYQHE-D------KKERVFS 617
G.adiacensKHUD009_PBP1B           GTRTGPTVLEQTSAFSTLANGGEHHDAYLIEKIEDSRGNVVYQHE-D------KKERVFS 617
S.pneumoniaeD39_PBP1B             GG-IEVTVAQHTNGYQTLANNGVYHQKHVISKIEAADGRVVYEYQ-D------KPVQVYS 609
S.pneumoniaeR6_PBP1B              GG-IEVTVAQHTNGYQTLANNGVYHQKHVISKIEAADGRVVYEYQ-D------KPVQVYS 609
S.pneumoniaeNCTC7465_PBP1B        GG-IEVTVAQHTNGYQTLANNGVYHQKHVISKIEAADGRVVYEYQ-D------KPVQVYS 609
S.pyogenesATCC-BAA-595_PBP1B       GG-IEVSVAQQTNAYQMLSNNGLYQKQYIVDKITASDGTVVYKHE-N------KPIRIFS 584
E.faeciumDO_PBP1B                 -T-VDVTTLQQTNGFQALANGGVYEEGYIIDSITDNAGNVIYKHE-S------NSVRIYS 590
E.faecalisOG1RF_PBP1B             -S-VETNVVTQTNGFQALANKGKYQKAYMIEKITDNSGHVVYEHK-D------EGTQVYS 604
G.adiacensATCC49175_PBP2A         GLTKGVSPIQMASAYTAFANKGVRTKPRFVTKIVDANGKVIVDNTAV------KDNRVTT 566
G.adiacensKHUD009_PBP2A           GLTKGVSPIQMASAYTAFANKGVRTKPRFVTKIVDANGKVIVDNTAV------KDNRVTT 566
G.adiacensGA01_PBP2A              GLTKGVSPIQMASAYTAFANKGVRTKPRFVTKIVDANGKVIVDNTAV------KDNRVTT 566
G.adiacensIS48_PBP2A              GLTKGVSPIQMASAYTAFANKGVRTKPRFVTKIVDANGKVIVDNTAV------KDNRVTT 566
E.faecalisOG1RF_PBP2A             GLQTGVSPLTMAGAYSAFANEGYKTETHLITKIVDSTGAIVVDNTKV------KKEQVIT 548
E.faeciumDO_PBP2A                 GLEKGVSPLIMAGAYSAFANDGQMYTPHLITKIVDSTGAVIVDNTNK------KPKQVIS 546
S.pyogenesATCC-BAA-595_PBP2A       GSV-TTNPLEMAQAYAAFANNGVIHPAHLINRIENARGEVLKTFT-D------KAKRVVS 604
S.pneumoniaeR6_PBP2A              SGV-ETNPLQMAQAYAAFANEGLMPEAHFISRIENASGQVIASHK-N------SQKRVID 557
S.pneumoniaeD39_PBP2A             SGV-ETNPLQMAQAYAAFANEGLMPEAHFISRIENASGQVIASHK-N------SQKRVID 557
S.pneumoniaeNCTC7465_PBP2A        SGV-ETNPLQMAQAYAAFANEGLMPEAHFISRIENASGQVIANHK-N------SQKRVID 557
G.adiacensKHUD009_PBP1A           --GAEISPIKMAAAYATFSNAGTYSKPYTVTKITTRDGQVYEF--KP------EQKQAMK 541
G.adiacensIS48_PBP1A              --GAEISPIKMAAAYATFSNAGTYSKPYTVTKITTRDGQVYEF--KP------EQKQAMK 541
G.adiacensATCC49175_PBP1A         --GAEISPIKMAAAYATFSNAGTYSKPYTVTKITTRDGQVYEF--KP------EQKQAMK 541
G.adiacensGA01_PBP1A              --GAEISPIKMAAAYATFSNAGTYSKPYTVTKITTRDGQVYEF--KP------EQKQAMK 541
E.faecalisOG1RF_PBP1A             GDKYGISSLKLAAAYAAFANNGIYNKPYYVNKVVFNDGTSVDY--QP------DGKRAMK 551
E.faeciumDO_PBP1A                 GTKYGASSLKMAAAYAAFANGGTYYKPQYVNKIVFQDGTEETY--EP------DGKTAMS 555
S.pyogenes_ATCC-BAA-595_PBP1A     -KKYGASSEKMAAAYAAFANGGIYHKPRYVNKVEFSDGTSKTF--DE------KGKRAMK 527
S.pneumoniaeNCTC7465_PBP1A        -KKYGASSEKMAAAYAAFANGGTYYKPMYIHKVVFSDGSEKEF--SN------VGTRAMK 524
S.pneumoniaeR6_PBP1A              -KKYGASSEKMAAAYAAFANGGTYYKPMYIHKVVFSDGSEKEF--SN------VGTRAMK 524
```

```
S.pneumoniaeD39_PBP1A              -KKYGASSEKMAAAYAAFANGGTYYKPMYIHKVVFSDGSEKEF--SN------VGTRAMK 524
G.adiacensIS48_PBP2B               QFDL-YTPLQLAQYMSTIANGGTRIAPRLVKEIHETSPKGGIGNLEDVIPTKIMNTLQVE 582
G.adiacensGA01_PBP2B               QFDL-YTPLQLAQYMSTIANGGTRIAPRLVKEIHETSPKGGIGNLEDVIPTKIMNTLQVE 582
G.adiacensATCC49175_PBP2B          QFDL-YTPLQLAQYMSTIANGGTRIAPRLVKEIHETSPKGGIGNLEDVIPTKIMNTLQVE 582
G.adiacensKHUD009_PBP2B            QFDL-YTPLQLAQYMSTIANGGTRIAPRLVKEIHETSPKGGIGNLEDVIPTKIMNTLQVE 582
S.pneumoniaeR6_PBP2B               QFDN-YTPMQLAQYVATIANNGVRVAPRIVEGIYGNNDKGGLGDLIQQLQPTEMNKVNIS 582
S.pneumoniaeD39_PBP2B              QFDN-YTPMQLAQYVATIANNGVRVAPRIVEGIYGNNDKGGLGDLIQQLQPTEMNKVNIS 582
S.pneumoniaeNCTC7465_PBP2B         QFDN-YTPMQLAQYVATIANNGVRVAPRIVEGIYGNNDKGGLGDLIQQLQPTEMNKVNIS 577
E.faecalisOG1RF_PBP2B              QYDT-YSALQLAQYVSTVANNGIRVQPHVVEGIYGNDENGALGKILKEIEPKVLNKVNIS 606
E.faeciumDO_PBP2B                  QFDT-YTPIQLAQYASTVANGGKRVQPHLVKAIYGNDENGNLGEVKKEIGTTVENTVNIS 613
E.faecalisOG1RF_PBP4               QGQLLISPIQQATMYSVFQNNGTLVYPKLVLD-------KETKKKD----------NVIS 584
E.faeciumDO_PBP5                   QGELLINPIQQAAMYSVFANNGTLVYPKLIAD-------KETKDKK----------NVIG 582
S.pyogenesATCC-BAA-595_PBP2         QGIS-VTQIQMLRAFTAISNNGEMLEPQFISQIYDPNTASFRTANKE------IVGKPVS 508
S.pneumoniaeR6_PBP2                QGIS-VTQTQMIRAFTAIANDGVMLEPKFISAIYDPNDQTARKSQKE------IVGNPVS 504
S.pneumoniaeD39_PBP2               QGIS-VTQTQMIRAFTAIANDGVMLEPKFISAIYDPNDQTARKSQKE------IVGNPVS 504
S.pneumoniaeNCTC7465_PBP2          QGIS-VTQTQMIRAFTAIANDGVMLEPKFISAIYDPNDQTARKSQKE------IVGNPVS 504
G.adiacensATCC49175_PBP2           QGIT-VTVYQMMQAFTAIANGGQMQKLRLVDHFTEPNTDKETPNPVN------SLGKVIS 486
G.adiacensIS48_PBP2                QGIT-VTVYQMMQAFTAIANGGQMQKLRLVDHFTDPNTDKETPNPVN------SLGKVIS 486
G.adiacensGA01_PBP2                QGIT-VTVYQMMQAFTAIANGGQMQKLRLVDHFTDPNTDKETPNPVN------SLGKVIS 486
G.adiacensKHUD009_PBP2             QGIT-VTVYQMMQAFTAIANGGQMQKLRLVDHFTDPNTDKETPNPVN------SLGKVIS 486
E.faecalisOG1RF_PBP2               QAIT-VTNFQMMKGFSAIANDGSMLQPHYISKIVDKNTGKETITEPQ------IVGTPIK 510
E.faeciumDO_PBP2                   QAVG-VTNFQMMKAFTSIANNGTMIQPRYISKVVDPQTGEERTTQTE------VLGQPFS 499
                                         .          . * *       :

G.adiacensATCC49175_PBP1B          EATSYLTTNMLQDIAN---STQFYNMKGNMG------FSSDLAGKTGTSENE-------- 660
G.adiacensGA01_PBP1B              EATSYLTTNMLQDIAN---STQFYNMKGNMG------FSSDLAGKTGTSENE-------- 660
G.adiacensIS48_PBP1B              DATAYLTTNMLQDIAN---STQFYNMKGNMG------FSSDLAGKTGASENE-------- 660
G.adiacensKHUD009_PBP1B            DATAYLTTNMLQDIAN---STQFYNMKGNMG------FSSDLAGKTGTSENE-------- 660
S.pneumoniaeD39_PBP1B              KATATIMQGLLREVLS---SRVTTTFKSNLTSLNPTLANADWIGKTGTTNQD-------- 658
S.pneumoniaeR6_PBP1B               KATATIMQGLLREVLS---SRVTTTFKSNLTSLNPTLANADWIGKTGTTNQD-------- 658
S.pneumoniaeNCTC7465_PBP1B         KATATIMQGLLREVLS---SRVTTTFKSNLTSLNPTLANADWIGKTGTTNQD-------- 658
S.pyogenesATCC-BAA-595_PBP1B        AATATILQELLRGPIT---SGATTTFKNRLAAINPWLANADWIGKTGTTENY-------- 633
E.faeciumDO_PBP1B                  EATASIMNDMMRSVIN---AKITTPFKDAISSLNGNLGKADWVGKTGSTNEY-------- 639
E.faecalisOG1RF_PBP1B              PATASIMNDLLRSVVD---SANTTKFKPTLAGLNPHLASADWVGKTGTTNDY-------- 653
G.adiacensATCC49175_PBP2A          EEVAKKMTSMLLTVYGQ--EG---------LGAPFSPSGKIIAGKTGTTEAL-------- 607
G.adiacensKHUD009_PBP2A            EEVAKKMTSMLLTVYGQ--EG---------LGAPFSPSGKIIAGKTGTTEAL-------- 607
G.adiacensGA01_PBP2A              EEVAKKMTSMLLTVYGQ--EG---------LGAPFSPSGKIIAGKTGTTEAL-------- 607
G.adiacensIS48_PBP2A              EEVAKKMTSMLLTVYGQ--EG---------LGAPFSPSGKIIAGKTGTTEAL-------- 607
E.faecalisOG1RF_PBP2A              KDVADGITSMLLGVFS---SG---------SGVNAQPAGYVMAGKTGTTETN-------- 588
E.faeciumDO_PBP2A                  KETADEMTSMLLGTFS---NG---------TAAAANPAGYTIAGKTGTTETN-------- 586
S.pyogenesATCC-BAA-595_PBP2A        QSVADKMTAMMLGTFS---NG---------TAVNANVYGYTLAGKTGTTETN-------- 644
S.pneumoniaeR6_PBP2A               KSVADKMTSMMLGTFT---NG---------TGISSSPADYVMAGKTGTTEAV-------- 597
S.pneumoniaeD39_PBP2A              KSVADKMTSMMLGTFT---NG---------TGISSSPADYVMAGKTGTTEAV-------- 597
```

```
S.pneumoniaeNCTC7465_PBP2A         KSVADKMTSMMLGTFT---NG---------TGISSSPADYVMAGKTGTTEAV-------- 597
G.adiacensKHUD009_PBP1A            DSTAYMITNVLKDSFT---YG---------FATEVAIPGLSTAAKTGSSNYTIEQKRAMG 589
G.adiacensIS48_PBP1A               DSTAYMITNVLKDSFT---YG---------FATEVAIPGLSTAAKTGSSNYNIEQKRAMG 589
G.adiacensATCC49175_PBP1A          DSTAYMITNVLKDSFT---YG---------FATEVAIPGLSTAAKTGSSNYTIEQKRAMG 589
G.adiacensGA01_PBP1A               DSTAYMITNVLKDSFT---YG---------FATEVAIPGLSTAAKTGSSNYTIEQKRAMG 589
E.faecalisOG1RF_PBP1A              DSTAYMMTDMLKDVLN---GG---------TGFNGAIPGLIQAAKTGTSNYTDEDLARMG 599
E.faeciumDO_PBP1A                  PETAYMITDILKDTIT---EG---------TGTNAQIAGLYQAGKTGTSNYTDDEYAKLG 603
S.pyogenes_ATCC-BAA-595_PBP1A      ETTAYMMTDMLKTVLT---YG---------TGTAAAIPGVAQAGKTGTSNYTDEELAKIG 575
S.pneumoniaeNCTC7465_PBP1A         ETTAYMMTDMMKTVLT---YG---------TGRNAYLAWLPQAGKTGTSNYTDEEIENHI 572
S.pneumoniaeR6_PBP1A               ETTAYMMTDMMKTVLS---YG---------TGRNAYLAWLPQAGKTGTSNYTDEEIENHI 572
S.pneumoniaeD39_PBP1A              ETTAYMMTDMMKTVLS---YG---------TGRNAYLAWLPQAGKTGTSNYTDEEIENHI 572
G.adiacensIS48_PBP2B               KKILDHIKEGLYRVTHG-ENGTS------AS-TFKNY-SPEVAGKTGTVEAFYSGPNPAY 633
G.adiacensGA01_PBP2B               KKILDHIKEGLYRVTHG-ENGTS------AS-TFKNY-SPEVAGKTGTAEAFYSGPNPAY 633
G.adiacensATCC49175_PBP2B          KKILDHIKEGLYRVTHG-ENGTS------AS-TFKNY-SPEVAGKTGTAEAFYSGPNPAY 633
G.adiacensKHUD009_PBP2B            KKILDHIKEGLYRVTHG-ENGTS------AS-TFKNY-SPEVAGKTGTTEAFYSGPNPAY 633
S.pneumoniaeR6_PBP2B               DSDMSILHQGFYQVAHG-TSGLT------TGRAFSNGALVSISGKTGTAESYVADG---- 631
S.pneumoniaeD39_PBP2B              DSDMSILHQGFYQVAHG-TSGLT------TGRAFSNGALVSISGKTGTAESYVADG---- 631
S.pneumoniaeNCTC7465_PBP2B         DSDMSILHQGFYQVAHG-TSELT------TGRAFSNGALVSISGKTGTAESYVADG---- 626
E.faecalisOG1RF_PBP2B              EDQIGILQQGFYNVVNG-TSPFT------TARGLKSD-KFSIAAKTGTAETQATDANGV- 657
E.faeciumDO_PBP2B                  AENMAILREGFHQVVHG-TDPYT------TAKPLASA-KMDLSAKTGTAETVAEGH--P- 662
E.faecalisOG1RF_PBP4               ANAANTIATDLLGSVED-PSGYV--YN-------MYNPNFSLAAKTGTAEIKDKQDTD-- 632
E.faeciumDO_PBP5                   ETAVQTIVPDLREVVQD-VNGTA--HS-------LSALGIPLAAKTGTAEIKEKQDEK-- 630
S.pyogenesATCC-BAA-595_PBP2         KKAASETRQYMIGVGTDPEFGTL--YSKTFG-PIIKVGDLPVAVKSGTAQIGSEDGSGYQ 565
S.pneumoniaeR6_PBP2                KDAASLTRTNMVLVGTDPVYGTM--YNHSTGKPTVTVPGQNVALKSGTAQIADEKNGGYL 562
S.pneumoniaeD39_PBP2               KDAASLTRTNMVLVGTDPVYGTM--YNHSTGKPTVTVPGQNVALKSGTAQIADEKNGGYL 562
S.pneumoniaeNCTC7465_PBP2          KDAASLTRTNMVLVGTDPVYGTM--YNHSTGKPTVTVPGQNVALKSGTAQIADEKNGGYL 562
G.adiacensATCC49175_PBP2           PEAAKKTLEYLYQATRM-KNGTA--YE-------FNIDGEEIAAKTGTAEIINPETGKYY 536
G.adiacensIS48_PBP2               PEAAKKTLEYLYQATRM-KNGTA--YE-------FNIDGEEIAAKTGTAEFINPETGKYY 536
G.adiacensGA01_PBP2               PEAAKKTLEYLYQATRM-KNGTA--YE-------FNIDGEEIAAKTGTAEFINPETGKYY 536
G.adiacensKHUD009_PBP2             PEAAKKTLEYLYQATRM-KNGTA--YE-------FNIDGEEIAAKTGTAEIINPETGKYY 536
E.faecalisOG1RF_PBP2               AQTAQQIRTYMIDTVEDPTYGIA--YD------IYKVPGYHVAAKTGTAQIS--DGKGYV 560
E.faeciumDO_PBP2                   KETTEKVREYMRDVVESENYGSA--YG------VYSVPGYNVSAKTGTAQIA-SDTGGYQ 550
                                             :                                 *:*: :

G.adiacensATCC49175_PBP1B          --------------IDNWFVAY----TPTVTLGSWIGYDNFYNAR-YAITAGDGYGEPTT 701
G.adiacensGA01_PBP1B               --------------IDNWFVAY----TPTVTLGSWIGYDNFYNAR-YAITAGDGYGEPTT 701
G.adiacensIS48_PBP1B               --------------IDNWFVAY----TPTVTLGSWIGYDNFYNAR-YAITAGDGYGEPTT 701
G.adiacensKHUD009_PBP1B            --------------IDNWFVAY----TPTVTLGSWIGYDNFYNAR-YAITAGDGYGEPTT 701
S.pneumoniaeD39_PBP1B              --------------ENMWLMLS----TPRLTLGGWIGHDDNHSL-----SRRAGY----S 691
S.pneumoniaeR6_PBP1B               --------------ENMWLMLS----TPRLTLGGWIGHDDNHSL-----SRRAGY----S 691
S.pneumoniaeNCTC7465_PBP1B         --------------ENMWLMLS----TPRLTLGGWIGHDDNHSL-----SRRAGY----S 691
S.pyogenesATCC-BAA-595_PBP1B        --------------TDVWLVLS----TPKVTLGGWAGHDDNTSL-----APLTGY----N 666
E.faeciumDO_PBP1B                  --------------RDSWLIVS----TPSITISSWAGHDDNTGM-----DSKARI----- 671
```

```
E.faecalisOG1RF_PBP1B              --------------KDSWLIVS----TPTVTLSSWSGHDLPAPM-----TTTSGD----- 685
G.adiacensATCC49175_PBP2A          --------NNENGSRDQWMIGY----TPDVVVATWMGYDQSGN-Y-SLSASSREGVGPL- 652
G.adiacensKHUD009_PBP2A            --------NNENGSRDQWMIGY----TPDVVVATWMGYDQSGN-Y-SLSASSREGVGPL- 652
G.adiacensGA01_PBP2A               --------NNENGSRDQWMIGY----TPDVVVATWMGYDQSGN-Y-SLSASSREGVGPL- 652
G.adiacensIS48_PBP2A               --------NNENGSRDQWMIGY----TPDVVVATWMGYDQSGN-Y-SLSASSREGVGPL- 652
E.faecalisOG1RF_PBP2A              --------FDSSKTNDQWVIGY----TPEVVIATWLGFQETSKTH-YLEGSSATYASQV- 634
E.faeciumDO_PBP2A                  --------FDATKANDQWMIGY----TPDVVISTWMGYEESSKLH-YLEGTSGNVVGKV- 632
S.pyogenesATCC-BAA-595_PBP2A       --------FNPDLAGDQWVIGY----TPDVVISQWVGFNQTDENH-YLTDSSAGTASAI- 690
S.pneumoniaeR6_PBP2A               --------FNPEYTSDQWVIGY----TPDVVISHWLGFPTTDENH-YLAGSTSNGAAHV- 643
S.pneumoniaeD39_PBP2A              --------FNPEYTSDQWVIGY----TPDVVISHWLGFPTTDENH-YLAGSTSNGAAHV- 643
S.pneumoniaeNCTC7465_PBP2A         --------FNPEYTSDQWVIGY----TPDVVISHWLGFPTTDENH-YLAGSTSNGAAHV- 643
G.adiacensKHUD009_PBP1A            AS------DYEDIIPDSWFIGY----SPDYTISVWTGYDNPYEKGGGVDTTEQGYAKLI- 638
G.adiacensIS48_PBP1A               AS------DYEDIIPDSWFIGY----SPDYTISVWTGYDNPYEKGGGVDTTEQGYAKLI- 638
G.adiacensATCC49175_PBP1A          AS------DYEDIIPDSWFIGY----SPDYTISVWTGYDNPYEKGGGVDTTEQGYAKLI- 638
G.adiacensGA01_PBP1A               AS------DYEDIIPDSWFIGY----SPDYTISVWTGYDNPYEKGGGVDTTEQGYAKLI- 638
E.faecalisOG1RF_PBP1A              T-------TEKGIAPDSTFVGY----TTHYAVSVWTGYNDRNTPI---YQEYYGIASDV- 644
E.faeciumDO_PBP1A                  I-------S-SGVYPDILFAGY----TPNYSISVWTGYNKKMTPV---TSESSHVASDV- 647
S.pyogenes_ATCC-BAA-595_PBP1A      EKYGLYPDYVGTLAPDENFVGF----TKRYAMAVWTGYKNRLTPV---YGSSLEIASDV- 627
S.pneumoniaeNCTC7465_PBP1A         K-------TSQFVAPDELFAGY----TRKYSMAVWTGYSNRLTPL---VGNGLTVAAKV- 617
S.pneumoniaeR6_PBP1A               K-------TSQFVAPDELFAGY----TRKYSMAVWTGYSNRLTPL---VGNGLTVAAKV- 617
S.pneumoniaeD39_PBP1A              K-------TSQFVAPDELFAGY----TRKYSMAVWTGYSNRLTPL---VGNGLTVAAKV- 617
G.adiacensIS48_PBP2B               K---------NEAVENSTFISYAPYDNPEIVVSVVAPYFKDGLP----SDYAAKIAKKV- 679
G.adiacensGA01_PBP2B               K---------NEAVENSTFISYAPYDNPEIVVSVVAPYFKDGSP----SDYAAKIAKKV- 679
G.adiacensATCC49175_PBP2B          K---------NEAVENSTFISYAPYDNPEIVVSVVAPYFKDGSP----SDYAAKIAKKV- 679
G.adiacensKHUD009_PBP2B            K---------NEAVENSTFISYAPYDNPEIVVSVVAPYFKDGSP----SDYAAKIAKKV- 679
S.pneumoniaeR6_PBP2B               -----------QQATNTNAVAYAPSDNPQIAVAVVFPHNTNLT-----NGVGPSIARDI- 674
S.pneumoniaeD39_PBP2B              -----------QQATNTNAVAYAPSDNPQIAVAVVFPHNTNLT-----NGVGPSIARDI- 674
S.pneumoniaeNCTC7465_PBP2B         -----------QQATNTNAVAYAPSDNPQIAVAVVFPHNTNLT-----NGVGPSIARDI- 669
E.faecalisOG1RF_PBP2B              ----------NHTTVNSNLVAYAPYENPEIAISVVLPHLNDES-----SKPNQTIAKEV- 701
E.faeciumDO_PBP2B                  ----------DITTVNSNIVAYGPTDNPEIAISVVLPNLLDEQ-----DHMNLTIAKEI- 706
E.faecalisOG1RF_PBP4               -G--------KENSFLLT---L-DRSNNKFLTMIMVENSGEN-------GSATDISKPL- 671
E.faeciumDO_PBP5                   -G--------KENSFLFA---F-NPDNQGYMMVSMLENKEDD-------DSATKRASEL- 669
S.pyogenesATCC-BAA-595_PBP2        DG--------GLTNYVYSVVAMVPADKPDFLMYVTMTKPQHFGP-----LFWQDVVNPV- 611
S.pneumoniaeR6_PBP2                V---------GLTDYIFSAVSMSPAENPDFILYVTVQQPEHYSG-----IQLGEFANPI- 607
S.pneumoniaeD39_PBP2               V---------GLTDYIFSAVSMSPAENPDFILYVTVQQPEHYSG-----IQLGEFANPI- 607
S.pneumoniaeNCTC7465_PBP2          V---------GVTDYIFSAVSMSPAENPDFILYVTVQQPEHYSG-----IQLGEFANPI- 607
G.adiacensATCC49175_PBP2           S---------NGNNYIFSVVGFAPYNDPKYILYVTVKQPRVAVTG---NTILKEIFNPL- 583
G.adiacensIS48_PBP2                S---------NGNNYIFSVVGFAPYNDPKYILYVTVKQPRVAVTV---NTILKEIFNPL- 583
G.adiacensGA01_PBP2                S---------NGNNYIFSVVGFAPYNDPKYILYVTVKQPRVAVTV---NTILKEIFNPL- 583
G.adiacensKHUD009_PBP2             S---------NGNNYIFSVVGFAPYNDPKYILYVTVKQPRVAVTG---NTILKEIFNPL- 583
E.faecalisOG1RF_PBP2               D---------GASQVLSSVVEMVPADNPEYVLYITLRQAKSGSP----SEAMAAIANPL- 606
E.faeciumDO_PBP2                   T---------GDTAYLYSIVEMVPSEDPDYVLYLTMKHPKTY-D----RMALAKIANPL- 595
```

```
G.adiacensATCC49175_PBP1B        RSQRQWTYLMKAAYEANPEL-------------IGKETTFKQPDSVYRDSVVSTTGTK-- 746
G.adiacensGA01_PBP1B             RSQRQWTYLMKAAYEANPEL-------------IGKETTFKQPDSVYRDSVVSTTGTK-- 746
G.adiacensIS48_PBP1B             RSQRQWTYLMKAAYEANPEL-------------IGKETTFKQPDSVYRDSVVSTTGTK-- 746
G.adiacensKHUD009_PBP1B          RSQRQWTYLMKAAYEANPEL-------------IGKETTFKQPDSVYRDSVVSTTGTK-- 746
S.pneumoniaeD39_PBP1B            NNSNYMAHLVNAIQQASPSI-------------WG-NERFALDPSVVKSEVLKSTGQK-- 735
S.pneumoniaeR6_PBP1B             NNSNYMAHLVNAIQQASPSI-------------WG-NERFALDPSVVKSEVLKSTGQK-- 735
S.pneumoniaeNCTC7465_PBP1B       NNSNYMAHLVNAIQQASPSI-------------WG-NERFALDPSVVKSEVLKSTGQK-- 735
S.pyogenesATCC-BAA-595_PBP1B     NNSNYLAYLANAINQADPNV-------------IGVGQRFNLDPGVIKANVLKSTGLQ-- 711
E.faeciumDO_PBP1B                RSANYLANLINQAYQAKPDI-------------FGTDEKFELSSDVMKKKVSGFTGQT-- 716
E.faecalisOG1RF_PBP1B            NNGNYMANLANALYYANPEL-------------FGIGQKFELDPSVIKSKVSEFTGEK-- 730
G.adiacensATCC49175_PBP2A        -----FKLEMEGLLQ------------------YTANTAFNVEPVKTKQSNQ-------- 681
G.adiacensKHUD009_PBP2A          -----FKLEMEGLLQ------------------YTANTAFNVEPVKTKQANQ-------- 681
G.adiacensGA01_PBP2A             -----FKLEMEGLLQ------------------YTANTAFNVEPVKTKQANQ-------- 681
G.adiacensIS48_PBP2A             -----FKLEMEGLLQ------------------YTANTAFNVEPVKTKQANQ-------- 681
E.faecalisOG1RF_PBP2A            -----FNSQASGILP------------------QVKQAQFPVADAYATGGKVVDSND--- 668
E.faeciumDO_PBP2A                -----FKSAAEGILP------------------YTSKTGFSVADAYATGGQVVAADQVGN 669
S.pyogenesATCC-BAA-595_PBP2A     -----FSTQASYILP------------------YTKGSQFHVDNAYAQNGISAVYGVNE- 726
S.pneumoniaeR6_PBP2A             -----FRNIANTILP------------------YTPGSTFTVENAYKQNGIAPANTKRQ- 679
S.pneumoniaeD39_PBP2A            -----FRNIANTILP------------------YTPGSTFTVENAYKQNGIAPANTKRQ- 679
S.pneumoniaeNCTC7465_PBP2A       -----FRNIANTILP------------------YTPGSTFTVENAYKQNGIAPANTKRQ- 679
G.adiacensKHUD009_PBP1A          -----YYHLMKYMAQ------------------YSSGEDWVQPESVVQQQIEVGSIPLSL 675
G.adiacensIS48_PBP1A             -----YYHLMKYMAQ------------------YSSGEDWVQPDSVVQQQIEVGSIPLSL 675
G.adiacensATCC49175_PBP1A        -----YYHLMKYMAQ------------------YSSGEDWVQPDSVVQQQIEVGSIPLSL 675
G.adiacensGA01_PBP1A             -----YYHLMKYMAQ------------------YSSGEDWVQPDSVVQQQIEVGSIPLSL 675
E.faecalisOG1RF_PBP1A            -----YREIMSYLSQ------------------NVSNDDWVQPDSVVRVGNELYVKDA-Y 680
E.faeciumDO_PBP1A                -----YRELMQYVSA------------------NVTNTDWEMPSGLIRVGGELYYKDQ-Y 683
S.pyogenes_ATCC-BAA-595_PBP1A    -----YRSMMTYLTN------------------GY-SEDWTMPNGLYRSGGFLYLSGT-Y 662
S.pneumoniaeNCTC7465_PBP1A       -----YRSMMTYLSE------------------GSNPEDWNIPEGLYRNGEFVFKNGA-R 653
S.pneumoniaeR6_PBP1A             -----YRSMMTYLSE------------------GSNPEDWNIPEGLYRNGEFVFKNGA-R 653
S.pneumoniaeD39_PBP1A            -----YRSMMTYLSE------------------GSNPEDWNIPEGLYRNGEFVFKNGA-R 653
G.adiacensIS48_PBP2B             ---------YEAYFGKKSNSETNAATVNTQVQQNNRQ----------------------- 707
G.adiacensGA01_PBP2B             ---------YEAYFGKKSNSETNAATVNTQVQQNNRQ----------------------- 707
G.adiacensATCC49175_PBP2B        ---------YEAYFGKKSNSETNAATVNTQVQQNNRQ----------------------- 707
G.adiacensKHUD009_PBP2B          ---------YEAYFGKKSNSETNAATVNTQVQQNNRQ----------------------- 707
S.pneumoniaeR6_PBP2B             ---------INLYQKYHPMN---------------------------------------- 685
S.pneumoniaeD39_PBP2B            ---------INLYQKYHPMN---------------------------------------- 685
S.pneumoniaeNCTC7465_PBP2B       ---------INLYQKYHPMN---------------------------------------- 680
E.faecalisOG1RF_PBP2B            ---------LEAYMEMYKK----------------------------------------- 711
E.faeciumDO_PBP2B                ---------MDAYYDMFMAK---------------------------------------- 717
E.faecalisOG1RF_PBP4             ---------IDYLE-------ATI---------------------K-------------- 680
E.faeciumDO_PBP5                 ---------LQYLN-------QNY---------------------Q-------------- 678
S.pyogenesATCC-BAA-595_PBP2      ---------LEEAYLMQDTLTKPV------VSDANRQTTYKLPNFVGKNPGETSSELR-- 654
```

```
S.pneumoniaeR6_PBP2              ---------LERASAMKDSLNLQTTAK--ALEQVSQQSPYPMPSVKDISPGDLAEELR-- 654
S.pneumoniaeD39_PBP2             ---------LERASAMKDSLNLQTTAK--ALEQVSQQSPYPMPSVKDISPGDLAEELR-- 654
S.pneumoniaeNCTC7465_PBP2        ---------LERASAMKDSLNLQTTAK--ALEQVSQQSPYPMPSVKDISPGDLAEELR-- 654
G.adiacensATCC49175_PBP2         ---------MKRSLEYSRLSE--------------------------------------- 595
G.adiacensIS48_PBP2              ---------MKRSLEYSRLSE--------------------------------------- 595
G.adiacensGA01_PBP2              ---------MKRSLEYSRLSE--------------------------------------- 595
G.adiacensKHUD009_PBP2           ---------MKRSLEYSRLSE--------------------------------------- 595
E.faecalisOG1RF_PBP2             ---------MKLALDIKETDPET-TKV--------ASDKVTVADYKNMTPAEALANAK-- 646
E.faeciumDO_PBP2                 ---------MKRAMDFKETEEDSDTET--------KTEKVSVADYRNLEADVAAADAQ-- 636


G.adiacensATCC49175_PBP1B        AGTFK---------------------AENGGTYSISGGMTTDWFKKDFPPMNPFYNFAIG 785
G.adiacensGA01_PBP1B             AGTFK---------------------AENGGTYSISGGMTTDWFKKDFPPMNPFYNFAIG 785
G.adiacensIS48_PBP1B             AGTFK---------------------AENGGTYSISGGMTTDWFKKDFPPMNPFYNFAIG 785
G.adiacensKHUD009_PBP1B          AGTFK---------------------AENGGTYSISGGMTTDWFKKDFPPMNPFYNFAIG 785
S.pneumoniaeD39_PBP1B            PGKVS---------------------V-EGKEVEVTGSTVTSYWANKSGAPATSYRFAIG 773
S.pneumoniaeR6_PBP1B             PGKVS---------------------V-EGKEVEVTGSTVTSYWANKSGAPATSYRFAIG 773
S.pneumoniaeNCTC7465_PBP1B       PGKVS---------------------V-EGKEVEVTGSTVTSYWANKSGAPATSYRFAIG 773
S.pyogenesATCC-BAA-595_PBP1B     PGTVN---------------------V-NGHTFSVGGEMTTSLWSQK-GPGAMTYRFAIG 748
E.faeciumDO_PBP1B                PGKVT---------------------V-NKKSIQTPGKTVESLWAKN-GPKKSAFKFGVG 753
E.faecalisOG1RF_PBP1B            PGSIT---------------------Y-NGAKFNTPGKTTTSYYAKD-GAPQSTYKFGIG 767
G.adiacensATCC49175_PBP2A        ----K----NQNNTLDNFIQDAQKT-------GE------QIW----------------N 704
G.adiacensKHUD009_PBP2A          ----K----NQNNTLDNFIQDAQKT-------GE------QIW----------------N 704
G.adiacensGA01_PBP2A             ----K----NQNNTLDNFIQDAQKT-------GE------QIW----------------N 704
G.adiacensIS48_PBP2A             ----K----NQNNTLDKFIQDAQKT-------GE------QIW----------------N 704
E.faecalisOG1RF_PBP2A            -SLTGGANSNWRDNLKDFGNSVQDGASNFGDTLN------EVG----------------G 705
E.faeciumDO_PBP2A                TGNSNGESGNWQDNLNQYGEQAKEGLKNFGDMVK------EGV----------------Q 707
S.pyogenesATCC-BAA-595_PBP2A     --TGNQSGVDTQSIIDGLRKSAQEASQSLSKAV--------DQ----------------S 760
S.pneumoniaeR6_PBP2A             --VQTNDNSQTDDNLSDIRGRAQSLVDEASRAI--------SD----------------A 713
S.pneumoniaeD39_PBP2A            --VQTNDNSQTDDNLSDIRGRAQSLVDEASRAI--------SD----------------A 713
S.pneumoniaeNCTC7465_PBP2A       --VQTNDNSQTDDNLSDIRGRAQSLVDEASRAI--------SD----------------A 713
G.adiacensKHUD009_PBP1A          PGPRTPANMIATELFVKGSTPT-QQSSNYGITIEGPTGLKATY----------------N 718
G.adiacensIS48_PBP1A             PGPRTPANMIATELFVKGSTPT-QQSSNYGVTIEGPTGLKATY----------------N 718
G.adiacensATCC49175_PBP1A        PGPRTPANMIATELFVKGSTPT-QQSSNYGVTIEGPTGLKATY----------------N 718
G.adiacensGA01_PBP1A             PGPRTPANMIATELFVKGSTPT-QQSSNYGVTIEGPTGLKATY----------------N 718
E.faecalisOG1RF_PBP1A            EVPNV---QVLPSTTSSA--------PQ-------PESSSTV------------------ 704
E.faeciumDO_PBP1A                TARSN---AITPSTTIPS--------SSYVQ---TPGSSTTET----------------T 713
S.pyogenes_ATCC-BAA-595_PBP1A    ASNTDYTNSVYNNLYSNN--------T-------------T------------------ 682
S.pneumoniaeNCTC7465_PBP1A       STWNSPAPQQPPSTESSS--------S-------------S------------------ 673
S.pneumoniaeR6_PBP1A             STWSSPAPQQPPSTESSS--------S-------------S------------------ 673
S.pneumoniaeD39_PBP1A            STWSSPAPQQPPSTESSS--------S-------------S------------------ 673
G.adiacensIS48_PBP2B             ------------------------------------------------------------ 707
G.adiacensGA01_PBP2B             ------------------------------------------------------------ 707
```

```
G.adiacensATCC49175_PBP2B         ------------------------------------------------------------ 707
G.adiacensKHUD009_PBP2B           ------------------------------------------------------------ 707
S.pneumoniaeR6_PBP2B              ------------------------------------------------------------ 685
S.pneumoniaeD39_PBP2B             ------------------------------------------------------------ 685
S.pneumoniaeNCTC7465_PBP2B        ------------------------------------------------------------ 680
E.faecalisOG1RF_PBP2B             ------------------------------------------------------------ 711
E.faeciumDO_PBP2B                 ------------------------------------------------------------ 717
E.faecalisOG1RF_PBP4              ------------------------------------------------------------ 680
E.faeciumDO_PBP5                  ------------------------------------------------------------ 678
S.pyogenesATCC-BAA-595_PBP2       ------RNLV-QPVVLGTGSKIKKVSHQPGQTLT-ENQQVLIL-SDRFVEVPD--MYGWT 703
S.pneumoniaeR6_PBP2               ------RNLV-QPIVVGTGTKIKNSSAEEGKNLA-PNQQVLIL-SDKAEEVPD--MYGWT 703
S.pneumoniaeD39_PBP2              ------RNLV-QPIVVGTGTKIKNSSAEEGKNLA-PNQQVLIL-SDKAEEVPD--MYGWT 703
S.pneumoniaeNCTC7465_PBP2         ------RNLV-QPIVVGTGTKIKNSSAEEGKNLA-PNQQVLIL-SDKAEEVPD--MYGWT 703
G.adiacensATCC49175_PBP2          ------------------------------------------------------------ 595
G.adiacensIS48_PBP2               ------------------------------------------------------------ 595
G.adiacensGA01_PBP2               ------------------------------------------------------------ 595
G.adiacensKHUD009_PBP2            ------------------------------------------------------------ 595
E.faecalisOG1RF_PBP2              ------VNGV-DPVIIGDGEKIKKQSTPSGQTLM-PNQKLILI-TNGTNYMPD--LTGWS 695
E.faeciumDO_PBP2                  ------KSGL-QPVVIGNGKKVQKQSTANGDQLI-SGEKLILY-TGGDKLMPD--VTGWS 685


G.adiacensATCC49175_PBP1B         ATPEEMNNFWNKATPKKEEKKDTKKDEKKDEKKDEKKNEKKEETTQSPSTQSQ------- 838
G.adiacensGA01_PBP1B              ATPEEMNNFWNKATPKKEEKKDKKKDEKKDEKKDEKKNEKKEETTQSPSTQSQ------- 838
G.adiacensIS48_PBP1B              ATPEEMNNFWNKATPKKEEKKDTKKDEKKNEKKDEKKNEKKEETTQSPSTQNQ------- 838
G.adiacensKHUD009_PBP1B           ATPEEMNNFWNKATPKKEEKKDTKKDEKKDEKKEEKKNEKKEETTQSPSTQSQ------- 838
S.pneumoniaeD39_PBP1B             GSDADYQNAWSSIVGSLPTPSSSSSSSSSSSDS---SNSS----TTRPSSSR-------- 818
S.pneumoniaeR6_PBP1B              GSDADYQNAWSSIVGSLPTPSSSSSSSSSSSDS---SNSS----TTRPSSSR-------- 818
S.pneumoniaeNCTC7465_PBP1B        GSDADYQNAWSSIVGSLPTPSSSSSSSSSSSDS---SNSS----TTRPSSSR-------- 818
S.pyogenesATCC-BAA-595_PBP1B      GTDADYQKAWGNFGFRKN------------------------------------------ 766
E.faeciumDO_PBP1B                 GTDENYTDYWNTASAYARANQATKESDEKDD----------------------------- 784
E.faecalisOG1RF_PBP1B             GTDSNYASYWGNLAPRATTNNNNNNNNKNNDNK---KNN--------------------- 803
G.adiacensATCC49175_PBP2A         QLQEWGKNIWNKFNNR-------------------------------------------- 720
G.adiacensKHUD009_PBP2A           QLQEWGKNIWKKFNNR-------------------------------------------- 720
G.adiacensGA01_PBP2A              QLQEWGKNIWNKFKNR-------------------------------------------- 720
G.adiacensIS48_PBP2A              QLQEWGRNIWNKFNNR-------------------------------------------- 720
E.faecalisOG1RF_PBP2A             QIKDGAKKVKDKIEGIFGGLLGN------------------------------------- 728
E.faeciumDO_PBP2A                 GIGEAAKDLWRKYQGE-------------------------------------------- 723
S.pyogenesATCC-BAA-595_PBP2A      GLRDKAQSIWKEIVDYFR------------------------------------------ 778
S.pneumoniaeR6_PBP2A              KIKEKAQTIWDSIVNLFR------------------------------------------ 731
S.pneumoniaeD39_PBP2A             KIKEKAQTIWDSIVNLFR------------------------------------------ 731
S.pneumoniaeNCTC7465_PBP2A        KIKEKAQTIWDSIVNLFR------------------------------------------ 731
G.adiacensKHUD009_PBP1A           KEKKELTVTWDRYNNTGQGTPQFKVTANGQSQTVTGNS-VKFQNITGPSVSVSIVVTVGR 777
G.adiacensIS48_PBP1A              KEKKELTVTWDRYNNTGQGTPQFKVTANGQSQTVTGNS-VKFQNITGPSVSVSIVVTVGR 777
```

```
G.adiacensATCC49175_PBP1A          KEKKELTVTWDRYNNTGQGTPQFKVTANGQSQTVTGNS-VKFQNITGPSVSVSIVVTVGR 777
G.adiacensGA01_PBP1A               KEKKELTVTWDRYNNTGQGTPQFKVTANGQSQTVTGNS-VKFQNITGPSVSVSIVVTVGR 777
E.faecalisOG1RF_PBP1A              ---------------ESSSTKEAESSSSSSSESAPSSSEAPP-STEQPASSS-------- 740
E.faeciumDO_PBP1A                  TQ-------------SSSSTSQSESTAESSKESTTAETSEPASSTTVPSSSS-------- 752
S.pyogenes_ATCC-BAA-595_PBP1A      ---------------TASSQTTSDDTSSSNDTSNST---NTDNNG-----S--------- 710
S.pneumoniaeNCTC7465_PBP1A         ---------------SDSSTSQSSSTTPSTNNSTTT---NPNNNT-----QQ-------- 702
S.pneumoniaeR6_PBP1A               ---------------SDSSTSQSSSTTPSTNNSTTT---NPNNNT-----QQ-------- 702
S.pneumoniaeD39_PBP1A              ---------------SDSSTSQSSSTTPSTNNSTTT---NPNNNT-----QQ-------- 702
G.adiacensIS48_PBP2B               ------------------------------------------------------------ 707
G.adiacensGA01_PBP2B               ------------------------------------------------------------ 707
G.adiacensATCC49175_PBP2B          ------------------------------------------------------------ 707
G.adiacensKHUD009_PBP2B            ------------------------------------------------------------ 707
S.pneumoniaeR6_PBP2B               ------------------------------------------------------------ 685
S.pneumoniaeD39_PBP2B              ------------------------------------------------------------ 685
S.pneumoniaeNCTC7465_PBP2B         ------------------------------------------------------------ 680
E.faecalisOG1RF_PBP2B              ------------------------------------------------------------ 711
E.faeciumDO_PBP2B                  ------------------------------------------------------------ 717
E.faecalisOG1RF_PBP4               ------------------------------------------------------------ 680
E.faeciumDO_PBP5                   ------------------------------------------------------------ 678
S.pyogenesATCC-BAA-595_PBP2        KSNVKTFAKWTGIDISFKGTDSGRVMK----QSVDVGKSLKK--------IKKMTITLGD 751
S.pneumoniaeR6_PBP2                KETAETLAKWLNIELEFQGSGS-TVQK----QDVRANTAIKD--------IKKITLTLGD 750
S.pneumoniaeD39_PBP2               KETAETLAKWLNIELEFQGSGS-TVQK----QDVRANTAIKD--------IKKITLTLGD 750
S.pneumoniaeNCTC7465_PBP2          KETAETFAKWLNIELEFQGSGS-TVQK----QDVRANTAIKD--------IKKITLTLGD 750
G.adiacensATCC49175_PBP2           ------------------------------------------------------------ 595
G.adiacensIS48_PBP2                ------------------------------------------------------------ 595
G.adiacensGA01_PBP2                ------------------------------------------------------------ 595
G.adiacensKHUD009_PBP2             ------------------------------------------------------------ 595
E.faecalisOG1RF_PBP2               KSDVTKFGDLLGLTVEFKGEGY--VTK----QSIAAETEITE---------KKLTVTLEG 740
E.faeciumDO_PBP2                   KADIMKLGKILGIEVSFDGDGY--CVK----QELAPYEKITE---------DKLSFTLEE 730


G.adiacensATCC49175_PBP1B          --------------------------------TTRSN----------------------- 843
G.adiacensGA01_PBP1B               --------------------------------TTRSN----------------------- 843
G.adiacensIS48_PBP1B               --------------------------------TTRSN----------------------- 843
G.adiacensKHUD009_PBP1B            --------------------------------TTRSN----------------------- 843
S.pneumoniaeD39_PBP1B              --------------------------------ARR------------------------- 821
S.pneumoniaeR6_PBP1B               --------------------------------ARR------------------------- 821
S.pneumoniaeNCTC7465_PBP1B         --------------------------------ARR------------------------- 821
S.pyogenesATCC-BAA-595_PBP1B       ------------------------------------------------------------ 766
E.faeciumDO_PBP1B                  ------------------------------------------------------------ 784
E.faecalisOG1RF_PBP1B              ------------------------------------------------------------ 803
G.adiacensATCC49175_PBP2A          ------------------------------------------------------------ 720
G.adiacensKHUD009_PBP2A            ------------------------------------------------------------ 720
```

```
G.adiacensGA01_PBP2A              ------------------------------------------------------------ 720
G.adiacensIS48_PBP2A              ------------------------------------------------------------ 720
E.faecalisOG1RF_PBP2A             ------------------------------------------------------------ 728
E.faeciumDO_PBP2A                 ------------------------------------------------------------ 723
S.pyogenesATCC-BAA-595_PBP2A      ------------------------------------------------------------ 778
S.pneumoniaeR6_PBP2A              ------------------------------------------------------------ 731
S.pneumoniaeD39_PBP2A             ------------------------------------------------------------ 731
S.pneumoniaeNCTC7465_PBP2A        ------------------------------------------------------------ 731
G.adiacensKHUD009_PBP1A           NSSDPITNEFQI-----------EQPTTTQEQTTRQSETTTDNNHRQNNNSNNEQTTQER 826
G.adiacensIS48_PBP1A              NSSDPITNEFQI-----------EQPTTTQEQTTRQSETTTENNRRN--DSNNEQTTQER 824
G.adiacensATCC49175_PBP1A         NSSDPITNEFQI-----------EQPTTTQEQTTRQSETTTENNRRN--DSNNEQTTQER 824
G.adiacensGA01_PBP1A              NSSDPITNEFQI-----------EQPTTTQEQTTRQSETTTENNRRN--DSNNEQTTQER 824
E.faecalisOG1RF_PBP1A             SAEQPATSEQPPEPSSSSSQEPPQPPESSSKPDENKA--A-------------------- 778
E.faeciumDO_PBP1A                 EES--------STP------SSSAPPASSSEPATSGA--DAANDHTPS------SSTSAS 790
S.pyogenes_ATCC-BAA-595_PBP1A     ---HPSTDDKKTTH---------------------------------------------- 721
S.pneumoniaeNCTC7465_PBP1A        SNTTPEQQNQNPQPA---------QP---------------------------------- 719
S.pneumoniaeR6_PBP1A              SNTTPDQQNQNPQPA---------QP---------------------------------- 719
S.pneumoniaeD39_PBP1A             SNTTPDQQNQNPQPA---------QP---------------------------------- 719
G.adiacensIS48_PBP2B              ------------------------------------------------------------ 707
G.adiacensGA01_PBP2B              ------------------------------------------------------------ 707
G.adiacensATCC49175_PBP2B         ------------------------------------------------------------ 707
G.adiacensKHUD009_PBP2B           ------------------------------------------------------------ 707
S.pneumoniaeR6_PBP2B              ------------------------------------------------------------ 685
S.pneumoniaeD39_PBP2B             ------------------------------------------------------------ 685
S.pneumoniaeNCTC7465_PBP2B        ------------------------------------------------------------ 680
E.faecalisOG1RF_PBP2B             ------------------------------------------------------------ 711
E.faeciumDO_PBP2B                 ------------------------------------------------------------ 717
E.faecalisOG1RF_PBP4              ------------------------------------------------------------ 680
E.faeciumDO_PBP5                  ------------------------------------------------------------ 678
S.pyogenesATCC-BAA-595_PBP2       ------------------------------------------------------------ 751
S.pneumoniaeR6_PBP2               ------------------------------------------------------------ 750
S.pneumoniaeD39_PBP2              ------------------------------------------------------------ 750
S.pneumoniaeNCTC7465_PBP2         ------------------------------------------------------------ 750
G.adiacensATCC49175_PBP2          ------------------------------------------------------------ 595
G.adiacensIS48_PBP2               ------------------------------------------------------------ 595
G.adiacensGA01_PBP2               ------------------------------------------------------------ 595
G.adiacensKHUD009_PBP2            ------------------------------------------------------------ 595
E.faecalisOG1RF_PBP2              TE---------------------------------------------------------- 742
E.faeciumDO_PBP2                  ------------------------------------------------------------ 730


G.adiacensATCC49175_PBP1B         ---  843
G.adiacensGA01_PBP1B              ---  843
```

```
G.adiacensIS48_PBP1B              --- 843
G.adiacensKHUD009_PBP1B           --- 843
S.pneumoniaeD39_PBP1B             --- 821
S.pneumoniaeR6_PBP1B              --- 821
S.pneumoniaeNCTC7465_PBP1B        --- 821
S.pyogenesATCC-BAA-595_PBP1B      --- 766
E.faeciumDO_PBP1B                 --- 784
E.faecalisOG1RF_PBP1B             --- 803
G.adiacensATCC49175_PBP2A         --- 720
G.adiacensKHUD009_PBP2A           --- 720
G.adiacensGA01_PBP2A              --- 720
G.adiacensIS48_PBP2A              --- 720
E.faecalisOG1RF_PBP2A             --- 728
E.faeciumDO_PBP2A                 --- 723
S.pyogenesATCC-BAA-595_PBP2A      --- 778
S.pneumoniaeR6_PBP2A              --- 731
S.pneumoniaeD39_PBP2A             --- 731
S.pneumoniaeNCTC7465_PBP2A        --- 731
G.adiacensKHUD009_PBP1A           RNR 829
G.adiacensIS48_PBP1A              RNR 827
G.adiacensATCC49175_PBP1A         RNR 827
G.adiacensGA01_PBP1A              RNR 827
E.faecalisOG1RF_PBP1A             --- 778
E.faeciumDO_PBP1A                 GNR 793
S.pyogenes_ATCC-BAA-595_PBP1A     --- 721
S.pneumoniaeNCTC7465_PBP1A        --- 719
S.pneumoniaeR6_PBP1A              --- 719
S.pneumoniaeD39_PBP1A             --- 719
G.adiacensIS48_PBP2B              --- 707
G.adiacensGA01_PBP2B              --- 707
G.adiacensATCC49175_PBP2B         --- 707
G.adiacensKHUD009_PBP2B           --- 707
S.pneumoniaeR6_PBP2B              --- 685
S.pneumoniaeD39_PBP2B             --- 685
S.pneumoniaeNCTC7465_PBP2B        --- 680
E.faecalisOG1RF_PBP2B             --- 711
E.faeciumDO_PBP2B                 --- 717
E.faecalisOG1RF_PBP4              --- 680
E.faeciumDO_PBP5                  --- 678
S.pyogenesATCC-BAA-595_PBP2       --- 751
S.pneumoniaeR6_PBP2               --- 750
S.pneumoniaeD39_PBP2              --- 750
S.pneumoniaeNCTC7465_PBP2         --- 750
```

```
G.adiacensATCC49175_PBP2           --- 595
G.adiacensIS48_PBP2                --- 595
G.adiacensGA01_PBP2                --- 595
G.adiacensKHUD009_PBP2             --- 595
E.faecalisOG1RF_PBP2               --- 742
E.faeciumDO_PBP2                   --- 730
```

Figure S1. PBPs amino acid sequences multi alignment by Clustal omega.
